# Synthesis, in vitro potency of inhibition, enzyme kinetics and in silico studies of quinoline-based α-glucosidase inhibitors

**DOI:** 10.1038/s41598-023-50711-2

**Published:** 2024-01-04

**Authors:** Minoo Khalili Ghomi, Navid Dastyafteh, Mohammad Nazari Montazer, Milad Noori, Somayeh Mojtabavi, Mohammad Ali Faramarzi, Seyedeh Mahdieh Hashemi, Mohammad Mahdavi

**Affiliations:** 1 Endocrinology and Metabolism Research Center, Endocrinology and Metabolism Clinical Sciences Institute, Tehran University of Medical Sciences, Tehran, Iran; 2 Department of Pharmaceutical Biotechnology, Faculty of Pharmacy and Biotechnology Research Center, Tehran University of Medical Sciences, Tehran, Iran; 3 Department of Medicinal Chemistry and Pharmaceutical Sciences Research Center, Faculty of Pharmacy, Mazandaran University of Medical Sciences, Sari, Iran

**Keywords:** Computational biology and bioinformatics, Drug discovery, Endocrinology, Chemistry

## Abstract

Diabetes mellitus is a multifactorial global health disorder that is rising at an alarming rate. One effective therapeutic approach for controlling hyperglycemia associated with type-2 diabetes is to target α-glucosidase, which catalyzes starch hydrolysis in the intestine. In an attempt to find potential α-glucosidase inhibitors, a series of twenty new quinoline linked benzothiazole hybrids (**8a–t**) were synthesized in good yields from suitable reaction procedures and their chemical structures were analyzed by ^1^HNMR, ^13^CNMR, IR, and ESI–MS analysis. The synthesized derivatives further screened for their activity against α-glucosidase. Among them, compounds **8b**, **8h**, **8n** and **8o** exhibited remarkable α-glucosidase inhibitory activity with IC_50_ values ranging from 38.2 ± 0.3 to 79.9 ± 1.2 µM compared with standard drug acarbose (IC_50_ = 750.0 ± 2.0 µM). Enzyme kinetic studies of the most active compound (**8h**) indicated a non-competitive inhibition with K_i_ value of 38.2 µM. Moreover, the homology modeling, molecular docking and molecular dynamics simulation studies were conducted to reveal key interactions between the most active compound **8h** and the targeted enzyme. These results are complementary to the experimental observations. In order to predict the druggability of the novel derivatives, the pharmacokinetic properties were also applied. These findings could be useful for the design and development of new α-glucosidase inhibitors.

## Introduction

**Figure 1:**
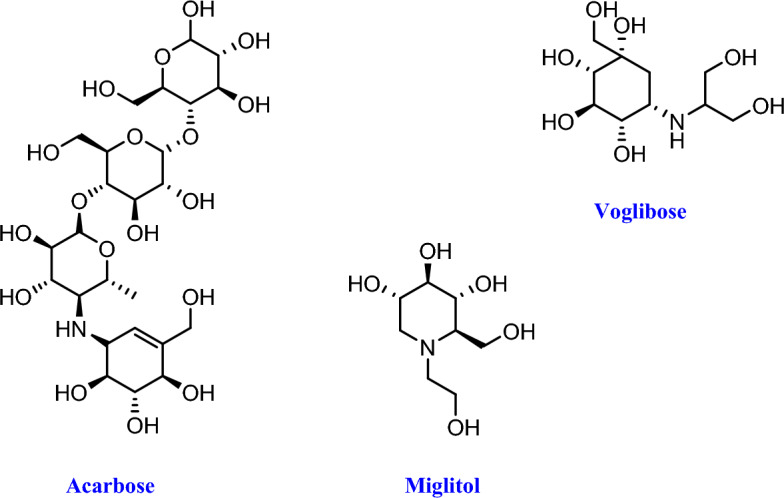
FDA approved AGIs for the treatment of type 2 diabetes mellitus.

Nowadays, as physical activity reduction and obesity increase lead to change lifestyle, the worldwide outbreak of diabetes is taking on pandemic dimensions, and the number of people were suffering from diabetes is estimated to extend to 439 million by 2030^1^. Type 2 diabetes is an endocrine chronic metabolic disorder widely spread, characterized by abnormal levels of glucose in the blood stream. The currently anti-diabetic drugs are classified based on distinct mechanistic groups such as insulin secretagogues (sulfonylureas), insulin sensitizers (biguanides and thiazolidinediones), insulin mimetics (glucagon-like peptide analogues and agonists), α-glucosidase inhibitors (miglitol, acarbose, voglibose), and DPP-4 inhibitors^2^. Among these classes, α-glucosidase inhibitors (AGIs) indicated substantial potential for preventing an association between postprandial hyperglycemia and macrovascular complications in diabetic subjects^3^. Moreover, α-glucosidase removes viral glycoproteins and its inhibitors may be beneficial in the viral infection treatment.* α*-Glucosidase, an *exo-*type glycosidase enzyme which found on the brush borders of the small intestine, converts carbohydrates into monosaccharides in order to prepare energy for regular function of human body^4^. *α*-Glucosidase inhibition not only defers the absorption of carbohydrates but also reduces the summit of postprandial blood glucose. AGIs are exclusively beneficial for decreasing postprandial hyperglycemia and glycosylated hemoglobin levels and also reduce postprandial insulin concentration. They also diminish glucose variability throughout the day, compared with oral antihyperglycemic drugs. They, although, do not affect fasting insulin and serum triglyceride concentrations. The FDA approves AGIs for the treatment of type 2 diabetes mellitus, that only acarbose, miglitol and voglibose are advanced to clinical level (Fig. 1)^5^. Acarbose (received its first FDA approval in 1995) has been shown to decrease body weight in a worldwide observational study. It has been proven that acarbose increases life expectancy in patients with type 2 diabetes mellitus and reduces the risk of cardiovascular events development in individuals with impaired glucose tolerance. Miglitol is a second generation AGI, followed in 1996. It is a derivative of 1-desoxynojirimycin, and binds reversibly to the brush border α-glucosidase enzymes. In contrast to its parent drug (acarbose), miglitol is almost completely absorbed in the small intestine. These are the only two AGIs approved for the United States market, although another AGI, voglibose, was approved by the Pharmaceuticals and Medical Devices Agency in Japan^6^.

Synthetic and natural products containing quinoline structure representing diverse pharmacological activities such as anti-biotic^7^, anti-cancer^8^, anti-fungal^9^, anti-malarial^10^, anti-bacterial^11^, anti-HIV^12^, anti-rheumatoid arthritis^13^, anti-tuberculosis^14^, anti-diabetic^15^, anti-oxidant^16^, anti-inflammatory^17^. Introducing chloroquine as anti-malaria began a new era of quickly developing drugs like pamaquine, ciprofloxacin, camptothecin, topotecan. On the other hand, thiazole moiety consisting both nitrogen and sulfur is also well known due to their wide spectrum biological potential including anti-inflammatory^18^, antitumor^19^, antiviral^20^, antifungal^21^ and antibacterial activities^22^. Moreover, abafungin, ritonavir, tiazofurin, niridazole and nitazoxanide possess this widely used scaffold.

**Figure 2:**
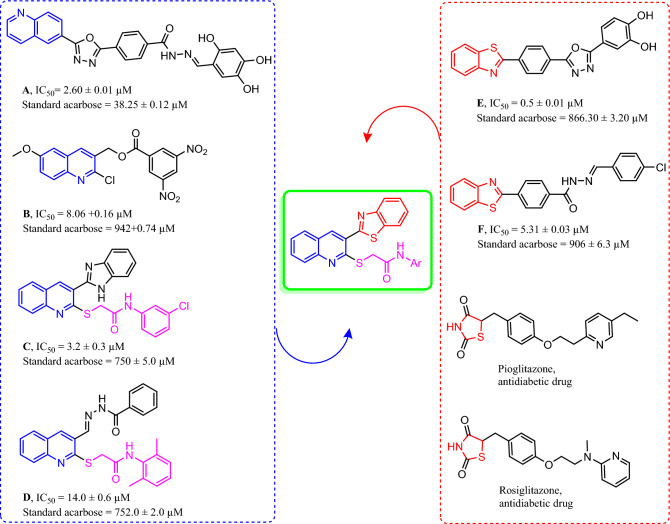
Design strategy of novel α-glucosidase inhibitors.

Quinoline pharmacophore, which has strong interactions with the active site of α-glucosidase, is well known for its inhibitory activities (Compounds **A–D**). Also, thiazoles and benzothiazoles (Compounds **E, F**) has been represented as α-glucosidase inhibitors^16,23–27^ for their ability of providing leads with easy synthetic protocol and structural diversity that make ideal framework in antidiabetic drug discovery (pioglitazone and rosiglitazone) (Fig. 2).

In continuing our research to find new pharmacophore for the potent α-glucosidase inhibitors and due to develop more effective α-glucosidase inhibitors by combining the quinoline and benzothiazole moieties in one molecule, we report the synthesis of novel quinolone tagged benzothiazole derivatives, the in vitro α-glucosidase inhibition and a molecular dynamic simulation study was also performed to represent a structural rational of the obtained inhibitory potencies.

## Chemistry

**Scheme 1:**
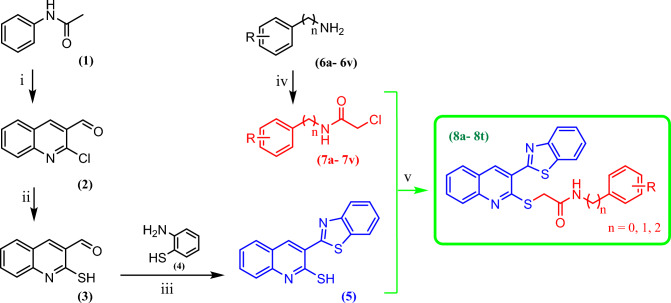
(i) (**a**) POCl_3_, DMF, 0 °C, (**b**) 80 °C, 12 h; (ii) Na_2_S, DMF, room temperature; (iii) sodium metabisulfite, EtOH, reflux; (iv) chloroacetyl chloride, DMF, room temperature, 12 h; (v) Acetone, K_2_CO_3_, room temperature, 4 h.

The title compounds, 2-((3-(benzo[d]thiazol-2-yl)quinolin-2-yl)thio)-*N*-benzylacetamide derivatives (**8a–v**), were synthesized according to Scheme 1. As shown, the key intermediate **5**, 3-(benzo[d]thiazol-2-yl)quinoline-2-thiol, was prepared in three steps. Firstly, to the stirred phosphoryl chloride in DMF at 0–5 °C, acetanilide was added after warming to the room temperature and the resulting mixture was heated for 12 h at 80–90 °C. The obtained 2-chloroquinoline-3-carbaldehyde (**2**), was entered to the next stage of the reaction without any purification. Secondly, the mixture of compound **2** and sodium sulfide was left to stir for 2 h at room temperature in DMF. Then, the reaction mixture was poured into crushed ice and acidified with acetic acid. The obtained 2-mercaptoquinoline-3-carbaldehyde (**3**) was further recrystallized in ethanol. In the third step, the latter intermediate (**3**) and 2-aminobenzenethiol **4** were dissolved and stirred at room temperature. Along with adding sodium metabisulfite, the resulted mixture is allowed to reflux for about 12 h. After reaction completion, the mixture was precipitated in ice water, filtered, and purified by recrystallization in ethanol to afford compound **5**. On the other hand, the chloroacetyl chloride was added to amine derivatives **6** in DMF, while they were cooled to 0 °C. Then the mixture was left to stir at room temperature for 12 h, which was resulted acetamide derivatives **7**. Finally, the 2-chloro-*N*-substituted acetamide derivatives (**7**) were reacted with 3-(benzo[d]thiazol-2-yl)quinoline-2-thiol (**3**) in dry acetone and K_2_CO_3_ at room temperature for 4 h, filtered and the solid product was recrystallized by ethanol to give title products (**8**).

### In vitro α-glucosidase inhibitory activity and SAR analysis

In order to discover potent α-glucosidase inhibitors and to investigate the structure–activity relationships, all synthesized derivatives **8a–t** were screened to assess their potential inhibitory activities. Half maximal inhibitory concentration of the potency of the compounds in inhibiting α-glucosidase, in comparison with acarbose as the reference drug, are shown in Table 1. Based on the obtained IC_50_s, all of tested compounds demonstrated potent inhibitory activities (IC_50_s = 38.2 ± 0.3—384.3 ± 0.3 µM) against α-glucosidase compared with acarbose (IC_50_ = 750.0 ± 2.0 µM). The *para*-fluorophenyl (**8h**) and *para*-tolyl (**8c**) derivatives are the most active and weakest ones among all synthesized compounds, respectively.

**Table 1:** α-Glucosidase inhibitory activity of compounds **8a–t**.


Entry	Compounds	Ar	n	IC_50_ (µM)^a^	Concentrations of precipitation (µM)	Glide score (Kcal/mol)
**1**	**8a**		0	299.0 ± 0.1	> 90	− 5.114
**2**	**8b**		0	79.9 ± 1.2	> 90	− 6.219
**3**	**8c**		0	384.3 ± 0.3	> 90	− 5.527
**4**	**8d**		0	194.4 ± 0.8	> 90	− 5.920
**5**	**8e**		0	130.4 ± 0.5	> 90	− 5.697
**6**	**8f**		0	231.6 ± 0.7	> 90	− 5.481
**7**	**8g**		0	198.5 ± 0.9	> 90	− 5.721
**8**	**8h**		0	38.2 ± 0.3	> 90	− 6.842
**9**	**8i**		0	118.2 ± 1.4	> 90	− 5.110
**10**	**8j**		0	111.7 ± 1.1	> 90	− 5.261
**11**	**8k**		0	145.4 ± 1.5	> 90	− 5.432
**12**	**8l**		0	182.7 ± 1.0	> 90	− 5.411
**13**	**8m**		0	198.9 ± 1.5	> 90	− 5.121
**14**	**8n**		0	65.9 ± 1.0	> 90	− 6.231
**15**	**8o**		0	47.2 ± 0.6	> 90	− 6.658
**16**	**8p**		0	139.8 ± 1.0	> 90	− 6.623
**17**	**8q**		1	255.6 ± 0.3	> 90	− 5.117
**18**	**8r**		1	161.4 ± 0.4	> 90	− 5.223
**19**	**8s**		1	130.1 ± 0.7	> 90	− 5.518
**20**	**8t**		2	222.7 ± 0.9	> 90	− 5.020
	**Acarbose**			750.0 ± 2.0		− 7.171

^a^Data are the mean of three-independent determinations of triplicate samples and represented in terms of mean ± SD.

A thoroughly looking at the structures and activities in Table 1 indicated that all compounds showed higher inhibitory activity against α-glucosidase compared to acarbose. The most potent compounds were **8b**, **8h**, **8n** and **8o** with IC_50_ values ranging from 38.2 ± 0.3 to 79.9 ± 1.2 µM and the weakest one was compound **8c** with IC_50_ value of 384.3 ± 0.3 µM. Moreover, the unsubstituted analogs bearing phenyl (**8a**), benzyl (**8q)** and phenethyl (**8t**) moieties showed weak activity against α-glucosidase. Interestingly, most of the potent derivatives bear an electron-withdrawing groups and also substitution in *ortho* and *para* positions. Although the weakest compound had a *para*-tolyl moiety (**8c**), the *para*-ethyl substitution (**8d**) was better tolerated compared to it. However, compound **8b** with *ortho*-tolyl group showed fivefold more potency compared to *para*-isomer (**8c**). Moreover, a significant decrease in potency was observed when the second methyl group were introduced to the compound **8b**.

Comparison between halogenated derivatives (**8g–l**) revealed that the presence of halogen on the *para* position remarkably improved the activity, since the *para*-fluoro substitution on phenyl ring in compound **8h** increased the inhibitory activity by 10-fold more than unsubstituted phenyl. Moreover, fluorine introduction significantly improved the inhibitory activity between other halogenated ones. It is worth to mention that the *para*-fluoro substitution on the benzyl ring (**8s**) showed less activity about threefold compared to phenyl one. The SAR analysis demonstrated that the different chloro-substituted derivatives were potent and also the *meta* derivative was the most one among them. Furthermore, by comparing compounds **8c** and **8r** in the phenyl and benzyl series, we can conclude that introducing methyl group to benzyl ring could be beneficial for inhibitory activity compared to phenyl ring. Substitution phenyl ring in compound **8a** with α-naphthyl (**8p**), benzyl (**8q**) and phenethyl (**8t**) groups can slightly restore the inhibitory activity. Compounds **8m–o** containing nitro group were remarkably potent. Among them, the derivatives **8n** bearing *ortho*-nitrotolyl substituent (IC_50_ = 65.9 ± 1.0 µM) and **8o** containing 5-nitrothiazolyl moiety (IC_50_ = 47.2 ± 0.6 µM) exhibited superior activity compared to the **8m** with *para*-nitrophenyl substitution (IC_50_ = 198.9 ± 1.5 µM). Although the *para*-tolyl derivative **8c** was the weakest compound, insertion of the nitro group on the *ortho* position of phenyl ring resulted in compound **8n** with very potent activity (IC_50_ = 65.9 ± 1.0 µM).

It can be concluded that the compounds containing electron-withdrawing groups were more potent than the compounds bearing electron-donating ones. However, this substitution in *ortho* and *para* position is more favorable.

**Figure 3:**
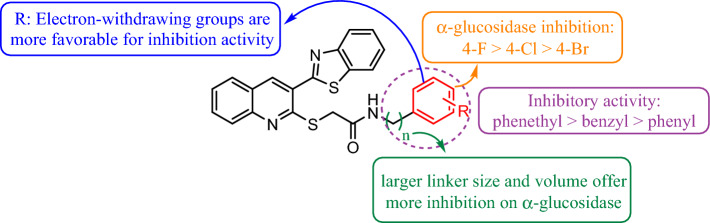
Summary of structure–activity relationship of compounds **8a–t**.

Overall, these obtained biological results revealed that the effect of halogen substituent dramatically impacts on the α-glucosidase inhibition. Importantly, our study demonstrates that the aryl part should be optimized by varying the halogen substituent, and even the larger groups than phenyl ring. The summary of SAR is presented in Fig. 3.

The SAR analysis, informed by the molecular docking study, aligns with the outcomes of the enzyme assay. The compound with the highest glide score, compound **8h**, registered at − 6.842 kcal/mol, coinciding with its status as the most efficacious compound in the enzyme assay. Compounds with para-substitutions consistently showed elevated docking scores, particularly those with electron-withdrawing para substituents. In the context of halogen para-substitutions, the trend appears as F > Cl > Br, evident from the glide scores of compounds **8h**, **8k**, and **8i**, which were − 6.842, − 5.432, and − 5.411 kcal/mol, respectively. It's also evident that compounds with unsubstituted aryl groups, such as **8a**, **8q**, and **8t**, showcased the lowest glide scores, coming in at − 5.114, − 5.117, and − 5.020 kcal/mol, respectively. Notably, Acarbose exhibited a superior glide docking score in comparison to all synthesized compounds. This could be attributed to its expansive molecular surface and its capacity to form numerous hydrophobic interactions. Subsequent sections will delve deeper into a comparison of Acarbose and compound **8h** properties.

### Enzyme kinetic study

**Figure 4:**
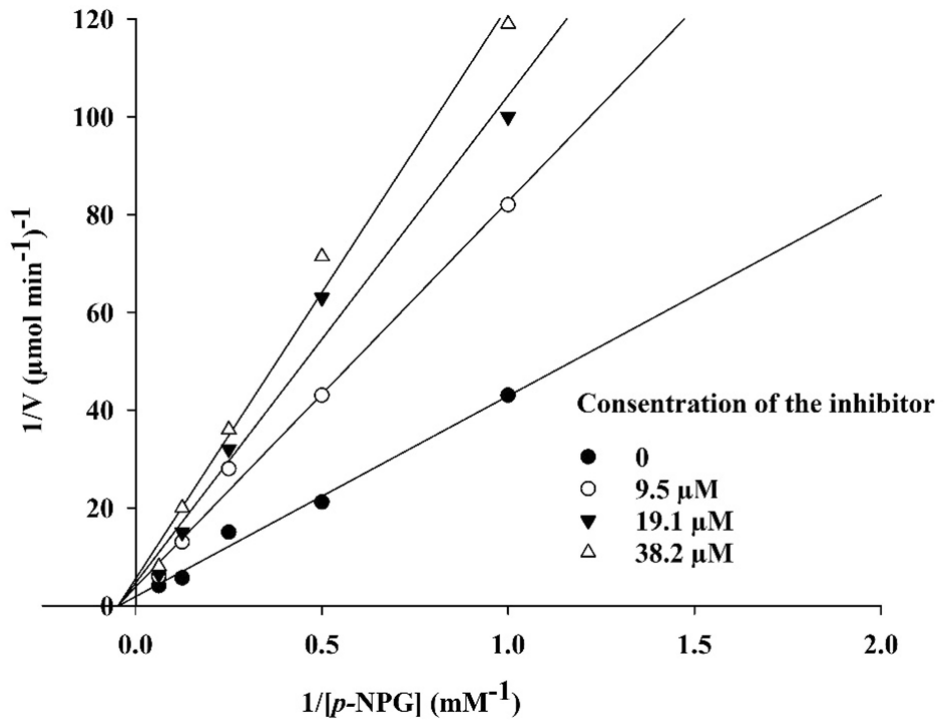
The Lineweaver–Burk plot in the absence and presence of different concentrations of **8h.**

Regarding to Fig. 4, the Lineweaver–Burk plot indicated a non-competitive inhibition for compound **8h**. The results show that decreased efficacy of the α-glucosidase enzyme was obtained by binding of **8h** to an allosteric site, which is differ from the active site; where the acarbose binds. Furthermore, the plot of the *K*_m_ versus different concentrations of inhibitor gave an estimate of the inhibition constant, *K*_i_ value of 38.2 μM.

### Homology modeling

**Figure 5:**
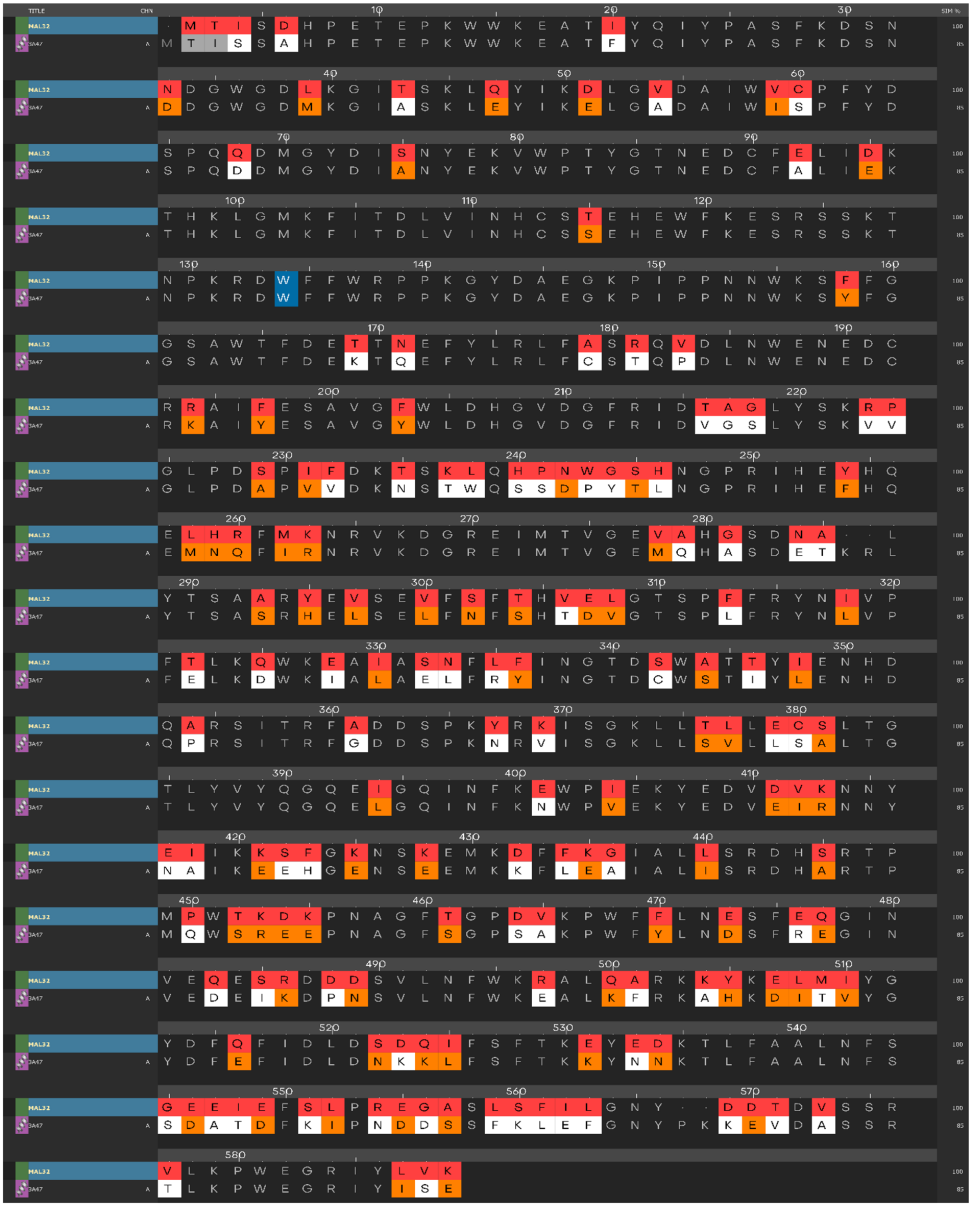
Alignment of *S. cerevisiae* α-glucosidase and *S. cerevisiae* isomaltase (PDB: 3A47) sequences. The different residues from the template are highlighted using colors, with orange representing similar residues and white representing dissimilar residues.

In order to conduct the enzyme assay, the Saccharomyces cerevisiae α-glucosidase enzyme (EC. 3. 2. 1. 20) have been used. However, since the 3-D crystallographic structure of this enzyme is not available in public databases, a new homology model generated using the protein sequence obtained from uniport.org^28^. To create the model, the structure of *S. cerevisiae* isomaltose (PDB: 3A47) utilized as a template due to its high sequence similarity with the Saccharomyces cerevisiae α-glucosidase (85% similarity). The alignment of the sequences is presented in Fig. 5.

In order to verify the correctness of modeled enzyme the Errad analysis have been conducted which showed the modeled protein with the Verification of protein structure by ERRAT analysis exhibited an overall quality factor of 93.892 (Fig. 4), which depicts a good 3D structure of the predicted model. In addition to this, it also examines the statistics of nonbonded atomic interactions. It is the graph of the error value function plotted against the position of a nine-residue sliding window, calculated by comparing data from highly refined structures with each other.

**Figure 6:**
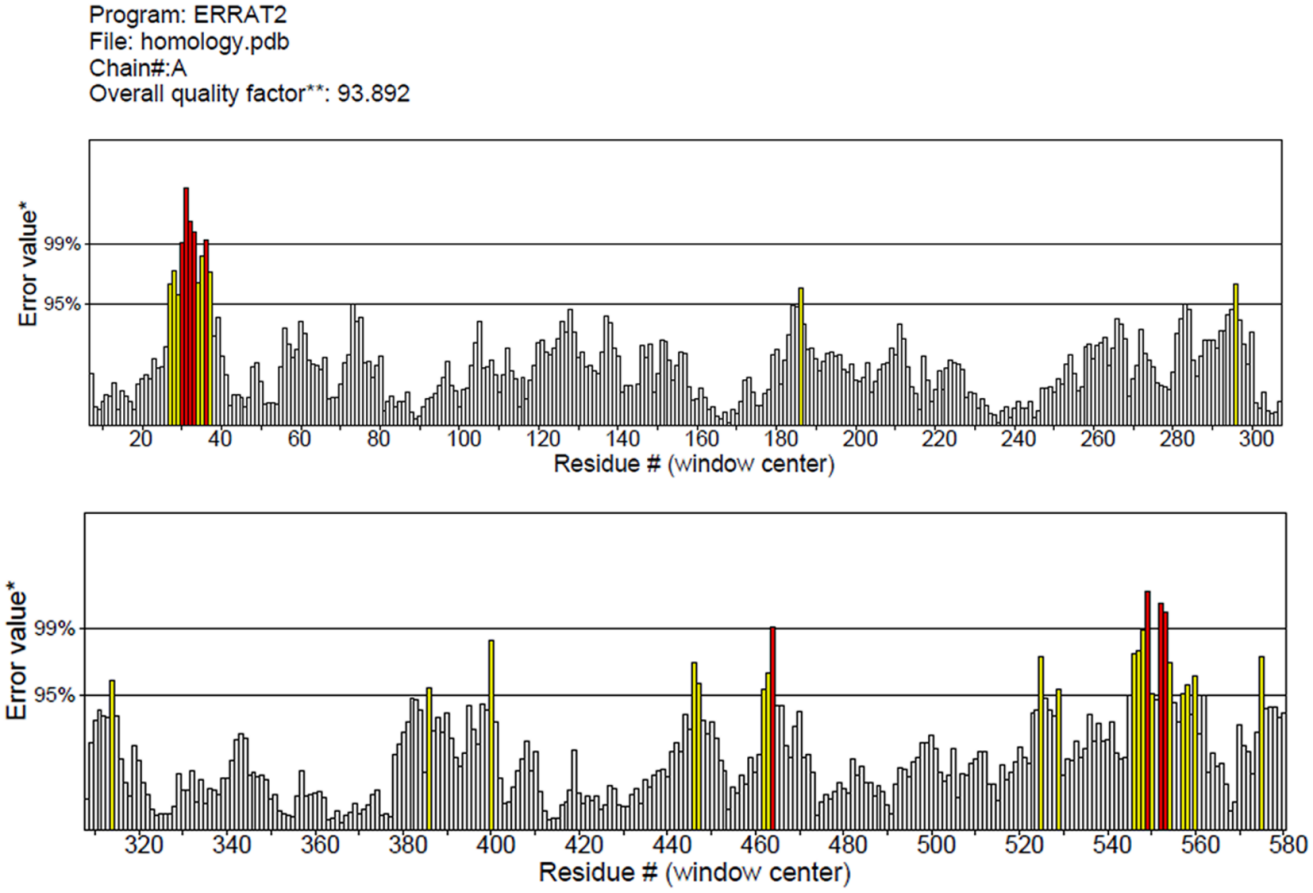
The ERRAT plot of the modelled Enzyme.

To ensure the accuracy of the modeled enzyme, the ERRAT analysis was performed^29^. The results of the ERRAT analysis indicated that the modeled protein had an overall quality factor of 93.892, as shown in Fig. 6. This high-quality factor suggests that the predicted model has a good 3D structure. Additionally, the ERRAT analysis also evaluated the statistics of nonbonded atomic interactions. This analysis involved plotting the error value function against the position of a nine-residue sliding window. The data used for this comparison was obtained from highly refined structures".

### Docking study

**Figure 7:**
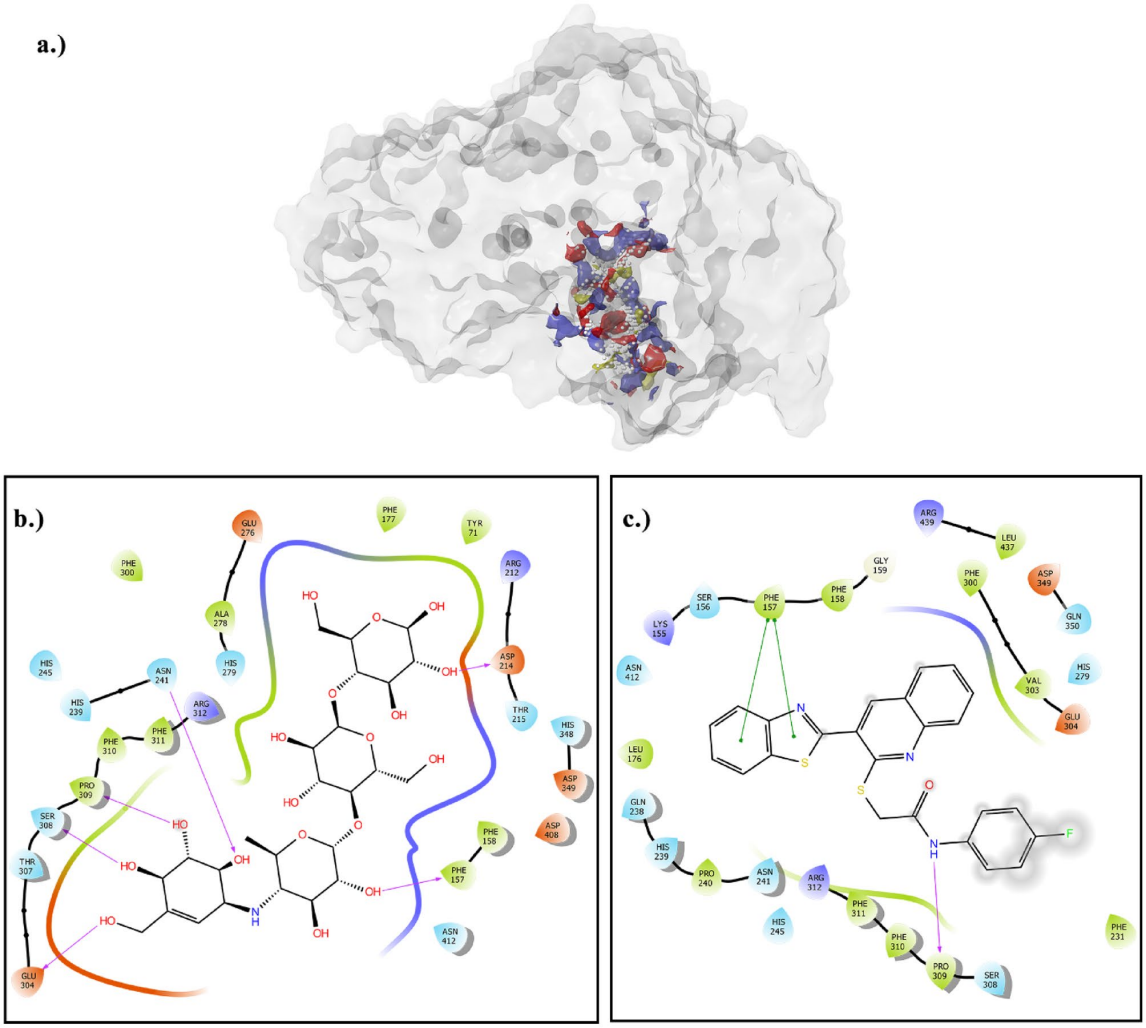
(**a**) Predicted active site of the enzyme based on the following properties: H-bond acceptor (red surface), H-bond donor (blue surface), hydrophobic sites (yellow surface). (**b**) Interaction of acarbose as the standard inhibitor with the active site pocket of *S. cerevisiae* α-glucosidase enzyme. (**c**) Interactions of compound 8h with the active site pocket of *S. cerevisiae* α-glucosidase enzyme.

To identify potential active sites of the modelled enzyme, a docking study was conducted on the protein model. The site mapping tool was utilized to identify five potential active sites, based on the site map score and overall surface area of the active sites^30^. Based on the scores and surface areas, the most probable active site was chosen as the grid box area for further docking studies. The active site selected for further studies was demonstrated in Fig. 7, and was found to contain a plausible surface area of hydrogen bond acceptor/donor and hydrophobic sites.

The predicted active site was utilized for the docking study with compound **8h**, which is the most biologically active compound that has been synthesized. The interactions between the compound **8h** and the active site pocket residues of the enzyme were observed, as shown in Fig. 5b. These interactions included a hydrogen bond between the amine hydrogen of the amide group and PRO 309, dual pi-pi stacking interactions between the benzothiazole system and PHE 157 residue, and several hydrophobic interactions with PRO 240, PHE 158, PHE 231, PHE 300, VAL 303, PHE 310, PHE 311, and LEU 176 residues^31^.

To obtain a comprehensive understanding of the docking conformation of compound 8h, a comparison has been made with acarbose, a standard inhibitor of the enzyme. In order to conduct this comparison, molecular docking performed with acarbose on the modeled enzyme, as depicted in Fig. 5. The primary interactions between acarbose and the active site pocket of the enzyme consist of hydrogen bond interactions with GLU 304, SER 308, ASN 241, PHE 157, and ASP 214. Additionally, numerous hydrophobic interactions have been observed between acarbose and PHE 300, PHE 310, PHE 311, TYR 71, and PHE 177. By analyzing the residues involved in the interaction of both molecules, it can be determined that compound **8h** exhibits a binding conformation that is quite similar to acarbose, as it interacts with the active site pocket of the enzyme.

### Molecular dynamics simulation

**Figure 8:**
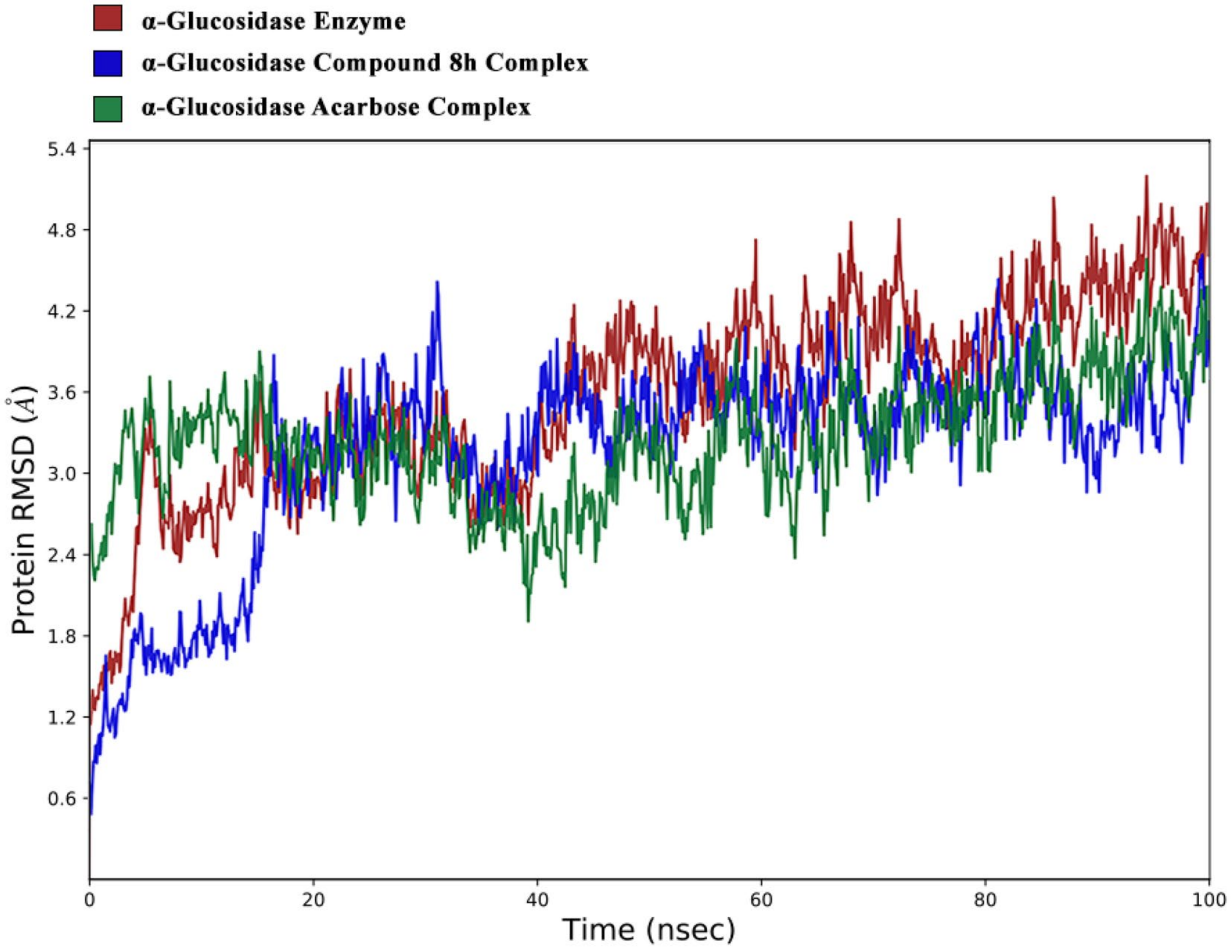
the RMSD values of the enzyme-compound **8h** complex and enzyme acarbose complex over the 100 ns simulation period.

The stability of the enzyme-inhibitor complex and enzyme were compared by evaluating the backbone root mean square deviation (RMSD) over 1000 trajectories during a 100 ns MD simulation (Fig. 8). The RMSD value of the α-glucosidase Enzyme stabilized after 5 ns, reaching an average value of 3 Å and remaining relatively constant with fewer fluctuations until 40 ns, after which the RMSD value increased significantly and continued to rise until the end of the simulation, with an average RMSD value of 4.5 Å^32,33^.

As shown in Fig. 8, the RMSD plot of the α-glucosidase and α-glucosidase-compound **8h** complex revealed that the complex stabilized after 20 ns, reaching an average value of 3 Å and maintaining the same level with an amplitude of 1.5 Å until the end of the simulation^34^. The overall RMSD values of the systems exhibited a significant difference, which can be attributed to the stabilizing effect of compound **8h** as an inhibitor on the enzyme. The RMSD values for the α-glucosidase-Acarbose complex reveal that the complex reached stability post 5 ns and maintained this stability until the 30 ns of simulation at 3 A. Following this, it diminished to 2 A until the 50 ns point in the simulation. From there, a subtle upward trend was noted until the end of the simulation. It's worth noting that when comparing the overall RMSD of the acarbose complex to the compound 8h complex, no substantial differences were identified.

**Figure 9:**
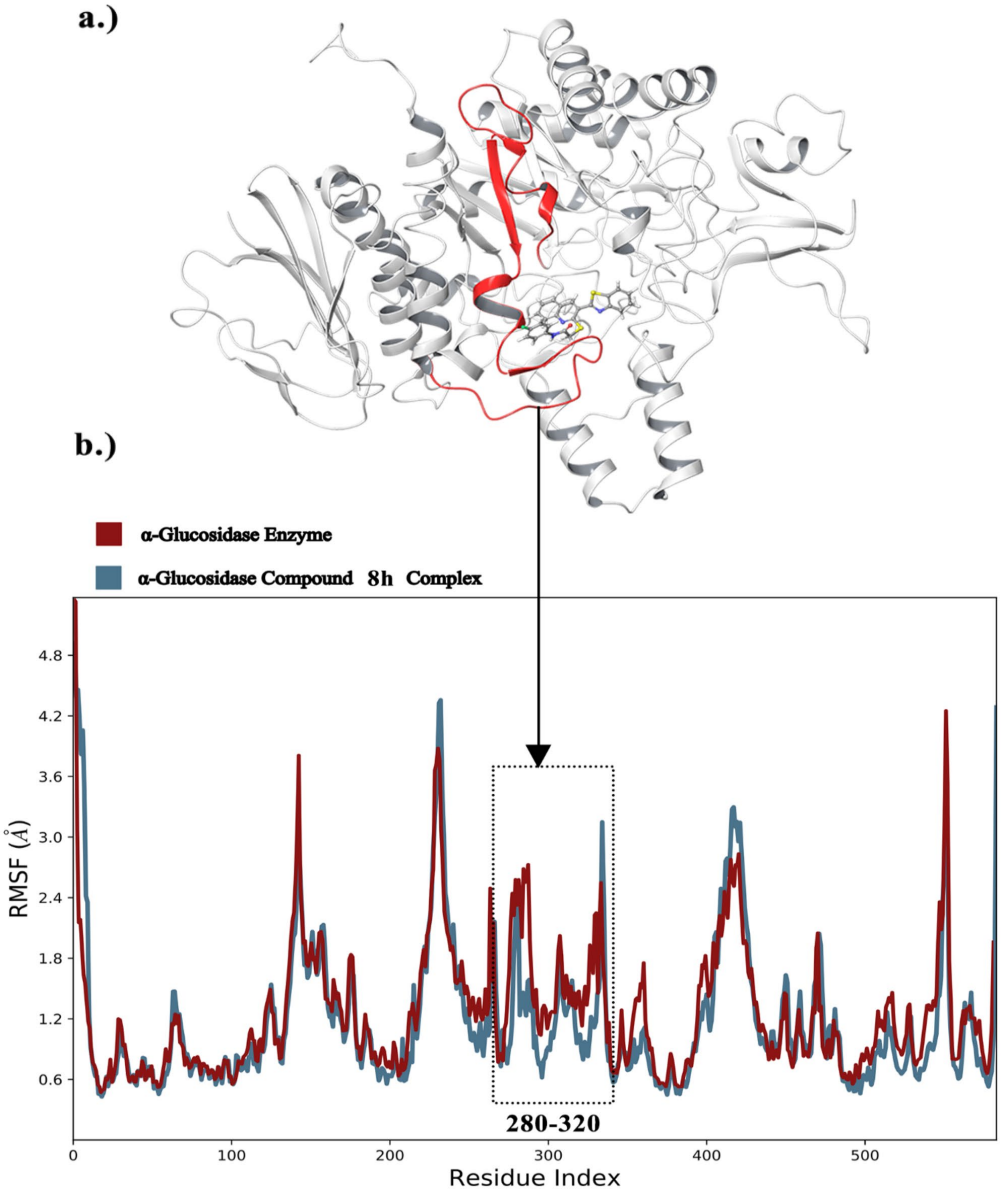
(**a**) The βαβ motif residues 280–320, which are located near the active site, are highlighted in red. (**b**) The RMSF values of the Enzyme and Enzyme-compound **8h** complex over the resides Index, the corresponding sequences in tertiary structure showed by red color.

The mechanism of ligand interaction with the enzyme can be elucidated through analysis of the root mean square fluctuations (RMSF) of Cα atoms from both systems. Decreases in residue movement were observed upon ligand binding to α-glucosidase, attributable to non-bonding interactions between the ligand and enzyme^35^. Figure 9 illustrates the most significant difference in fluctuations between the systems occurred in the amino acid range of 280–320, which corresponds to the βαβ motif nearby the active site (indicated in red).

**Figure 10:**
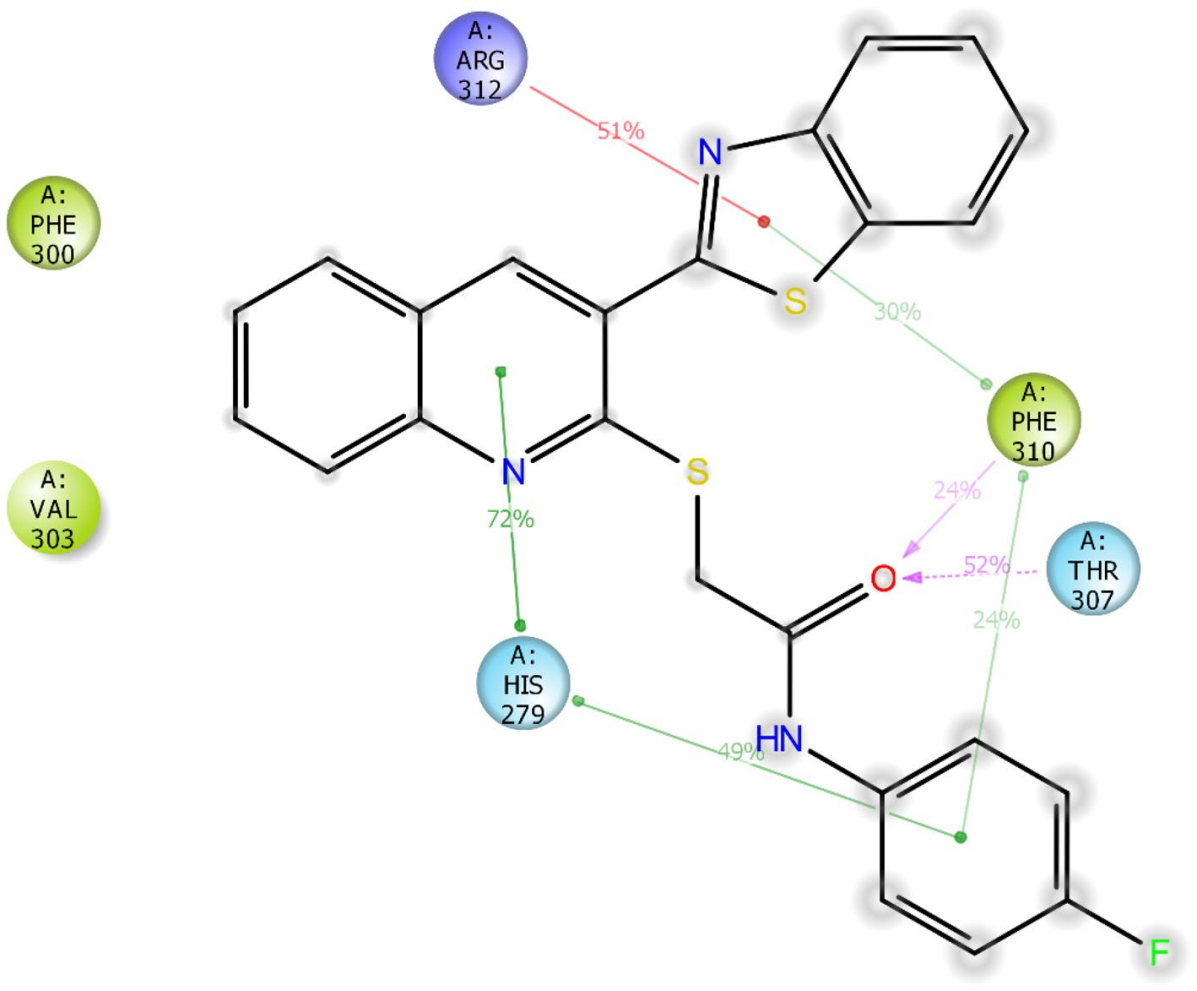
Schematic view of the detailed ligand atom interactions that occur with the active site residues for more than 30.0% of the simulation time during the 100ns simulation.

Figure 10 illustrates the interactions between compound **8h** and the enzyme's active site pocket, which were observed to occur for over 20% of the simulation duration. The interactions included hydrogen bonding between the amide group and residues PHE 310 and THR 307, a π-cation interaction between the benzothiazole group and ARG 312, as well as two dual π–π stack interactions: one between the quinoline and phenyl rings and HIS 279, and the other between the benzothiazole and phenyl rings with PHE 310. Additionally, hydrophobic interactions were observed with PHE 300 and VAL 303^36^.

### ADME‑Toxicity profiles and physicochemical properties

As part of preclinical drug development studies, the physicochemical properties and pharmacokinetic profile of the most potent α-glucosidase inhibitors (**8h**, **8n** and **8o**) were executed from the SwissADME website and Osiris DataWarrior. As illustrated in Table 2, the data obtained for the compounds are within the limits of Lipinski’s drug-likeness rule and have no violations. Accordingly, P-gp inhibition, log P and bioavailability score have favorable values and also there is no mutagenicity and tumorigenicity predicted for these compounds. Therefore, all information for are within the accepted range for drug-like molecules.

**Table 2:** The drug-likeness and ADMET parameters of the most potent inhibitors against α-glucosidase calculated by SwissADME^a^ and Osiris DataWarrior^b^.

Compound	IC50 (µM)	LogP^a^	Lipinski druglikeness^a^	p-gp substrate^a^	Bioavailability Score^a^	GI absorption^a^	Water solubility^a^	Mutagenic^b^	Tumorigenic^b^
**8h**	38.2 ± 0.3	3.35	Yes 0 violation	Yes	0.55	Low	Poorly soluble	None	None
**8n**	65.9 ± 1.0	3.83	Yes 0 violation	No	0.55	Low	Poorly soluble	None	None
**8o**	47.2 ± 0.6	2.36	Yes 0 violation	No	0.55	Low	Moderately soluble	None	None
**Acarbose**	750.0 ± 2.0	1.43	No 3 violations: MW > 500, HBA > 10, HBD > 5	Yes	0.17	Low	Highly soluble	None	None

## Conclusion

In conclusion, we have developed a new series of quinolone-benzothiazole hybrid including twenty derivatives. All synthesized compounds **8a–t** displayed promising *α*-glucosidase inhibitory activity in comparison to the standard drug acarbose. Three derivatives; **8h**, **8n** and **8o**, were represented significantly higher inhibitory potency than the others as they possess at least one electron-withdrawing group (F, NO_2_). Among the synthesized compounds, compound **8h** with fluorophenyl moiety on *para*-position, was found to be the most potent one with an IC_50_ value of 38.2 ± 0.3 μM. A kinetic study revealed that compound **8h** acts through a non-competitive inhibition mechanism with a *K*_*i*_ value of 38.2 μM. Moreover, a docking study and molecular dynamics simulation were conducted on the new protein which was generated via homology modeling. Results showed that the most active compounds **8h** interacted with important amino acids in the active site of the enzyme.

## Experimental

### Enzyme inhibition studies

The mode of inhibition of the most active compound **8h**, with the lowest IC_50_, was investigated against the α-glucosidase with different concentrations of *p*-nitrophenyl-*α*-d-glucopyranoside (1–16 mM) as substrate in the absence and presence of sample **8h** at different concentrations (0, 9.5, 19.1, and 38.2 µM). A Lineweaver–Burk plot was generated to recognize the type of inhibition and the Michaelis–Menten constant (*K*_m_) value was defined from the plot between the reciprocal of the substrate concentration (1/[S]) and reciprocal of enzyme rate (1/V) over different concentrations of inhibitor.

### Homology modeling

Homology modeling was performed using the amino acid sequence FASTA file of the saccharomyces cerevisiae α-glucosidase enzyme (EC. 3. 2. 1. 20), which was downloaded from uniprot.org (1) using the uniprot code P38158. The saccharomyces cerevisiae isomaltase enzyme (PDB ID: 3A47) was chosen as the template enzyme based on previous reports (3). The homology modeling was conducted using Maestro Prime (4).

### Molecular docking

Molecular modeling investigations were carried out using the Maestro Molecular Modeling platform (version 10.5) by Schrödinger, LLC (2).

For molecular docking, the modeled protein from the previous stage was prepared using the Protein Preparation Wizard (5), and missing sidechains and loops were filled using the Prime tool (4). H-bonds were assigned by PROPKA at PH: 7.4. The 2D structure of the ligand was drawn in ChemDraw (ver. 16) and exported as an SDF file for use in the next step. The ligand was prepared using the OPLS_2005 forcefield and EPIK (7) at a target PH of 7.0 ± 2, using the LigPrep module (6). The SiteMap tool (8) was utilized to identify potential binding sites of the enzyme–substrate complex, with the SiteMap report including 5 potential binding sites with at least 15 site points per site and a more restrictive definition of hydrophobicity. A grid box was generated for each binding site using the reported sites as entries, with a box size of 25 Å. Compound **8h** was docked onto the binding sites using Glide (9) with standard precision and flexible ligand sampling, with 20 poses reported per ligand.

### Molecular dynamic

Molecular dynamics simulations were performed using Desmond from the Schrodinger Maestro interface (10), based on the results of the previous docking stage. An orthorhombic cell was defined and filled with TIP3P model water molecules, and adequate Na ions were added to the system to neutralize the overall charge of the complex. The simulation time was 100 ns, and the NPT ensemble was applied with a constant number of atoms, constant pressure (1.01325 bar), and constant temperature (300 K), using the 1.0-ps interval Nose–Hoover chain method as the default thermostat and the 2.0-ps interval Martyna-Tobias-Klein as the default barostat. The results of the molecular dynamics simulation were analyzed using the Maestro graphical interface (11).

### General procedure for the preparation of 2-((3-(benzo[d]thiazol-2-yl)quinolin-2-yl)thio)-N-arylacetamide (8a–t)

A mixture of 3-(benzo[d]thiazol-2-yl)quinoline-2-thiol (1 mmol) **7**, 2-chloro-*N*-substituted acetamide derivatives **5a–t** (1.2 mmol) in dry acetone (10 mL) and anhydrous K_2_CO_3_ (1 mmol) was stirred at room temperature for 4 h, filtered and the solid product formed was crystallized in ethanol to give final products **8a–t**.

#### 2-((3-(benzo[d]thiazol-2-yl)quinolin-2-yl)thio)-N-phenylacetamide (8a)

Cream solid;Yield:71%; mp: 193–195 °C; IR (KBr, v_max_) 3213 (NH), 3020 (CH Aromatic), 2965 (CH Aliphatic), 1671 (C=O) Cm^−1^; ^1^H NMR (500 MHz, DMSO-d6) δ 10.41 (s, 1H, NH_Amid_), 8.85 (s, 1H, H_4_), 8.24 (d, *J* = *8.00 Hz,* 1H, H_Ar_), 8.17 (d, *J = 8.20 Hz*, 1H, H_Ar_), 8.11 (d, *J* = *8.20 Hz*, 1H, H_Ar_), 7.91 (d, *J* = *8.40 Hz*, 2H, H_Ar_), 7.80 (t, *J* = *7.70 Hz*, 1H, H_Ar_), 7.61 (d, *J* = *7.80 Hz*, 2H, H_Ar_), 8.57–8.53 (m, 1H, H_Ar_), 7.30 (t, *J* = *7.70 Hz*, 2H, H_Ar_), 7.03 (t, *J* = *7.40 Hz*, 1H, H_Ar_), 4.21 (s, 2H, CH_2_) ppm. ^13^C NMR (125 MHz, DMSO-d6): δ 167.55, 164.32, 156.93, 153.53, 147.44, 139.75, 138.74, 135.34, 132.28, 129.24, 127.55, 127.37, 126.89, 126.61, 125.61, 125.18, 123.70, 122.82, 119.54, 36.64, ppm; ESI–MS (C_24_H_17_N_3_OS_2_): calculated m/z 427.08 [M + H]^+^, observed m/z 427.24 [M + H]^+^; Anal. Calcd: C_24_H_17_N_3_OS_2_; C, 67.42; H, 4.01; N, 9.83; Found; C, 67.59; H, 4.38; N, 9.98.

#### 2-((3-(benzo[d]thiazol-2-yl)quinolin-2-yl)thio)-N-(o-tolyl)acetamide (8b)

Cream solid;Yield:68%; mp: 211–213 °C; IR (KBr, v_max_) 3221 (NH), 3025 (CH Aromatic), 2960 (CH Aliphatic), 1679 (C=O) Cm^−1^; ^1^H NMR (500 MHz, DMSO-d6) δ 9.67 (s, 1H, NH_Amid_), 8.87 (s, 1H, H_4_), 8.24 (d, *J* = *8.20 Hz,* 1H, H_Ar_), 8.17 (d, *J* = *8.20 Hz*, 1H, H_Ar_), 8.13 (d, *J* = *8.20 Hz*, 1H, H_Ar_), 7.98 (d, *J* = *8.40 Hz*, 1H, H_Ar_), 7.84 (t, *J* = *7.70 Hz*, 1H, H_Ar_), 7.61 (d, *J* = *8.10 Hz*, 2H, H_Ar_), 7.55 (t, *J* = *7.70 Hz*, 1H, H_Ar_), 7.38 (d, *J* = *8.10 Hz*, 1H, H_Ar_), 7.17 (d, *J* = *7.50 Hz*, 1H, H_Ar_), 7.11 (t, *J* = *7.40 Hz*, 1H, H_Ar_), 7.06 (d, *J* = *7.60 Hz*, 1H, H_Ar_), 4.26 (s, 2H, CH_2_), 2.14 (s, 3H, CH_3_) ppm. ^13^C NMR (125 MHz, DMSO-d6): δ 167.44, 164.32, 156.92, 153.53, 147.50, 138.82, 136.85, 135.38, 132.28, 132.05, 130.72, 129.29, 127.65, 127.36, 126.92, 126.60, 126.39, 125.77, 125.60, 125.24, 123.69, 122.83, 35.90, ppm ; ESI–MS (C_25_H_19_N_3_OS_2_): calculated m/z 441.10 [M + H]^+^, observed m/z 441.21 [M + H]^+^; Anal. Calcd: C_25_H_19_N_3_OS_2_; C, 68.00; H, 4.34; N, 9.52; Found; C, 68.21; H, 4.58; N, 9.76.

#### 2-((3-(benzo[d]thiazol-2-yl)quinolin-2-yl)thio)-N-(p-tolyl)acetamide (8c)

Cream solid;Yield:68%; mp: 217–219 °C; IR (KBr, v_max_) 3234 (NH), 3040 (CH Aromatic), 2975 (CH Aliphatic), 1664 (C=O) Cm^−1^; ^1^H NMR (500 MHz, DMSO-d6) δ 10.32 (s, 1H, NH_Amid_), 8.84 (s, 1H, H_4_), 8.23 (d, *J* = *7.90 Hz,* 1H, H_Ar_), 8.16 (d, *J* = *8.40 Hz*, 1H, H_Ar_), 8.10 (d, *J* = *8.10 Hz*, 1H, H_Ar_), 7.91 (d, *J* = *8.40 Hz*, 1H, H_Ar_), 7.80 (t, *J* = *7.80 Hz*, 1H, H_Ar_), 7.62 (d, *J* = *7.60 Hz*, 1H, H_Ar_), 7.55 (d, *J* = *7.70 Hz*, 1H, H_Ar_), 7.51 (d, *J* = *7.70 Hz*, 2H, H_Ar_), 7.47 (d, *J* = *7.90 Hz*, 1H, H_Ar_), 7.09 (d, *J* = *7.90 Hz*, 1H, H_Ar_), 4.19 (s, 2H, CH_2_), 2.23 (s, 3H, CH_3_) ppm. ^13^C NMR (125 MHz, DMSO-d6): δ 167.27, 164.31, 156.95, 153.53, 147.45, 138.72, 137.24, 135.34, 132.59, 132.28, 129.60, 129.25, 127.57, 127.36, 126.89, 126.60, 125.63, 125. 18., 123.70, 122.82, 119.67, 119.55, 36.60, 20.89, ppm; ESI–MS (C_25_H_19_N_3_OS_2_): calculated m/z 441.10 [M + H]^+^, observed m/z 441.12 [M + H]^+^; Anal. Calcd : C_25_H_19_N_3_OS_2_; C, 68.00; H, 4.34; N, 9.52; Found; C, 68.16; H, 4.49; N, 9.68.

#### 2-((3-(benzo[d]thiazol-2-yl)quinolin-2-yl)thio)-N-(4-ethylphenyl)acetamide (8d)

Cream solid;Yield:81%; mp: 195–197 °C; IR (KBr, v_max_) 3401 (NH), 3065(CH Aromatic), 2950(CH Aliphatic), 1670 (C=O) Cm^−1^; ^1^H NMR (500 MHz, DMSO-d6) δ 10.33 (s, 1H, NH_Amid_), 8.85 (s, 1H, H_4_), 8.24 (d, *J* = *8.00 Hz,* 1H, H_Ar_), 8.17 (d, *J* = *8.20 Hz*, 1H, H_Ar_), 8.11 (d, *J* = *8.10 Hz*, 1H, H_Ar_), 7.92 (d, *J* = *8.40 Hz*, 1H, H_Ar_), 7.81 (t, *J* = *7.80 Hz*, 1H, H_Ar_), 7.62 (t, *J* = *7.80 Hz*, 1H, H_Ar_), 7.60–7.54 (m, 2H, H_Ar_), 7.52 (d, *J* = *8.50 Hz*, 2H, H_Ar_), 7.12 (t, *J* = *8.00 Hz*, 2H, H_Ar_), 4.19 (s, 2H, CH_2_), 3.63 (d, *J* = *7.60 Hz*, 2H, CH_2Ethyl_), 1.13 (t, *J* = *7.60 Hz*, 3H, CH_3Ethyl_), ppm. ^13^C NMR (125 MHz, DMSO-d6): δ 167.34, 164.31, 156.90, 153.51, 147.44, 139.15, 138.70, 137.36, 135.32, 132.29, 129.23, 128.41, 127.57, 127.38, 126.91, 126.63, 125.61, 125.16, 123.69, 122.79, 119.80, 119.68, 36.54, 28.03, 16.13, ppm; ESI–MS (C_26_H_21_N_3_OS_2_): calculated m/z 455.11 [M + H]^+^, observed m/z 455.19[M + H]^+^; Anal. Calcd: C_26_H_21_N_3_OS_2_; C, 68.54; H, 4.65; N, 9.22; Found; C, 68.69; H, 4.84; N, 9.41.

#### 2-((3-(benzo[d]thiazol-2-yl)quinolin-2-yl)thio)-N-(2,3-dimethylphenyl)acetamide (8e)

Cream solid; Yield: 66%; mp: 210–212 °C; IR (KBr, v_max_) 3227 (NH), 3020 (CH Aromatic), 2965 (CH Aliphatic), 1678 (C=O) Cm^−1^; ^1^H NMR (500 MHz, DMSO-d6) δ 9.73 (s, 1H, NH_Amid_), 8.87 (s, 1H, H_4_), 8.24 (d, *J* = *8.10 Hz,* 1H, H_Ar_), 8.17 (d, *J* = *8.20 Hz*, 1H, H_Ar_), 8.13 (d, *J* = *8.30 Hz*, 1H, H_Ar_), 7.99 (d, *J* = *8.40 Hz*, 1H, H_Ar_), 7.84 (t, *J* = *7.80 Hz*, 1H, H_Ar_), 8.61 (d, *J* = *7.90 Hz*, 2H, H_Ar_), 7.56 (d, *J* = *7.60 Hz*, 1H, H_Ar_), 7.12 (d, *J* = *7.60 Hz*, 1H, H_Ar_), 7.05–6.95 (m, 2H, H_Ar_), 4.25 (s, 2H, CH_2_), 2.20 (s, 3H, CH_3_), 2.01 (s, 3H, CH_3_), ppm. ^13^C NMR (125 MHz, DMSO-d6): δ 167.45, 164.33, 156.97, 153.53, 147.52, 138.80, 137.38, 136.64, 135.39, 132.27, 131.59, 129.29, 127.68, 127.36, 126.91, 126.60, 125.77, 125.61, 125.24, 123.78, 123.70, 122.83, 35.83, 20.56, 14.46, ppm; ESI–MS (C_26_H_21_N_3_OS_2_): calculated m/z 455.11 [M + H]^+^, observed m/z 455.24 [M + H]^+^; Anal. Calcd: C_26_H_21_N_3_OS_2_; C, 68.54; H, 4.65; N, 9.22; Found; C, 68.69; H, 4.83; N, 9.51.

#### 2-((3-(benzo[d]thiazol-2-yl)quinolin-2-yl)thio)-N-(2,6-dimethylphenyl)acetamide (8f)

Cream solid; Yield: 68%; mp: 215–217 °C; IR (KBr, v_max_) 3235 (NH), 3030 (CH Aromatic), 2970 (CH Aliphatic), 1684 (C=O) Cm^−1^; ^1^H NMR (500 MHz, DMSO-d6) δ 9.56 (s, 1H, NH_Amid_), 8.87 (s, 1H, H_4_), 8.25 (d, *J* = *8.00 Hz,* 1H, H_Ar_), 8.18–8.11(m, 2H, H_Ar_), 8.02 (d, *J* = *8.50 Hz*, 1H, H_Ar_), 7.85 (t, *J* = *7.60 Hz*, 1H, H_Ar_), 7.62 (d, *J* = *7.40 Hz*, 2H, H_Ar_), 7.55 (t, *J* = *7.60 Hz*, 1H, H_Ar_), 7.02–6.96 (m, 3H, H_Ar_), 4.28 (s, 2H, CH_2_), 2.06 (s, 6H, 2xCH_3_) ppm. ^13^C NMR (125 MHz, DMSO-d6): δ 168.77, 160.08, 155.46, 150.11, 144.22, 138.82, 136.14, 135.73, 135.56, 135.41, 133.67, 132.21, 131.24, 130.18, 129.27, 128.18, 128.03, 127.73, 127.38, 126.93, 126.88, 126.60, 123.68, 122.83, 121.62, 37.82, 18.48, ppm; ESI–MS (C_26_H_21_N_3_OS_2_): calculated m/z 455.11 [M + H]^+^, observed m/z 455.19[M + H]^+^; Anal. Calcd: C_26_H_21_N_3_OS_2_; C, 68.54; H, 4.65; N, 9.22; Found; C, 68.72; H, 4.87; N, 9.45.

#### 2-((3-(benzo[d]thiazol-2-yl)quinolin-2-yl)thio)-N-(2-fluorophenyl)acetamide (8g)

Brown solid; Yield: 65%; mp: 201–203 °C; IR (KBr, v_max_) 3343(NH), 3045 (C-H Aromatic), 2970 (CH Aliphatic), 1654 (C=O) Cm^−1^; ^1^H NMR (500 MHz, DMSO-d6) δ 10.47 (s, 1H, NH_Amid_), 8.86 (s, 1H, H_4_), 8.25 (d, *J* = *8.10 Hz,* 1H, H_Ar_), 8.17 (d, *J* = *8.20 Hz*, 1H, H_Ar_), 8.12 (d, *J* = *8.10 Hz*, 1H, H_Ar_), 7.90 (d, *J* = *8.40 Hz*,1H, H_Ar_), 7.80 (t, *J* = *7.70 Hz*, 1H, H_Ar_), 7.63 (d, *J* = *6.70 Hz*, 2H, H_Ar_), 7.59–7.50 (m, 2H, H_Ar_), 7.14 (t, *J* = *8.80 Hz*, 3H, H_Ar_), 4.20 (s, 2H, CH_2_) ppm. ^13^C NMR (125 MHz, DMSO-d6): δ 167.48, 164.32, 156.91, 147.44, 138.76, 135.33, 132.31, 129.28, 127.54, 127.37, 126.90, 126.62, 125.61, 125.19, 123.70, 122.84, 121.30, 121.23, 115.88, 115.71, 36.55 ppm; ESI–MS (C_24_H_16_FN_3_OS_2_): calculated m/z 445.07 [M + H]^+^, observed m/z 445.20 [M + H]^+^; Anal. Calcd: C_24_H_16_FN_3_OS_2_; C, 64.70; H, 3.62; N, 9.43; Found; C, 64.86; H, 3.71; N, 9.68.

#### 2-((3-(benzo[d]thiazol-2-yl)quinolin-2-yl)thio)-N-(4-fluorophenyl)acetamide (8h)

Brown solid; Yield: 68%; mp: 219–221 °C; IR (KBr, v_max_) 3354(NH), 3030 (C–H Aromatic), 2980 (CH Aliphatic), 1659 (C=O) Cm^−1^; ^1^H NMR (500 MHz, DMSO-d6) δ 9.91 (s, 1H, NH_Amid_), 8.88 (s, 1H, H_4_), 8.23 (d, *J* = *8.10 Hz,* 1H, H_Ar_), 8.16 (d, *J* = *8.00 Hz*, 1H, H_Ar_), 8.12 (d, *J* = *8.10 Hz*, 1H, H_Ar_), 7.95 (d, *J* = *8.40 Hz* ,1H, H_Ar_), 7.83 (d, *J* = *8.40 Hz*, 2H, H_Ar_), 7.65–7.56 (m, 4H, H_Ar_), 7.54 (t, *J* = *7.90 Hz*, 1H, H_Ar_), 7.37 (d, *J* = *8.40 Hz*, 1H, H_Ar_), 4.29 (s, 2H, CH_2_) ppm. ^13^C NMR (125 MHz, DMSO-d6): δ 168.32, 164.29, 156.56, 153.52, 147.44, 138.93, 135.31, 134.64, 132.32, 129.30, 129.26, 128.07, 127.67, 127.39, 127.03, 126.65, 126.38, 125.65, 125.28, 123.70, 122.83, 35.87, ppm; ESI–MS (C_24_H_16_FN_3_OS_2_): calculated m/z 445.07 [M + H]^+^, observed m/z 445.18 [M + H]^+^; Anal. Calcd: C_24_H_16_FN_3_OS_2_; C, 64.70; H, 3.62; N, 9.43; Found; C, 64.91; H, 3.76; N, 9.59.

#### 2-((3-(benzo[d]thiazol-2-yl)quinolin-2-yl)thio)-N-(2-chlorophenyl)acetamide (8i)

Brown solid; Yield: 71%; mp: 221–223 °C; IR (KBr, v_max_) 3327(NH), 3015 (C–H Aromatic), 2940 (CH Aliphatic), 1657 (C=O) Cm^−1^; ^1^H NMR (500 MHz,DMSO-d6) δ 10.33 (s, 1H, NH_Amid_), 8.88 (s, 1H, H_4_), 8.24 (d, *J* = *8.00 Hz,* 1H, H_Ar_), 8.18 (d, *J* = *8.10 Hz*, 1H, H_Ar_), 8.15–8.09 (m, 2H, H_Ar_), 8..03 (d, *J* = *8.40 Hz*, 1H, H_Ar_), 7.91 (d, *J* = *8.50 Hz* ,1H, H_Ar_), 7.82 (t, *J* = *7.90 Hz*, 1H, H_Ar_), 7.75 (d, *J* = *8.30 Hz*, 1H, H_Ar_), 7.62 (t, *J* = *8.10 Hz*, 1H, H_Ar_), 7.55–7.46 (m, 1H, H_Ar_), 7.38 (t, *J* = *8.00 Hz*, 2H, H_Ar_), 4.40 (s, 2H, CH_2_) ppm. ^13^C NMR (125 MHz, DMSO-d6): δ 166.98, 164.31, 156.97, 155.66, 153.53, 147.46, 138.73, 135.35, 132.89, 132.29, 129.26, 127.58, 127.37, 126.89, 126.61, 125.65, 125.18, 123.70, 122.83, 121.10, 114.35, 36.50, ppm; ESI–MS (C_24_H_16_ClN_3_OS_2_): calculated m/z 461.04 [M + H]^+^, observed m/z 461.23 [M + H]^+^; Anal. Calcd: C_24_H_16_ClN_3_OS_2_; C, 62.40; H, 3.49; N, 9.10; Found; C, 62.49; H, 3.69; N, 9.32.

#### 2-((3-(benzo[d]thiazol-2-yl)quinolin-2-yl)thio)-N-(3-chlorophenyl)acetamide (8j)

Brown solid; Yield: 65%; mp: 226–228 °C; IR (KBr, v_max_) 3336(NH), 3030 (C–H Aromatic), 2965 (CH Aliphatic), 1651(C=O) Cm^−1^; ^1^H NMR (500 MHz, DMSO-d6) δ 10.62 (s, 1H, NH_Amid_), 8.87(s, 1H, H_4_), 8.25 (d, *J* = *8.00 Hz,* 1H, H_Ar_), 8.17 (d, *J* = *8.10 Hz*, 1H, H_Ar_), 8.12 (d, *J* = *8.10 Hz*, 1H, H_Ar_), 7.86 (d, *J* = *8.40 Hz*, 1H, H_Ar_), 7.83–7.78 (m, 2H, H_Ar_), 7.63 (t, *J* = *7.70 Hz*, 1H, H_Ar_), 7.59–7.54 (m, 2H, H_Ar_), 7.50 (d, *J* = *8.20 Hz*, 1H, H_Ar_), 7.34 (t, *J* = *8.30 Hz*, 1H, H_Ar_), 7.09 (d, *J* = *7.90 Hz*, 1H, H_Ar_), 4.20 (s, 2H, CH_2_) ppm. ^13^C NMR (125 MHz, DMSO-d6): δ 168.07, 164.33, 156.83, 147.41, 142.73, 141.20, 138.77, 135.32, 133.57, 132.33, 130.98, 129.30, 127.39, 126.94, 126.64, 125.57, 125.20, 123.71, 123.38, 122.85, 118.95, 117.87, 36.65, ppm; ESI–MS (C_24_H_16_ClN_3_OS_2_): calculated m/z 461.04 [M + H]^+^, observed m/z 461.11 [M + H]^+^; Anal. Calcd: C_24_H_16_ClN_3_OS_2_; C, 62.40; H, 3.49; N, 9.10; Found; C, 62.53; H, 3.72; N, 9.38.

#### 2-((3-(benzo[d]thiazol-2-yl)quinolin-2-yl)thio)-N-(4-chlorophenyl)acetamide (8k)

Brown solid; Yield: 73%; mp: 2230–228 °C; IR (KBr, v_max_) 3328(NH), 3040 (C–H Aromatic), 2950 (CH Aliphatic), 1668 (C=O) Cm^−1^; ^1^H NMR (500 MHz, DMSO-d6) δ 10.56 (s, 1H, NH_Amid_), 8.85 (s, 1H, H_4_), 8.23 (d, *J* = *8.00 Hz,* 1H, H_Ar_), 8.16 (d, *J* = *8.20 Hz*, 1H, H_Ar_), 8.11 (d, *J* = *8.20 Hz*, 1H, H_Ar_), 7.87 (d, *J* = *8.40 Hz* ,1H, H_Ar_), 7.79 (t, *J* = *8.00 Hz*, 1H, H_Ar_), 6.67 (d, *J* = *8.40 Hz*, 2H, H_Ar_), 7.62 (t, *J* = *8.20 Hz*, 1H, H_Ar_), 7.58–7.50 (m, 2H, H_Ar_), 7.35 (d, *J* = *8.50 Hz*, 2H, H_Ar_), 4.20 (s, 2H, CH_2_) ppm. ^13^C NMR (125 MHz, DMSO-d6): δ 167.80, 164.32, 156.86, 153.53, 147.42, 138.72, 135.32, 132.31, 129.27, 129.15, 129.10, 128.93, 127.50, 127.37, 126.91, 126.62, 125.57, 125.18, 123.70, 123.39, 122.99, 122.82, 121.18, 121.05, 120.96, 36.52, ppm ; ESI–MS (C_24_H_16_ClN_3_OS_2_): calculated m/z 461.04 [M + H]^+^, observed m/z 461.11 [M + H]^+^; Anal. Calcd: C_24_H_16_ClN_3_OS_2_; C, 62.40; H, 3.49; N, 9.10; Found; C, 62.61; H, 3.64; N, 9.27.

#### 2-((3-(benzo[d]thiazol-2-yl)quinolin-2-yl)thio)-N-(4-bromophenyl)acetamide (8l)

Brown solid; Yield: 70%; mp: 187–189 °C; IR (KBr, v_max_) 3320(NH), 3030 (C–H Aromatic), 2960 (CH Aliphatic), 1650 (C=O) Cm^−1^; ^1^H NMR (500 MHz, DMSO-d6) δ 10.56 (s, 1H, NH_Amid_), 8.85 (s, 1H, H_4_), 8.23 (d, *J* = *8.00 Hz,* 1H, H_Ar_), 8.16 (d, *J* = *8.10 Hz*, 1H, H_Ar_), 8.10 (d, *J* = *8.20 Hz*, 1H, H_Ar_), 7.87 (d, *J* = *8.40 Hz*, 1H, H_Ar_), 7.80 (t, *J* = *7.60 Hz*, 1H, H_Ar_), 7.65–7.58 (m, 3H, H_Ar_), 7.48 (d, *J* = *8.50 Hz*, 2H, H_Ar_), 4.20 (s, 2H, CH_2_) ppm. ^13^C NMR (125 MHz, DMSO-d6): δ 167.81, 159.50, 156.87, 153.53, 143.57, 137.40, 135.32, 133.55, 132.07, 131.15, 129.29, 127.74, 127.39, 126.93, 126.64, 125.57, 125.19, 124.28, 123.71, 122.85, 121.92, 121.75, 121.43, 38.76, ppm; ESI–MS (C_24_H_16_BrN_3_OS_2_): calculated m/z 504.99 [M + H]^+^, observed m/z 505.08 [M + H]^+^; Anal. Calcd: C_24_H_16_BrN_3_OS_2_; C, 56.92; H, 3.18; N, 8.30; Found; C, 57.12; H, 3.34; N, 8.49.

#### 2-((3-(benzo[d]thiazol-2-yl)quinolin-2-yl)thio)-N-(4-nitrophenyl)acetamide (8m)

Cream solid; Yield: 71%;mp: 209–211 °C; IR (KBr, v_max_) 3375 (NH), 3045 (CH Aromatic), 2960(CH Aliphatic), 1665 (C=O) Cm^−1^; ^1^H NMR (500 MHz,DMSO-d6) δ 11.07 (s, 1H, NH_Amid_), 8.87 (s, 1H, H_4_), 8.25 (d, *J* = *8.10 Hz,* 1H, H_Ar_), 8.23 (d, *J* = *8.80 Hz*, 2H, H_Ar_), 8.11 (d, *J* = *8.20 Hz*, 1H, H_Ar_), 7.89 (d, *J* = *8.80 Hz*, 2H, H_Ar_), 7.80 (t, *J* = *8.60 Hz*, 1H, H_Ar_), 7.76 (d, *J* = *8.10 Hz*, 1H, H_Ar_), 7.62 (t, *J* = *7.60 Hz*, 1H, H_Ar_), 7.58–7.53 (m, 2H, H_Ar_), 4.25 (s, 2H, CH_2_), ppm. ^13^C NMR (125 MHz, DMSO-d6): δ 168.84, 164.34, 156.73, 153.53, 147.36, 145.92, 142.61, 138.78, 135.27, 132.35, 129.28, 127.39, 126.94, 126.65, 125.56, 125.49, 125.19, 123.69, 122.83, 119.13, 36.84, ppm; ESI–MS (C_24_H_16_N_4_O_3_S_2_): calculated m/z 479.02 [M + H]^+^, observed m/z 479.23 [M + H]^+^; Anal. Calcd: C_24_H_16_N_4_O_3_S_2_; C, 52.60; H, 2.73; N, 14.60; Found; C, 52.78; H, 2.90; N, 14.82.

#### 2-((3-(benzo[d]thiazol-2-yl)quinolin-2-yl)thio)-N-(4-methyl-2-nitrophenyl)acetamide (8n)

Cream solid; Yield: 71%; mp: 221–223 °C; IR (KBr, v_max_) 3364 (NH), 3035 (CH Aromatic), 2950 (CH Aliphatic), 1661 (C=O) Cm^−1^; ^1^H NMR (500 MHz, DMSO-d6) δ 9.94 (s, 1H, NH_Amid_), 8.88 (s, 1H, H_4_), 8.24 (d, *J* = *8.10 Hz,* 1H, H_Ar_), 8.17 (d, *J* = *8.10 Hz*, 1H, H_Ar_), 8.14 (d, *J* = *8.20 Hz*, 1H, H_Ar_), 7.11–7.09 (m, 1H, H_Ar_), 8.03 (d, *J* = *8.20 Hz*, 1H, H_Ar_), 7.92 (t, *J* = *8.00 Hz*, 1H, H_Ar_), 7.89 (d, *J* = *8.10 Hz*, 1H, H_Ar_), 7.81 (t, *J* = *7.80 Hz*, 1H, H_Ar_), 7.63–7.57 (m, 2H, H_Ar_), 7.56–7.53 (m, 1H, H_Ar_), 4.25 (s, 2H, CH_2_), 2.34 (s, 3H, CH_2_) ppm. ^13^C NMR (125 MHz, DMSO-d6): δ 168.40, 164.32, 156.73, 153.53, 147.42, 143.58, 143.47, 138.89, 135.32, 132.37, 129.32, 127.50, 127.39, 126.99, 126.64, 125.92, 125.85, 125.65, 125.27, 123.69, 123.45, 122.84, 122.29, 122.22, 36.21, 18.27, ppm; ESI–MS (C_25_H_18_N_4_O_3_S_2_): calculated m/z 486.08 [M + H]^+^, observed m/z 486.19 [M + H]^+^; Anal. Calcd: C_25_H_18_N_4_O_3_S_2_; C, 61.71; H, 3.73; N, 11.51; Found; C, 61.87; H, 3.95; N, 11.70.

#### 2-((3-(benzo[d]thiazol-2-yl)quinolin-2-yl)thio)-N-(5-nitrothiazol-2-yl)acetamide (8o)

Cream solid; Yield: 68%; mp: 199–201 °C; IR (KBr, v_max_) 3355 (NH), 3070(CH Aromatic), 2965(CH Aliphatic), 1670 (C=O) Cm^−1^; ^1^H NMR (500 MHz,DMSO-d6) δ 10.93 (s, 1H, NH_Amid_), 8.81 (s, 1H, H_Ar_), 8.49 (s, 1H, H_4_), 8.23 (d, *J* = *8.00 Hz*, 1H, H_Ar_), 8.18 (d, *J* = *8.20 Hz*, 1H, H_Ar_), 8.09 (d, *J* = *8.10 Hz*, 1H, H_Ar_), 7.85 (d, *J* = *8.50 Hz*, 1H, H_Ar_), 7.79 (t, *J* = *7.90 Hz*, 1H, H_Ar_), 7.61 (t, *J* = *7.70 Hz*, 1H, H_Ar_), 7.55 (d, *J* = *8.00 Hz*, 1H, H_Ar_), 4.32 (s, 2H, CH_2_), ppm. ^13^C NMR (125 MHz, DMSO-d6): δ 164.31, 157.00, 153.47, 147.41, 145.39, 138.64, 135.37, 132.26, 129.21, 127.43, 127.34, 126.82, 126.58, 125.71, 125.11, 123.69, 122.78, 37.62, ppm; ESI–MS (C_21_H_13_N_5_O_3_S_3_): calculated m/z 479.02 [M + H]^+^, observed m/z 479.23 [M + H]^+^; Anal. Calcd: C_26_H_21_N_3_OS_2_ C, 52.60; H, 2.73; N, 14.60; Found; C, 52.78; H, 2.90; N, 14.82.

#### 2-((3-(benzo[d]thiazol-2-yl)quinolin-2-yl)thio)-N-(naphthalen-1-yl)acetamide (8p)

Cream solid; Yield: 62%; mp: 198–200 °C; IR (KBr, v_max_) 3224 (NH), 3025 (CH Aromatic), 2980 (CH Aliphatic), 1673 (C=O) Cm^−1^; ^1^H NMR (500 MHz, DMSO-d6) δ 10.33 (s, 1H, NH_Amid_), 8.88 (s, 1H, H_4_), 8.24 (d, *J* = *8.00 Hz,* 1H, H_Ar_), 8.18 (d, *J* = *8.40 Hz*, 1H, H_Ar_), 8.16–8.08 (m, 2H, H_Ar_), 8.03 (d, *J* = *8.30 Hz*, 1H, H_Ar_), 7.91 (t, *J* = *8.50 Hz*, 1H, H_Ar_), 7.82 (t, *J* = *8.00 Hz*, 1H, H_Ar_), 7.75 (d, *J* = *8.20 Hz*, 1H, H_Ar_), 7.62 (t, *J* = *8.00 Hz*, 2H, H_Ar_), 7.56–7.45 (m, 3H, H_Ar_), 7.38 (t, *J* = *8.00 Hz*, 2H, H_Ar_), 4.41 (s, 2H, CH_2_), 2.06 (s, 6H, 2xCH_3_) ppm. ^13^C NMR (125 MHz, DMSO-d6): δ 168.26, 159.21, 153.55, 149.33, 144.49, 134.15, 132.28, 130.26, 129.32, 128.54, 128.42, 127.70, 127.38, 126.95, 126.62, 126.47, 126.11, 126.04, 125.88, 123.71, 123.37, 123.01, 122.84, 122.22, 122.01, 121.86, 119.67, 38.87, ppm; ESI–MS (C_28_H_19_N_3_OS_2_): calculated m/z 477.10 [M + H]^+^, observed m/z 477.32 [M + H]^+^; Anal. Calcd: C_28_H_19_N_3_OS_2_; C, 70.41; H, 4.01; N, 8.80; Found; C, 70.53; H, 4.31; N, 8.97.

#### 2-((3-(benzo[d]thiazol-2-yl)quinolin-2-yl)thio)-N-benzylacetamide (8q)

Brown solid; Yield: 65%; mp: 180–183° C; IR (KBr, v_max_) 3320(NH), 3040 (C–H Aromatic), 2980(CH Aliphatic), 1680 (C=O) Cm^−1^; ^1^H NMR (500 MHz, DMSO-d6) δ 8.84 (s, 1H, H_4_), 8.70 (t, *J* = *6.30 Hz,* 1H, NH_Amid_), 8.23 (d, *J* = *8.20 Hz,* 1H, H_Ar_), 8.16 (d, *J* = *8.20 Hz*, 1H, H_Ar_), 8.11 (d, *J* = *8.10 Hz*, 1H, H_Ar_), 7.88 (d, *J* = *8.50 Hz*, 1H, H_Ar_), 7.82 (t, *J* = *7.80 Hz*, 1H, H_Ar_), 7.61 (d, *J* = *7.50 Hz*, 2H, H_Ar_), 7.54 (t, *J* = *7.80 Hz*, 1H, H_Ar_), 7.23–6.12 (m, 5H, H_Ar_), 4.31 (d, *J* = *6.10 Hz*, 2H, CH_2Benzyl_), 4.10 (s, 2H, CH_2_), ppm. ^13^C NMR (125 MHz, DMSO-d6): δ 168.43, 164.33, 156.84, 153.53, 147.49, 139.80, 138.74, 135.39, 132.16, 129.20, 128.56, 127.82, 127.50, 127.35, 127.08, 126.87, 126.58, 125.78, 125.21, 123.68, 122.82, 42.94, 35.28, ppm; ESI–MS (C_25_H_19_N_3_OS_2_): calculated m/z 441.10 [M + H]^+^, observed m/z 441.18 [M + H]^+^; Anal. Calcd: C_25_H_19_N_3_OS_2_; C, 68.00; H, 4.34; N, 9.52; Found; C, 68.17; H, 4.58; N, 9.69.

#### 2-((3-(benzo[d]thiazol-2-yl)quinolin-2-yl)thio)-N-(4-methylbenzyl)acetamide (8r)

Brown solid; Yield: 74%; mp: 182–184 °C; IR (KBr, v_max_) 3326(NH), 3045 (C–H Aromatic), 2990(CH Aliphatic), 1679 (C=O) Cm^−1^; ^1^H NMR (500 MHz, DMSO-d6) δ 8.84 (s, 1H, H_4_), 8.64 (t, *J* = *6.00 Hz,* 1H, NH_Amid_), 8.24 (d, *J* = *8.00 Hz,* 1H, H_Ar_), 8.16 (d, *J* = *8.10 Hz*, 1H, H_Ar_), 8.12 (d, *J* = *8.20 Hz*, 1H, H_Ar_), 7.85 (d, *J* = *8.10 Hz*, 1H, H_Ar_), 7.80 (t, *J* = *7.90 Hz*, 1H, H_Ar_), 7.61 (d, *J* = *7.50 Hz*, 2H, H_Ar_), 7.55 (t, *J* = *7.80 Hz*, 1H, H_Ar_), 7.08 (d, *J* = *8.10 Hz*, 2H, H_Ar_), 6.97 (d, *J* = *8.10 Hz*, 2H, H_Ar_), 4.25 (d, *J* = *5.90 Hz*, 1H, CH_2Benzyl_), 4.07 (s, 2H, CH_2_), 2.22 (s, 23H, CH_3_), ppm. ^13^C NMR (125 MHz, DMSO-d6): δ 168.40, 164.32, 156.79, 153.51, 147.47, 138.69, 136.70, 136.11, 135.38, 132.10, 129.44, 129.30, 129.27, 129.21, 129.15, 129.12, 128.94, 127.81, 127.69, 127.54, 127.35, 126.85, 126.58, 125.75, 125.17, 123.67, 122.78, 42.73, 35.30, 21.11, ppm; ESI–MS (C_26_H_21_N_3_OS_2_): calculated m/z 455.11 [M + H]^+^, observed m/z 455.36 [M + H]^+^; Anal. Calcd: C_26_H_21_N_3_OS_2_; C, 68.54; H, 4.65; N, 9.22; Found; C, 68.71; H, 4.91; N, 9.39.

#### 2-((3-(benzo[d]thiazol-2-yl)quinolin-2-yl)thio)-N-(4-fluorobenzyl)acetamide (8s)

Brown solid; Yield: 74%; mp: 213–215 °C; IR (KBr, v_max_) 3337(NH), 3030 (C–H Aromatic), 2980(CH Aliphatic), 1662 (C=O) Cm^−1^; ^1^H NMR (500 MHz, DMSO-d6) δ 8.84 (s, 1H, H_4_), 8.70 (t, *J* = *6.40 Hz,* 1H, NH_Amid_), 8.23 (d, *J* = *8.00 Hz,* 1H, H_Ar_), 8.16 (d, *J* = *8.10 Hz*, 1H, H_Ar_), 8.11 (d, *J* = *8.10 Hz*, 1H, H_Ar_), 7.85–7.77 (m, 2H, H_Ar_), 7.60 (d, *J* = *7.70 Hz*, 2H, H_Ar_), 7.54 (t, *J* = *7.70 Hz*, 1H, H_Ar_), 7.23 (*d*, *J* = *6.90 Hz*, 2H, H_Ar_), 6.96 (t, *J* = *8.60 Hz*, 2H, H_Ar_), 4.28 (d, *J* = *6.00 Hz*, 2H, CH_2Benzyl_), 4.08 (s, 2H, CH_2_), ppm. ^13^C NMR (125 MHz, DMSO-d6): δ 168.52, 164.33, 160.53, 156.77, 153.51, 147.44, 138.72, 136.01, 135.37, 132.13, 129.53, 129.47, 129.19, 127.75, 127.36, 126.88, 126.60, 125.76, 125.17, 123.67, 122.81, 115.30, 115.13, 42.25, 35.27, ppm; ESI–MS (C_25_H_18_FN_3_OS_2_): calculated m/z 459.09 [M + H]^+^, observed m/z 459.21 [M + H]^+^; Anal. Calcd: C_25_H_18_FN_3_OS_2_; C, 65.34; H, 3.95; N, 9.14; Found; C, 65.51; H, 4.09; N, 9.33.

#### 2-((3-(benzo[d]thiazol-2-yl)quinolin-2-yl)thio)-N-phenethylacetamide (8t)

Cream solid; Yield: 66%; mp: 225–227 °C; IR (KBr, v_max_) 3243 (NH), 3050 (CH Aromatic), 2975 (CH Aliphatic), 1679 (C=O) Cm^−1^; ^1^H NMR (500 MHz, DMSO-d6) δ 8.84 (s, 1H, H_4_), 8.30–8.22 (m, 2H, NH_Amid_, H_Ar_), 8.16 (d, *J* = *8.10 Hz,* 1H, H_Ar_), 8.12 (d, *J* = *8.10 Hz*, 1H, H_Ar_), 8.90 (d, *J* = *8.50 Hz*, 1H, H_Ar_), 7.83 (t, *J* = *7.70 Hz*, 1H, H_Ar_), 7.61 (d, *J* = *7.90 Hz*, 2H, H_Ar_), 7.55 (t, *J* = *7.60 Hz*, 1H, H_Ar_), 7.16 (d, *J* = *6.90 Hz*, 2H, H_Ar_) 7.12 (d, *J* = *7.20 Hz*, 2H, H_Ar_), 3.99 (s, 2H, CH_2_), 3.32–3.25(m, 2H, CH_2_), 2.69 (t, *J* = *7.40 Hz*, 3H, CH_3Ethyl_), ppm. ^13^C NMR (125 MHz, DMSO-d6): δ 168.37, 164.28, 156.82, 153.52, 147.47, 139.79, 138.74, 135.40, 132.23, 129.21, 128.97, 128.67, 127.68, 127.34, 126.87, 126.58, 126.45, 125.75, 125.21, 123.69, 122.82, 41.09, 41.08, 35.51, ppm; ESI–MS (C_26_H_21_N_3_OS_2_): calculated m/z 455.11 [M + H]^+^, observed m/z 455.27[M + H]^+^; Anal. Calcd: C_26_H_21_N_3_OS_2_; C, 68.54; H, 4.65; N, 9.22; Found; C, 68.69; H, 4.90; N, 9.41.

### Supplementary Information

Supplementary Information.

## Data Availability

All data generated or analyzed during this study are included in this published article and its Supplementary Information files.
