# Supplementary material for: Synthesis, in vitro potency of inhibition, enzyme kinetics and in silico studies of quinoline-based α-glucosidase inhibitors
Source: Sci Rep. 2024 Jan 4;14:501. doi: 10.1038/s41598-023-50711-2 (PMC10766639; doi:10.1038/s41598-023-50711-2)
Supplement: Supplementary file 1 — Supplementary Information. [file 41598_2023_50711_MOESM1_ESM.pdf]

Supplementary material

Quinoline-based α-glucosidase inhibitors: synthesis, *in vitro* potency of inhibition, enzyme kinetics and *in silico* study

Minoo Khalili Ghomi [a], *Navid Dastyafteh* [a], *Mohammad Nazari Montazer* [a], *Milad Noori* [a], *Somayeh Mojtabavi* [b], *Mohammad Ali Faramarzi* [b], *Seyedeh Mahdieh Hashemi* [c,*], *Mohammad Mahdavi* [a,*]

[a] *Endocrinology and Metabolism Research Center, Endocrinology and Metabolism Clinical Sciences Institute, Tehran University of Medical Sciences, Tehran, Iran*

[b] *Department of Pharmaceutical Biotechnology, Faculty of Pharmacy and Biotechnology Research Center, Tehran University of Medical Sciences, Tehran, Iran*

[c] *Department of Medicinal Chemistry and Pharmaceutical Sciences Research Center, Faculty of Pharmacy, Mazandaran University of Medical Sciences, Sari, Iran*

Contents:

- ^{1}H NMR spectra of compounds **8a- 8t**.
- ^{13}C NMR spectra of compounds **8a- 8t**.

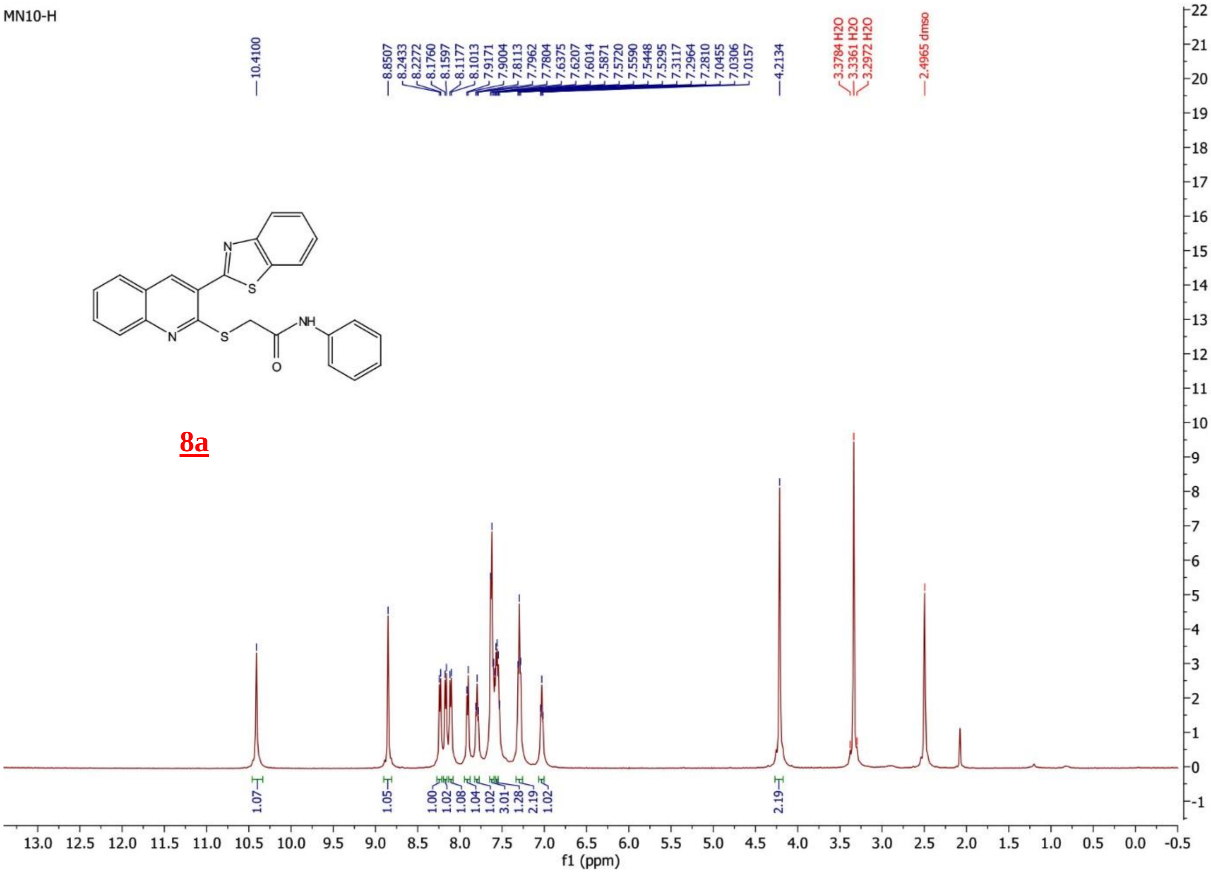
MN10-H
10.4100
8.8507
8.2433
8.2272
8.1760
8.1597
8.1177
8.1013
7.9171
7.9004
7.8113
7.7962
7.7804
7.6375
7.6207
7.6014
7.5871
7.5720
7.5590
7.5448
7.5295
7.3117
7.2964
7.2810
7.0455
7.0306
7.0157
4.2134
3.3784 H2O
3.3361 H2O
3.2972 H2O
2.4965 dmso
N
S
N
S
NH
O
1.07
1.05
1.00
1.02
1.08
1.04
1.02
3.01
1.28
2.19
1.02
2.19
f1 (ppm)

8a

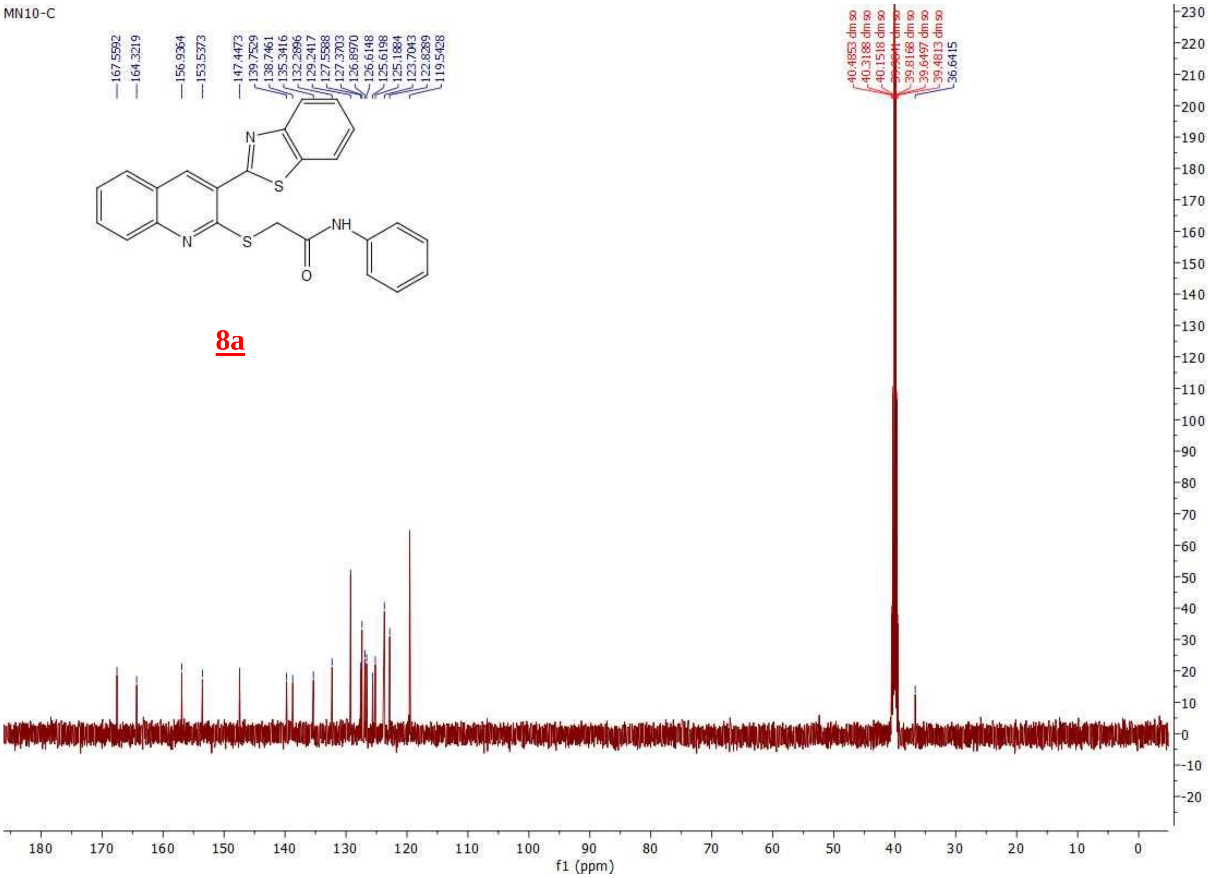
MN10-C
167.5592
164.3219
156.9364
153.5373
147.4473
139.7529
138.7461
135.3416
132.2896
129.2417
127.5588
127.3703
126.8970
126.6148
125.6198
125.1884
123.7043
122.8289
119.5428
40.4853 dmso
40.3188 dmso
40.1518 dmso
39.8168 dmso
39.6497 dmso
39.4813 dmso
36.6415
N
S
N
S
NH
O
8a
f1 (ppm)

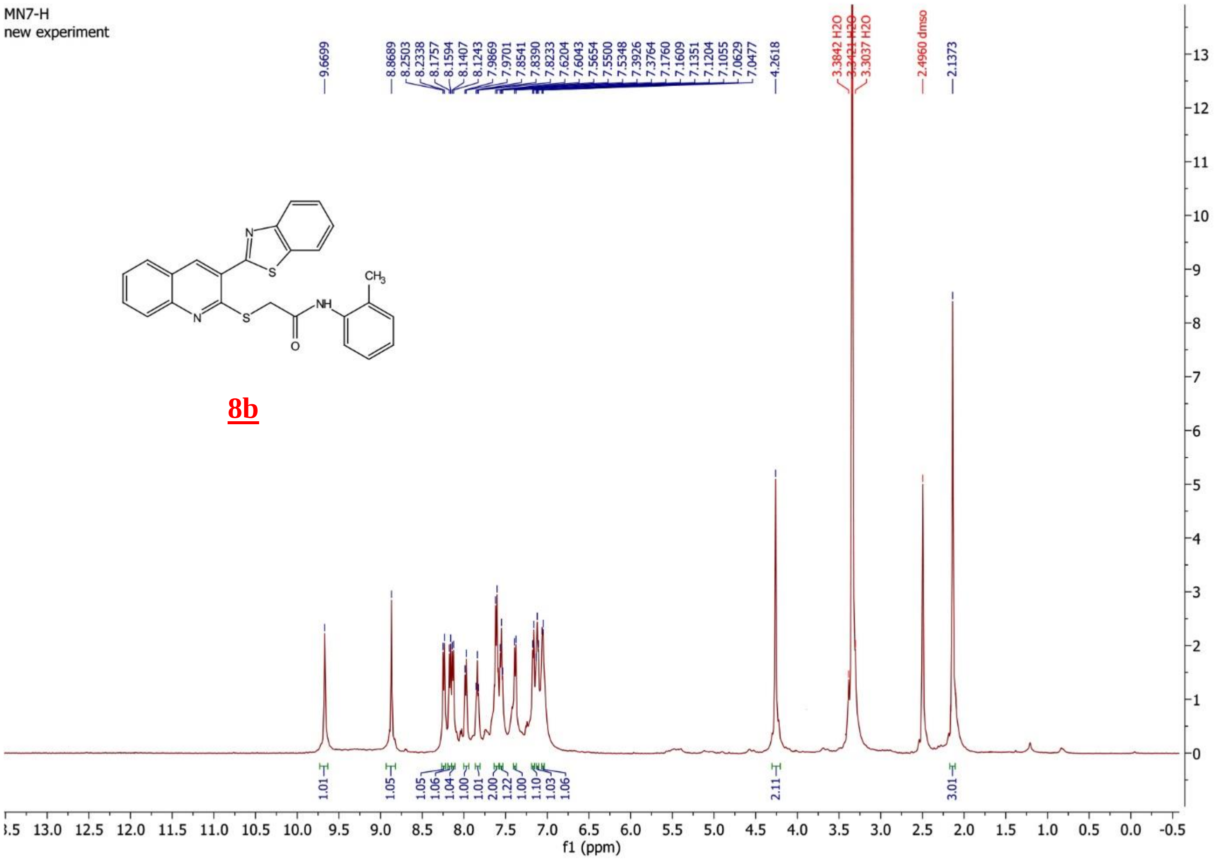
MN7-H
new experiment
9.6699
8.8689
8.2503
8.2338
8.1757
8.1594
8.1407
8.1243
7.9869
7.9701
7.8541
7.8390
7.8233
7.6204
7.6043
7.5654
7.5500
7.5348
7.3926
7.3764
7.1760
7.1609
7.1351
7.1204
7.1055
7.0629
7.0477
4.2618
3.3842 H2O
3.3421 H2O
3.3037 H2O
2.4960 dmso
2.1373
N
S
CH3
NH
O
1.01
1.05
1.05
1.06
1.04
1.00
1.01
2.00
1.22
1.00
1.10
1.03
1.06
2.11
3.01
f1 (ppm)

8b

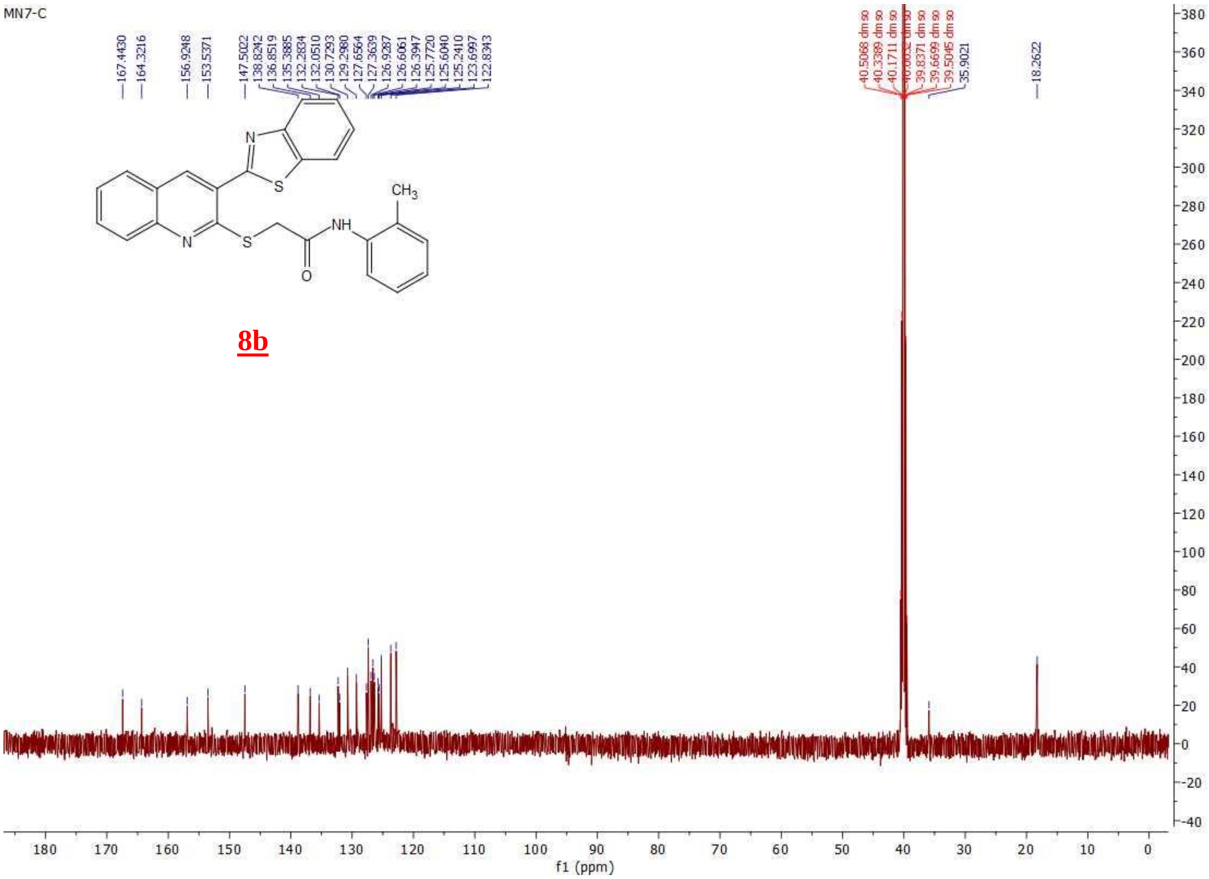
MN7-C
167.4430
164.3216
156.9248
153.5371
147.5022
138.8242
136.8519
135.3885
132.2834
132.0510
130.7293
129.2980
127.6564
127.3639
126.9287
126.6061
126.3947
125.7720
125.6040
125.2410
123.6997
122.8343
40.5068 dmso
40.3389 dmso
40.1711 dmso
39.8371 dmso
39.6699 dmso
39.5045 dmso
35.9021
18.2622
N
S
CH3
NH
O
f1 (ppm)

8b

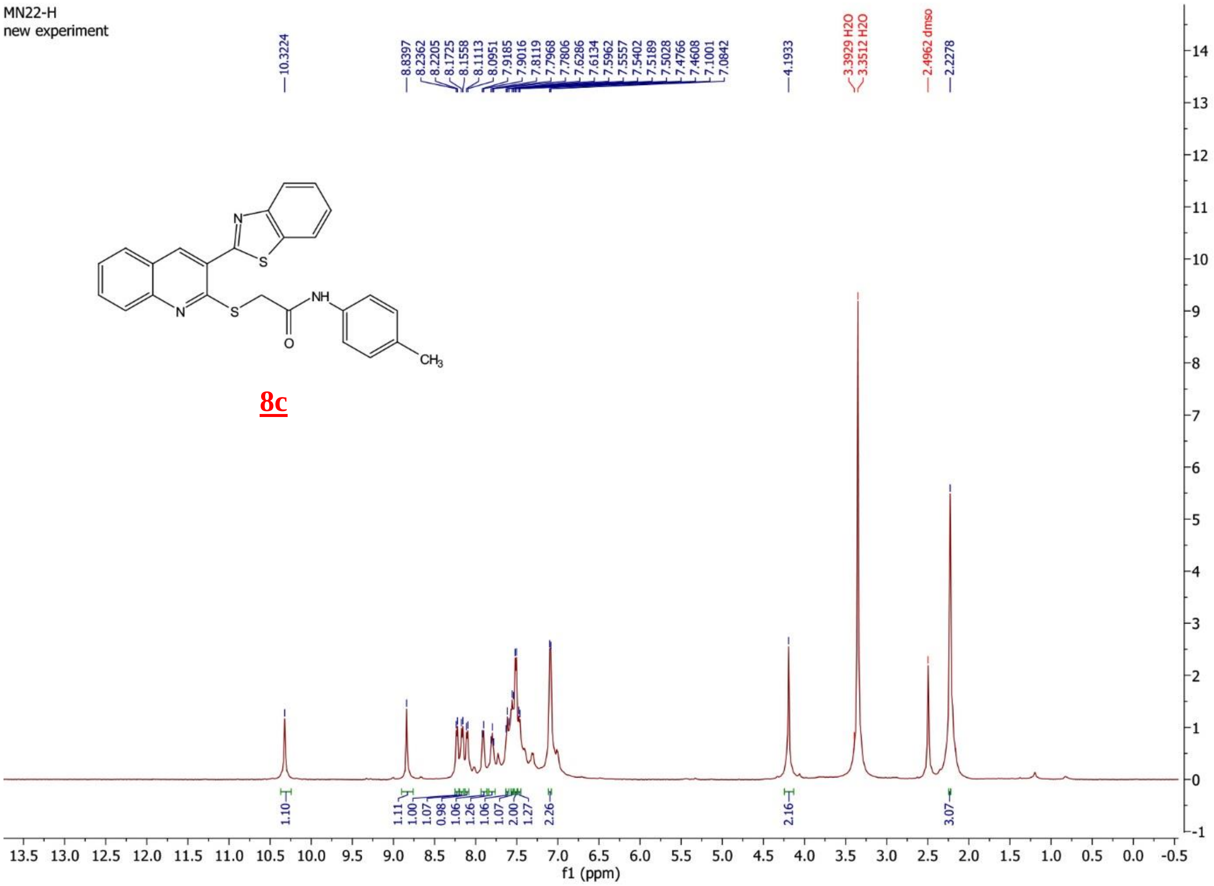
MN22-H
new experiment
10.3224
8.8397
8.2362
8.2205
8.1725
8.1558
8.1113
8.0951
7.9185
7.9016
7.8119
7.7968
7.7806
7.6286
7.6134
7.5962
7.5557
7.5402
7.5189
7.5028
7.4766
7.4608
7.1001
7.0842
4.1933
3.3929 H2O
3.3512 H2O
2.4962 dmso
2.2278
N
S
N
S
NH
O
CH3
1.10
1.11
1.00
1.07
0.98
1.06
1.26
1.06
1.07
2.00
1.27
2.26
2.16
3.07
f1 (ppm)

8c

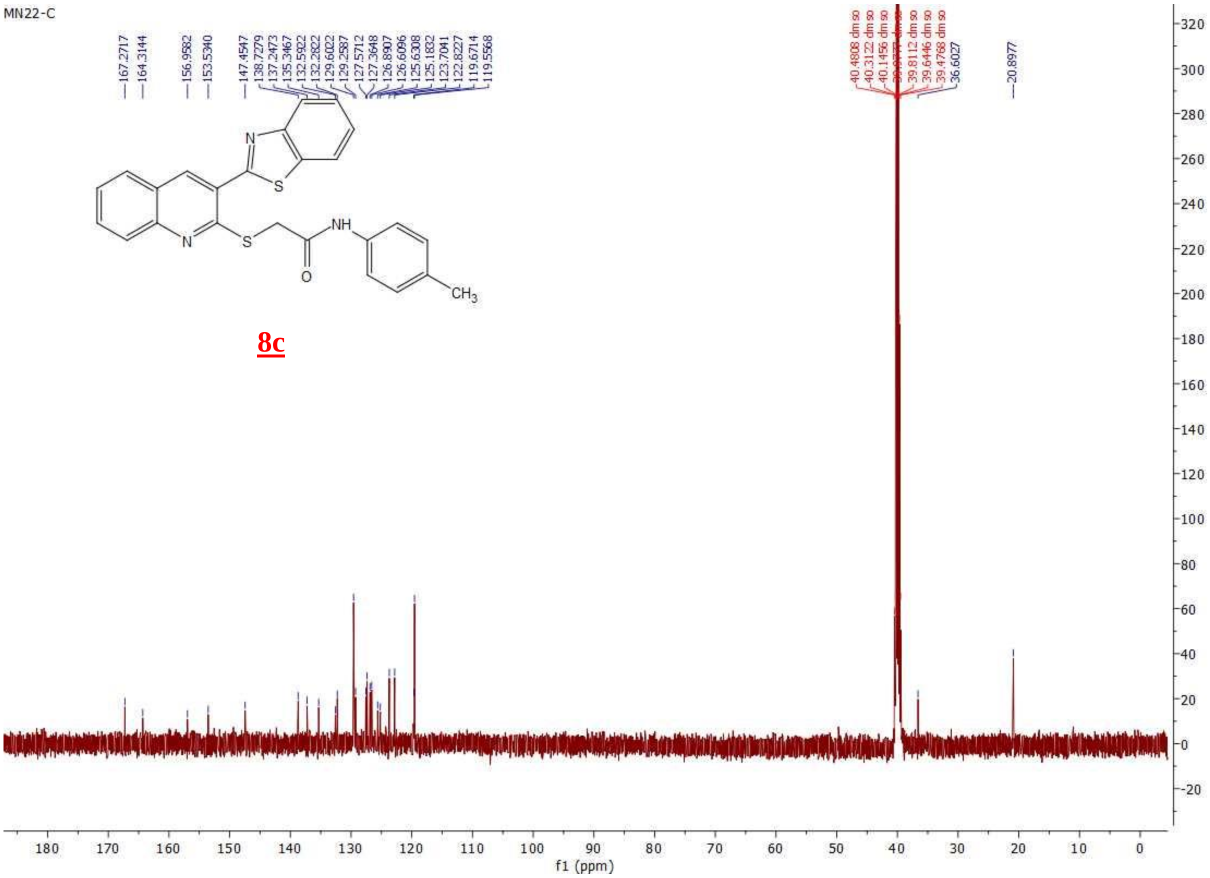
MN22-C
167.2717
164.3144
156.9582
153.5340
147.4547
138.7279
137.2473
135.3467
132.5922
132.2822
129.6022
129.2587
127.5712
127.3648
126.8907
126.6096
125.6308
125.1832
123.7041
122.8227
119.6714
119.5568
40.4808 dmso
40.3122 dmso
40.1456 dmso
39.8112 dmso
39.6446 dmso
39.4768 dmso
36.6027
20.8977
N
S
N
S
NH
O
CH3
8c
f1 (ppm)

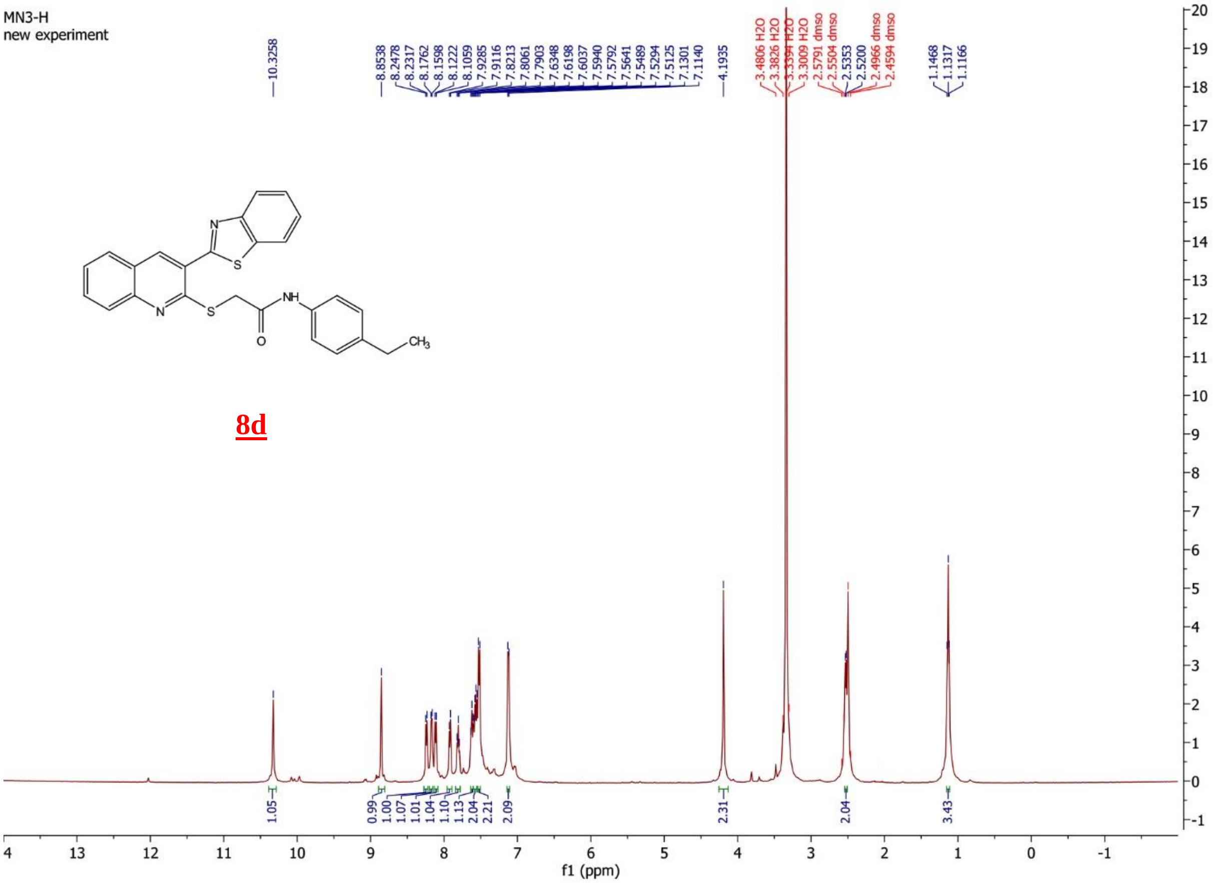
MN3-H
new experiment
10.3258
8.8538
8.2478
8.2317
8.1762
8.1598
8.1222
8.1059
7.9285
7.9116
7.8213
7.8061
7.7903
7.6348
7.6198
7.6037
7.5940
7.5792
7.5641
7.5489
7.5294
7.5125
7.1301
7.1140
4.1935
3.4806 H2O
3.3826 H2O
3.3394 H2O
3.3009 H2O
2.5791 dmso
2.5504 dmso
2.5353
2.5200
2.4966 dmso
2.4594 dmso
1.1468
1.1317
1.1166
N
S
N
S
NH
O
CH3
1.05
0.99
1.00
1.07
1.01
1.04
1.10
1.13
2.04
2.21
2.09
2.31
2.04
3.43
f1 (ppm)

8d

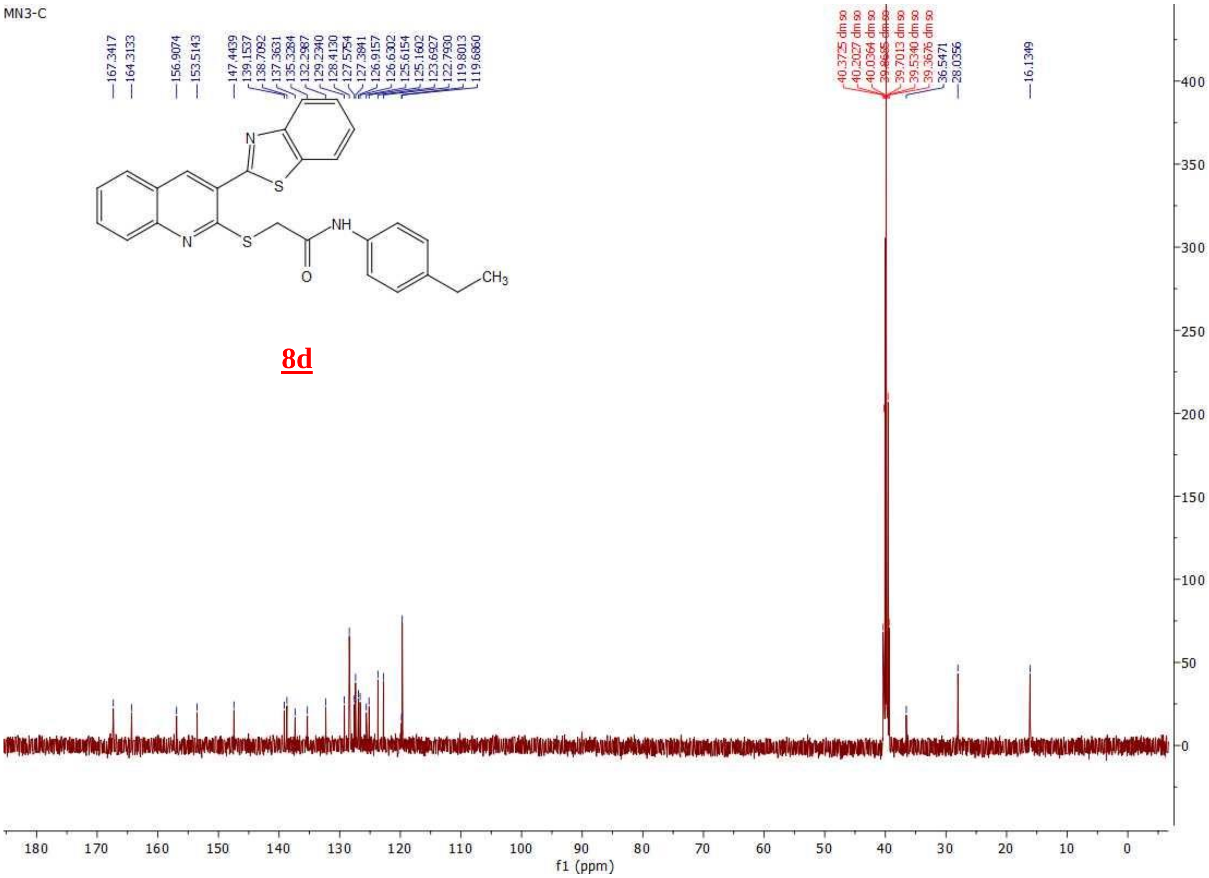
MN3-C
167.3417
164.3133
156.9074
153.5143
147.4439
139.1537
138.7092
137.3631
135.3284
132.2987
129.2340
128.4130
127.5754
127.3841
126.9157
126.6302
125.6154
125.1602
123.6927
122.7930
119.8013
119.6860
40.3725 dmso
40.2027 dmso
40.0364 dmso
39.8685 dmso
39.7013 dmso
39.5340 dmso
39.3676 dmso
36.5471
28.0356
16.1349
N
S
N
S
NH
O
CH3
f1 (ppm)

8d

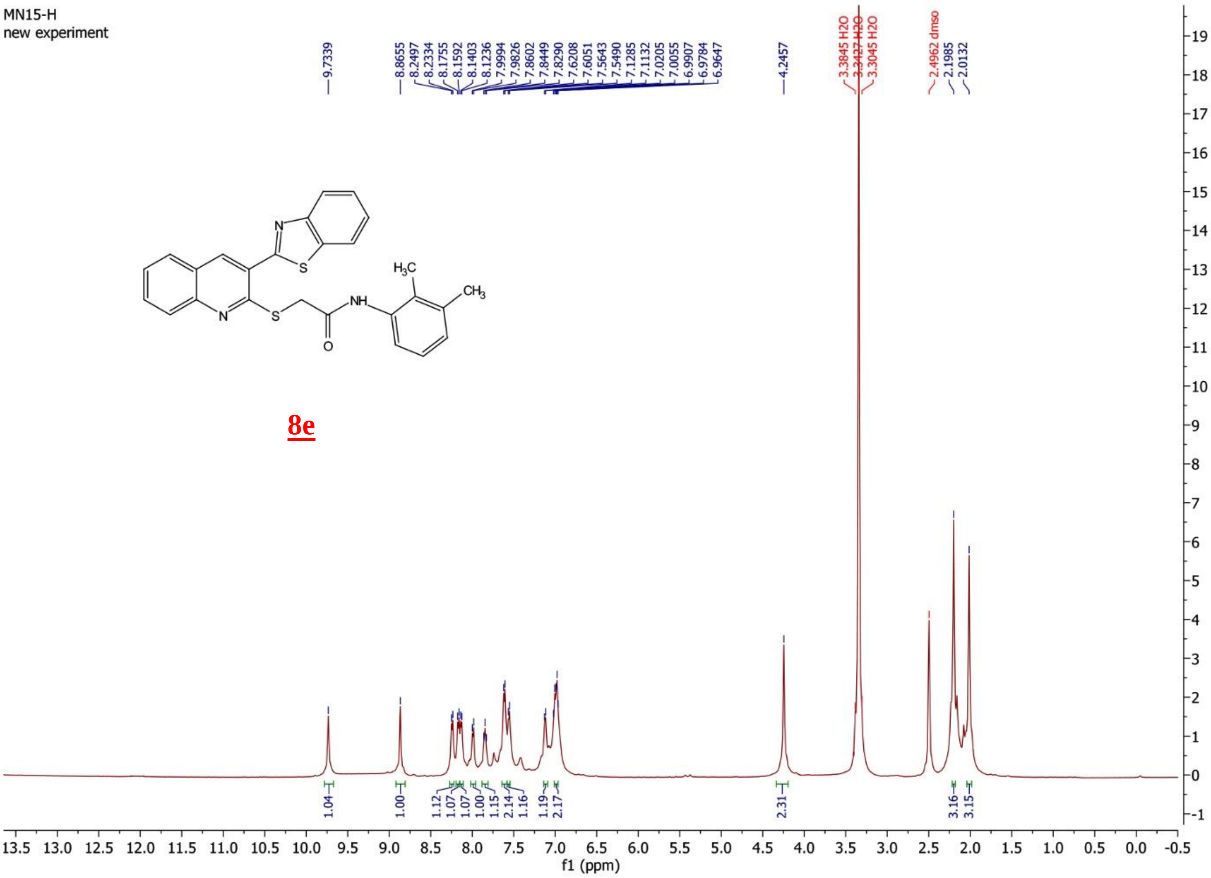

MN15-H
new experiment
9.7339
8.8655
8.2497
8.2334
8.1755
8.1592
8.1403
8.1236
7.9994
7.9826
7.8602
7.8449
7.8290
7.6208
7.6051
7.5643
7.5490
7.1285
7.1132
7.0205
7.0055
6.9907
6.9784
6.9647
4.2457
3.3845 H2O
3.3427 H2O
3.3045 H2O
2.4962 dmso
2.1985
2.0132
H3C
CH3
N
S
NH
O
8e
1.04
1.00
1.12
1.07
1.07
1.00
1.15
2.14
1.16
1.19
2.17
2.31
3.16
3.15
f1 (ppm)

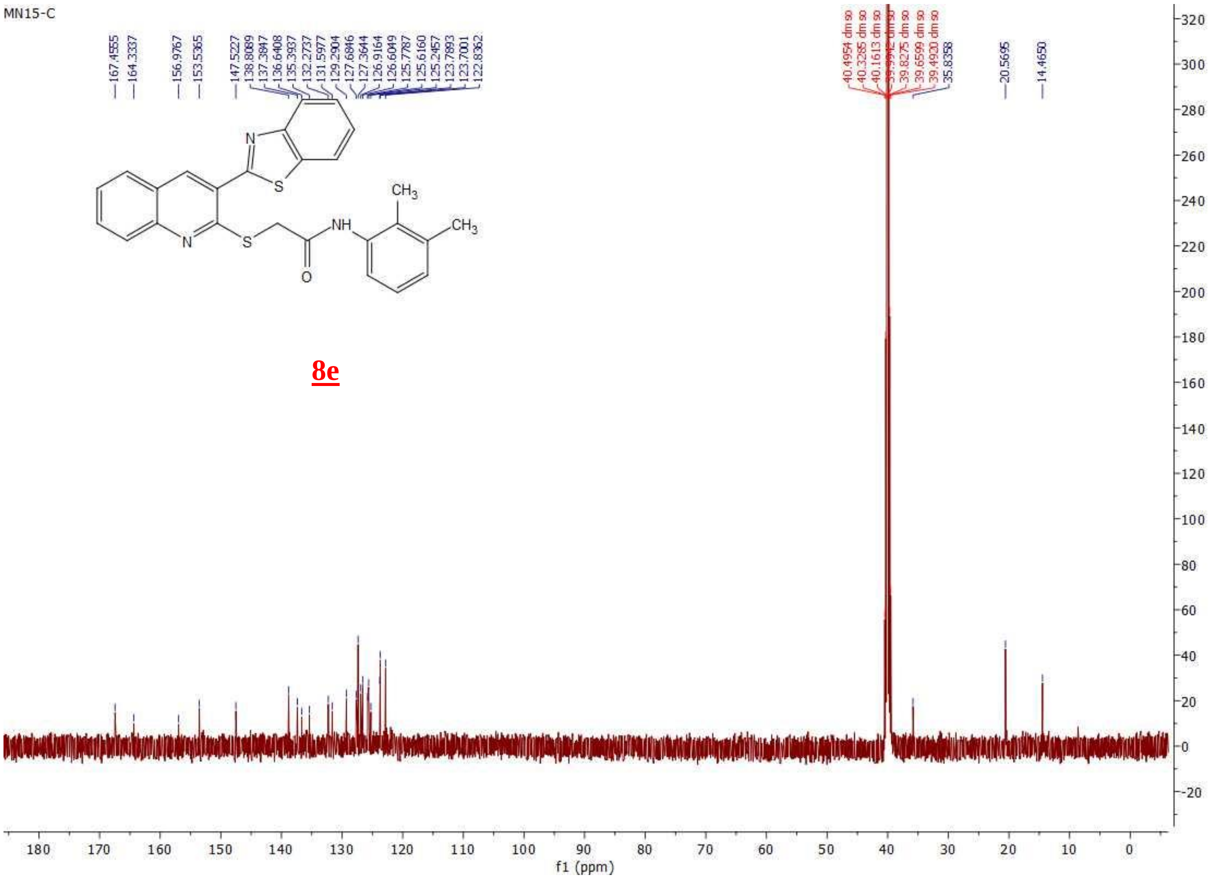

MN15-C
167.4555
164.3337
156.9767
153.5365
147.5227
138.8089
137.3847
136.6408
135.3937
132.2737
131.5977
129.2904
127.6846
127.3644
126.9164
126.6049
125.7787
125.6160
125.2457
123.7893
123.7001
122.8362
40.4954 dmso
40.3285 dmso
40.1613 dmso
dmso
39.8275 dmso
39.6599 dmso
39.4930 dmso
35.8358
20.5695
14.4650
N
S
N
S
NH
O
CH3
CH3
f1 (ppm)

8e

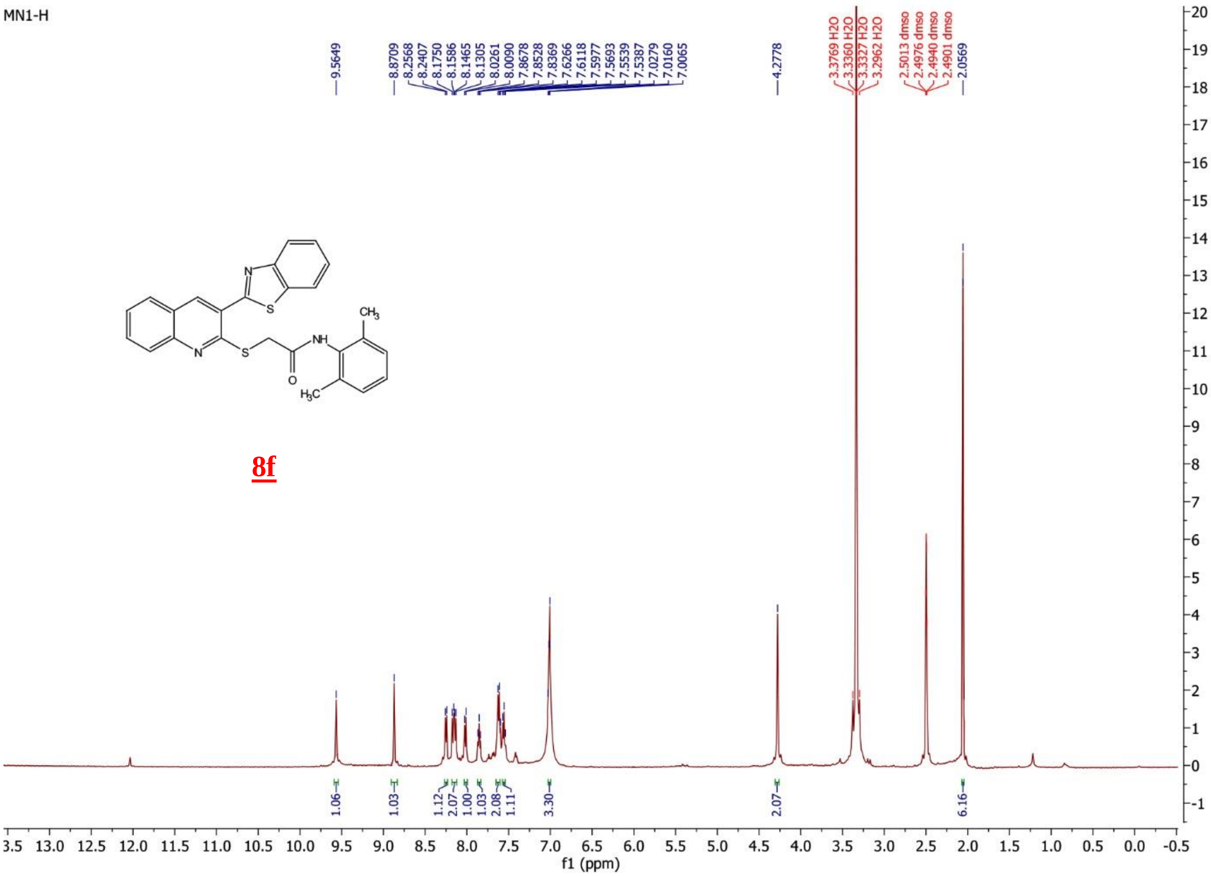
MN1-H
9.5649
8.8709
8.2568
8.2407
8.1750
8.1586
8.1465
8.1305
8.0261
8.0090
7.8678
7.8528
7.8369
7.6266
7.6118
7.5977
7.5693
7.5539
7.5387
7.0279
7.0160
7.0065
4.2778
3.3769 H2O
3.3360 H2O
3.3327 H2O
3.2962 H2O
2.5013 dmso
2.4976 dmso
2.4940 dmso
2.4901 dmso
2.0569
1.06
1.03
1.12
2.07
1.00
1.03
2.08
1.11
3.30
2.07
6.16
8f
f1 (ppm)

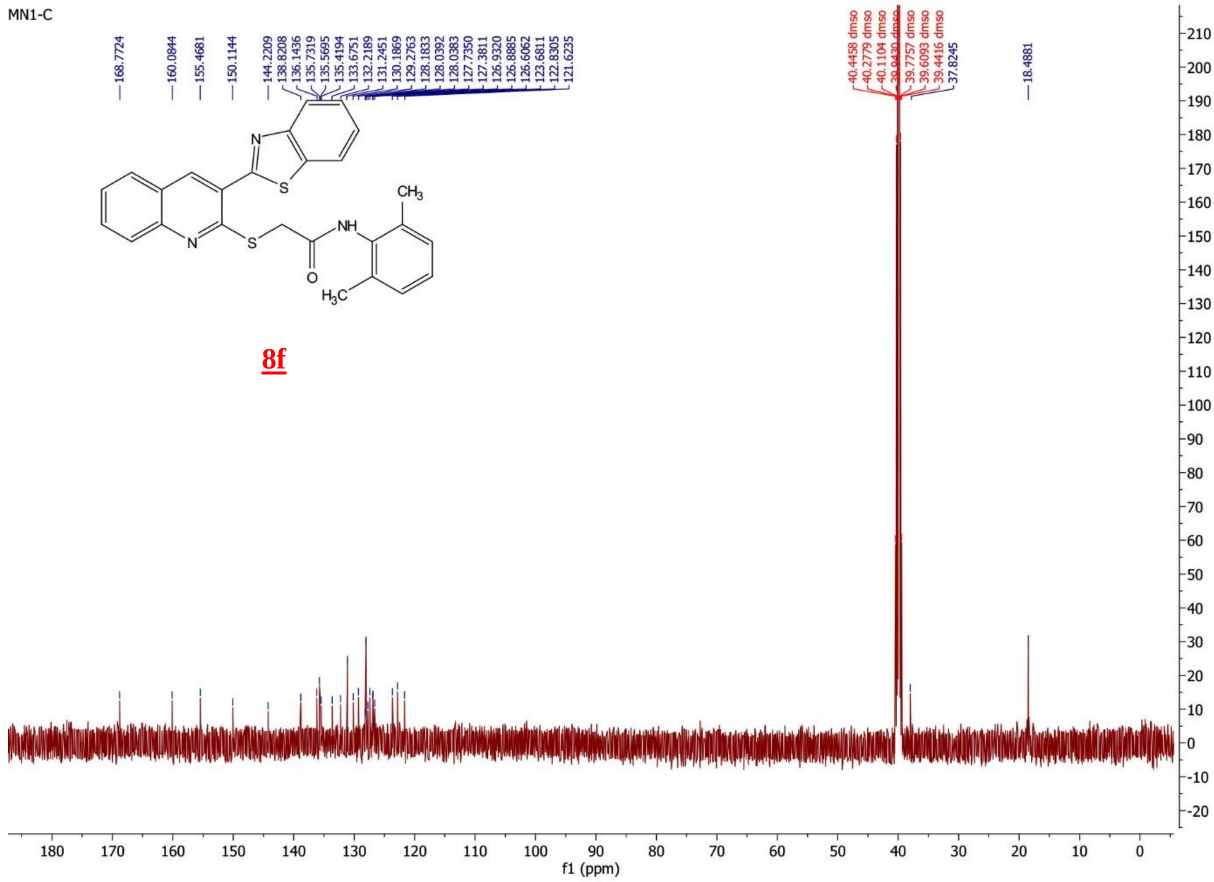
MN1-C
168.7724
160.0844
155.4681
150.1144
144.2209
138.8208
136.1436
135.7319
135.5695
135.4194
133.6751
132.2189
131.2451
130.1869
129.2763
128.1833
128.0392
128.0383
127.7350
127.3811
126.9320
126.8885
126.6062
123.6811
122.8305
121.6235
40.4458 dmso
40.2779 dmso
40.1104 dmso
39.9430 dmso
39.7757 dmso
39.6093 dmso
39.4416 dmso
37.8245
18.4881
N
S
N
S
NH
O
CH3
H3C
8f
f1 (ppm)

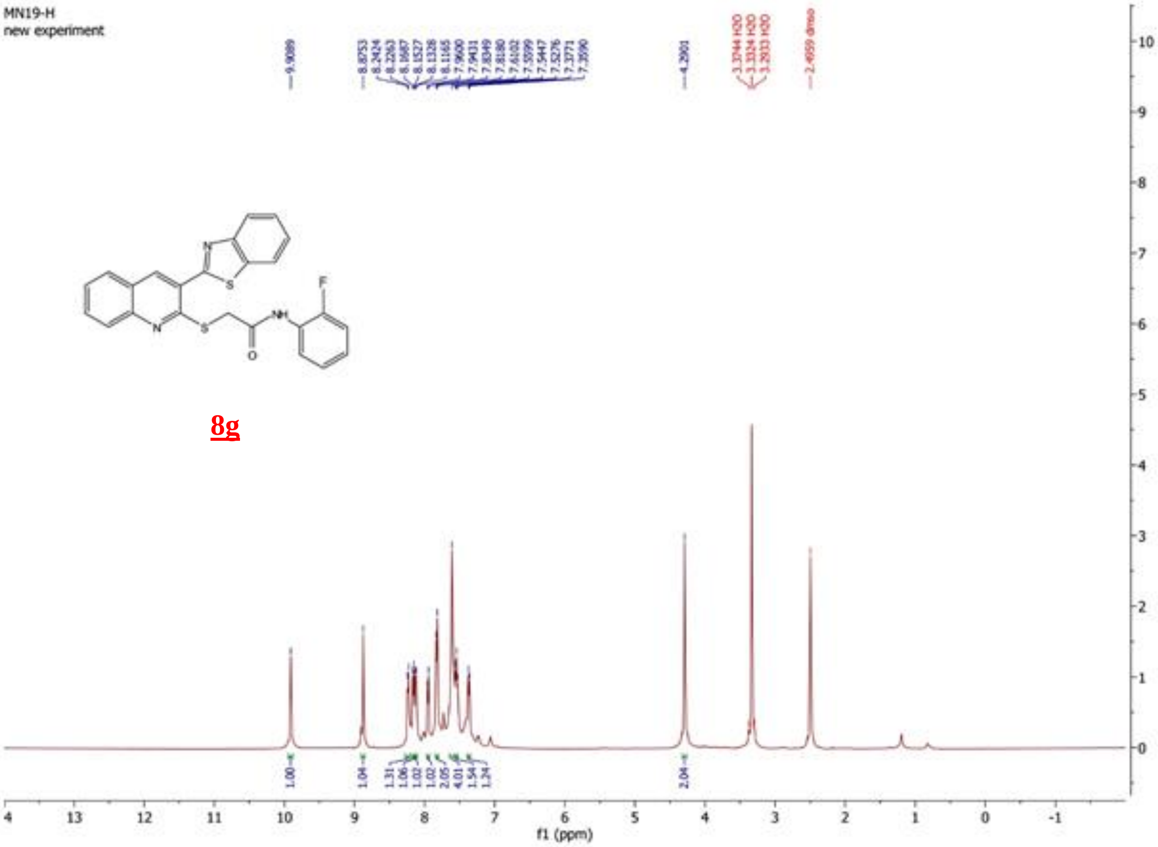

MN19-H
new experiment
9.9089
8.8753
8.2424
8.2263
8.1687
8.1527
8.1328
8.1165
7.9600
7.9431
7.8349
7.8180
7.6102
7.5599
7.5447
7.5276
7.3771
7.3590
4.2901
3.3744 H2O
3.3324 H2O
3.2933 H2O
2.4959 dmso
N
S
F
NH
N
S
O
1.00
1.04
1.31
1.06
1.02
1.02
2.05
4.01
1.54
1.34
2.04
f1 (ppm)

8g

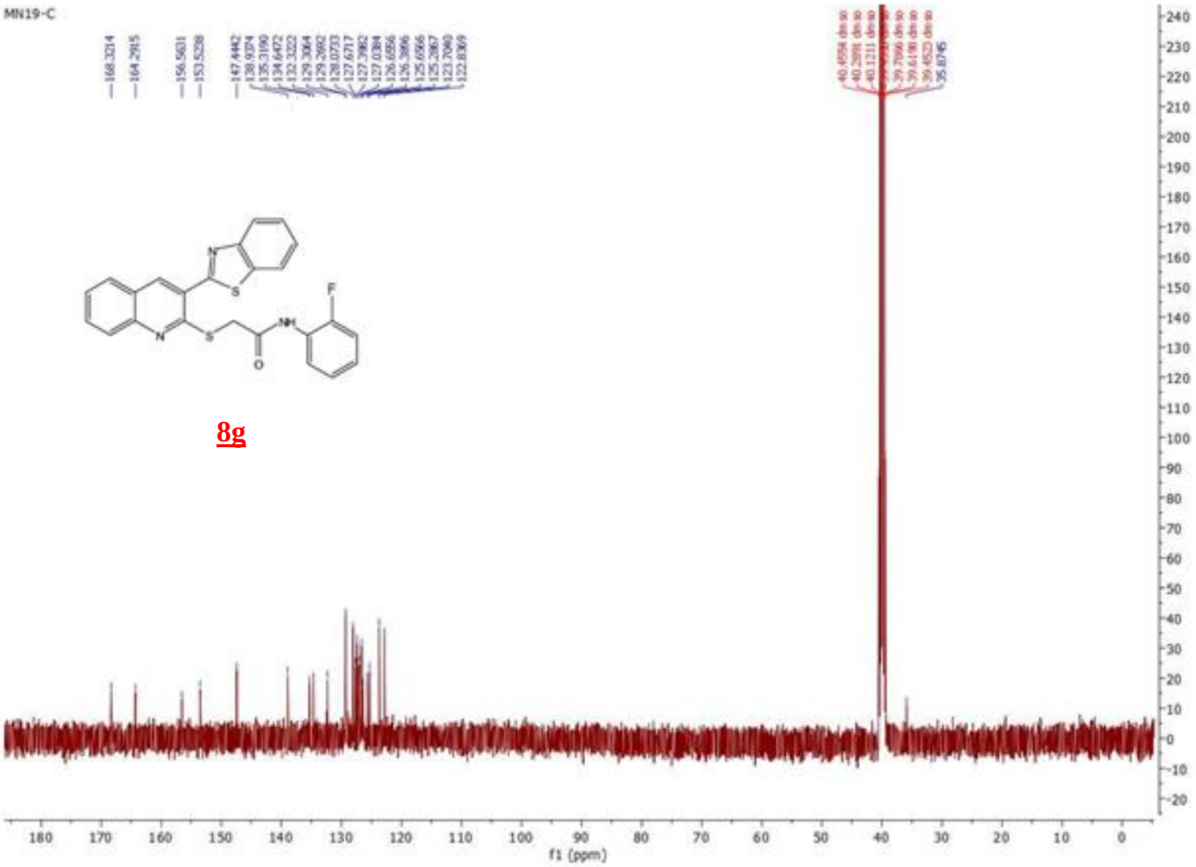
MN19-C
168.3214
164.2915
156.5631
153.5238
147.4442
138.9374
135.3190
134.6472
132.3222
129.3064
129.2692
128.0733
127.6717
127.3982
127.0384
126.6556
126.3866
125.6566
125.2867
123.7040
122.8369
40.4556 dmso
40.2891 dmso
40.1211 dmso
39.7966 dmso
39.6198 dmso
39.4523 dmso
35.8745
N
S
NH
F
O
f1 (ppm)

8g

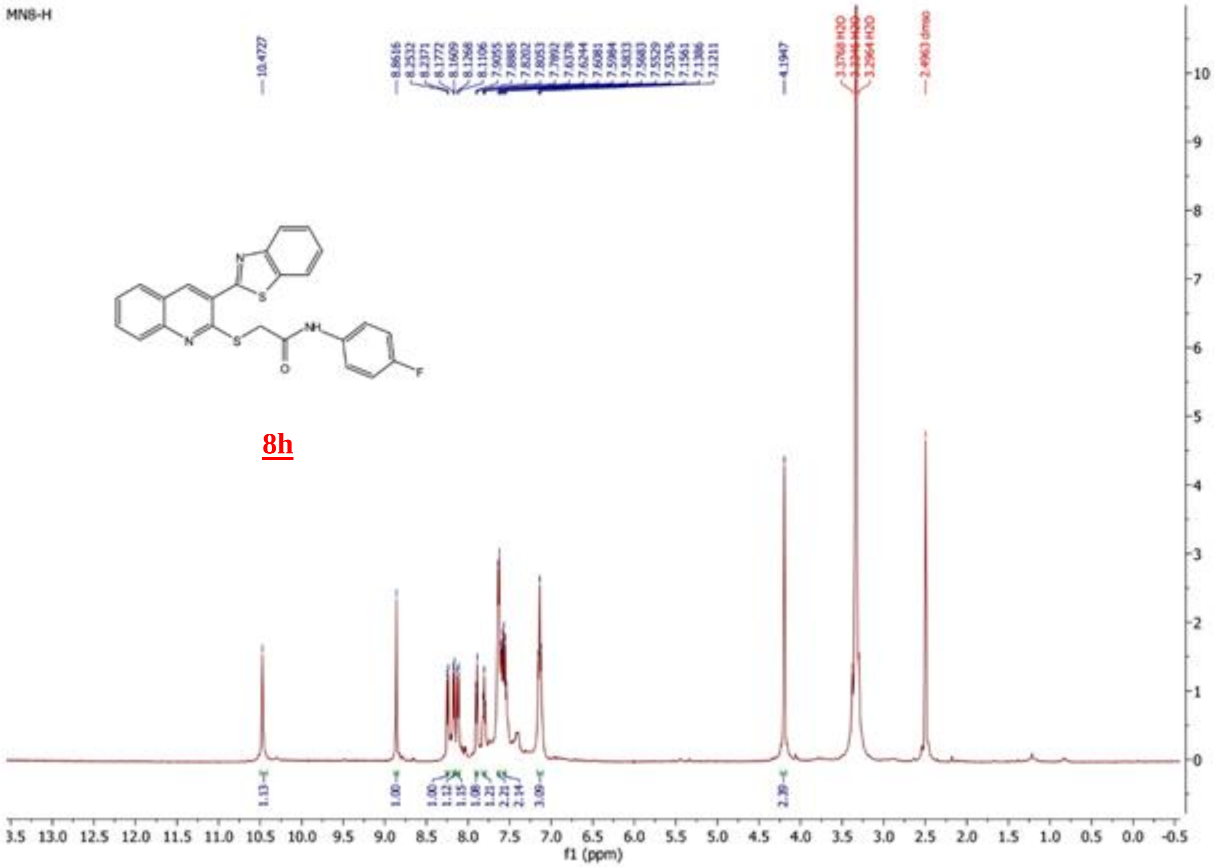

8h

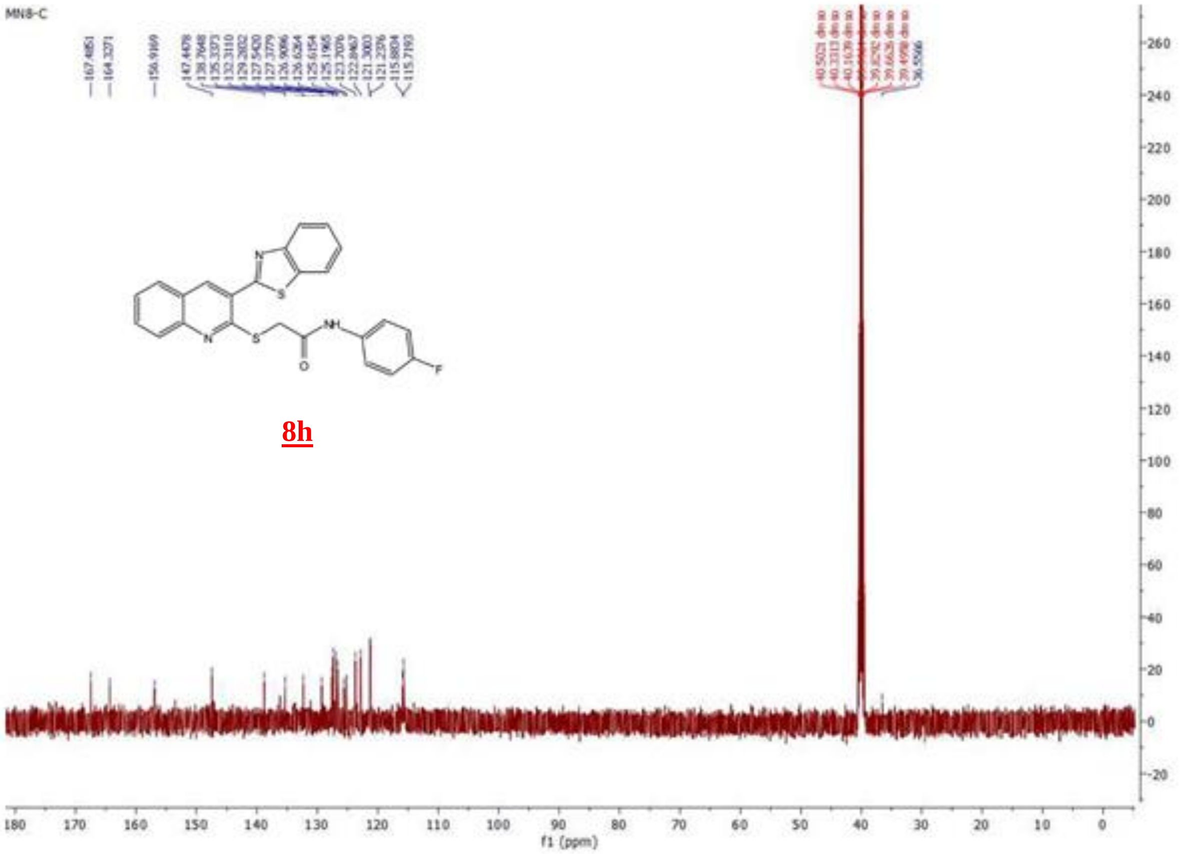
MN8-C
167.4851
164.3271
156.9169
8h
f1 (ppm)

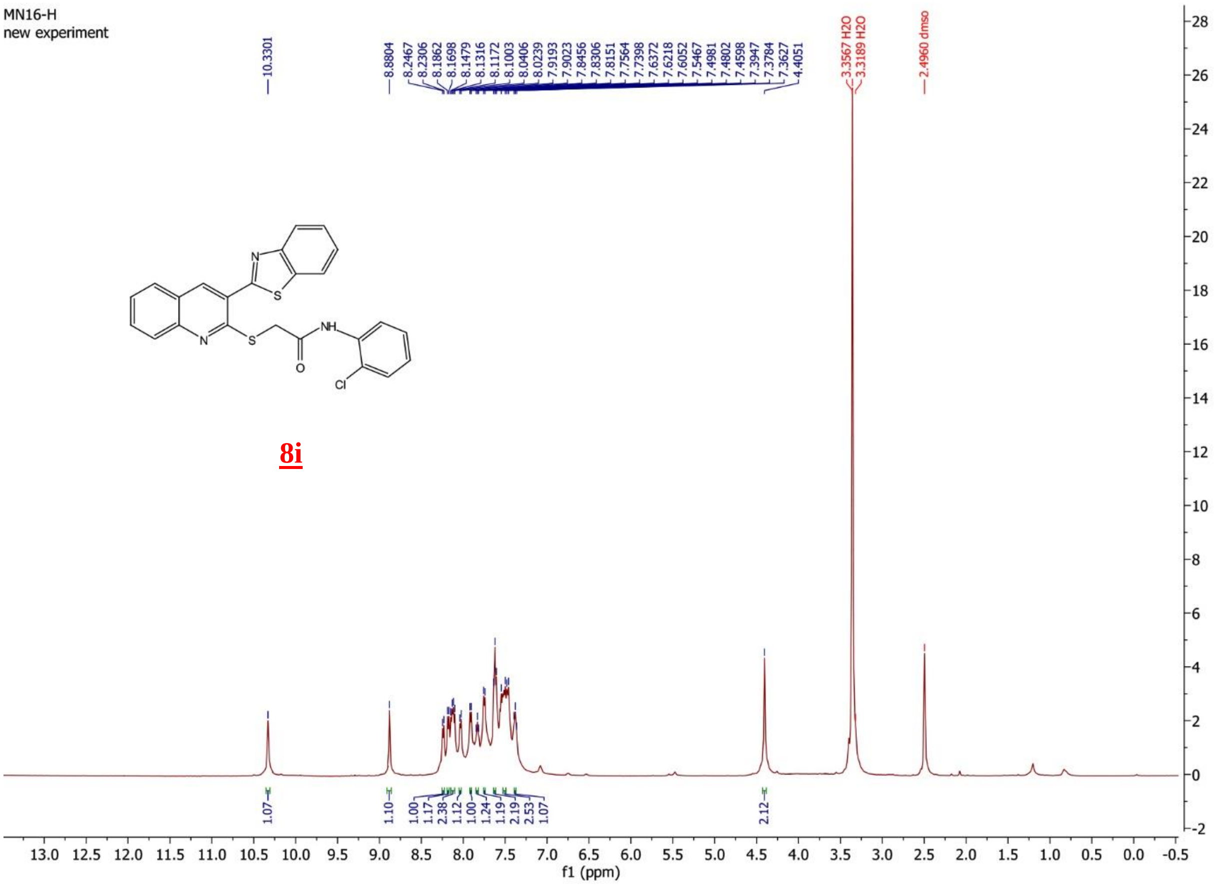

MN16-H
new experiment
10.3301
8.8804
8.2467
8.2306
8.1862
8.1698
8.1479
8.1316
8.1172
8.1003
8.0406
8.0239
7.9193
7.9023
7.8456
7.8306
7.8151
7.7564
7.7398
7.6372
7.6218
7.6052
7.5467
7.4981
7.4802
7.4598
7.3947
7.3784
7.3627
4.4051
3.3567 H2O
3.3189 H2O
2.4960 dmso
N
S
NH
O
Cl
8i
1.07
1.10
1.00
1.17
2.38
1.12
1.00
1.24
1.19
2.19
2.53
1.07
2.12
f1 (ppm)

MN16-C

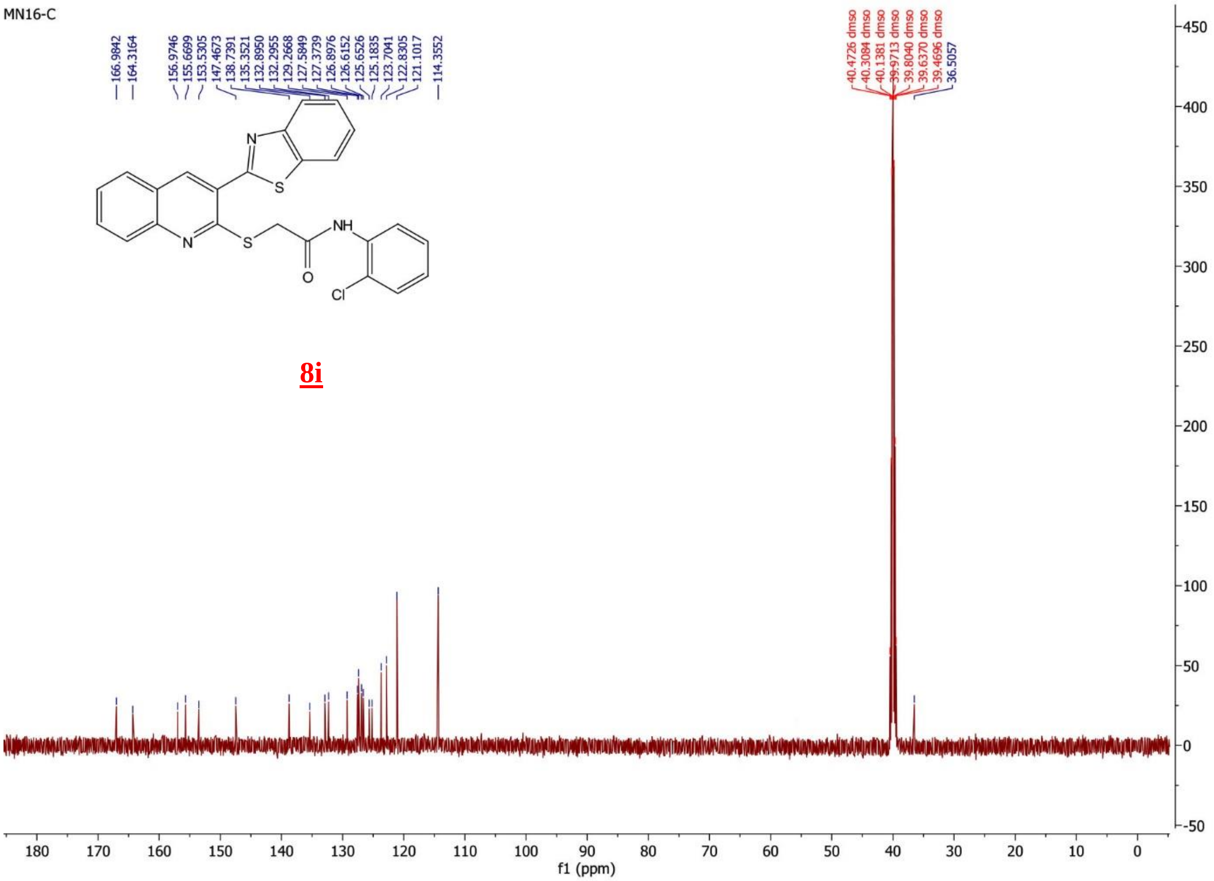
166.9842
164.3164
156.9746
155.6699
153.5305
147.4673
138.7391
135.3521
132.8950
132.2955
129.2668
127.5849
127.3739
126.8976
126.6152
125.6526
125.1835
123.7041
122.8305
121.1017
114.3552
40.4726 dmso
40.3084 dmso
40.1381 dmso
39.9713 dmso
39.8040 dmso
39.6370 dmso
39.4696 dmso
36.5057
N
S
N
S
NH
O
Cl
f1 (ppm)

8i

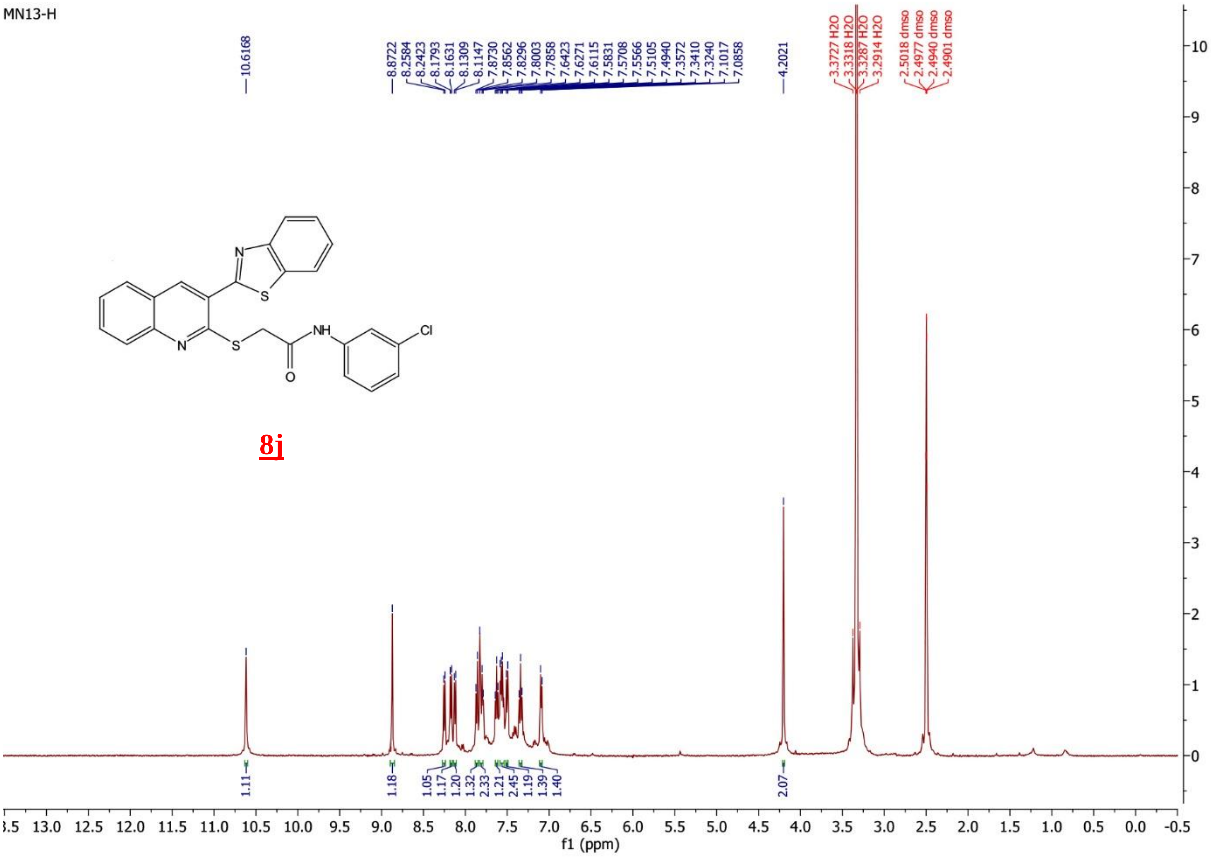

MN13-H
10.6168
8.8722
8.2584
8.2423
8.1793
8.1631
8.1309
8.1147
7.8730
7.8562
7.8296
7.8003
7.7858
7.6423
7.6271
7.6115
7.5831
7.5708
7.5566
7.5105
7.4940
7.3572
7.3410
7.3240
7.1017
7.0858
4.2021
3.3727 H2O
3.3318 H2O
3.3287 H2O
3.2914 H2O
2.5018 dmso
2.4977 dmso
2.4940 dmso
2.4901 dmso
N
S
NH
Cl
O
8j
1.11
1.18
1.05
1.17
1.20
1.32
2.33
1.21
2.45
1.19
1.39
1.40
2.07
f1 (ppm)

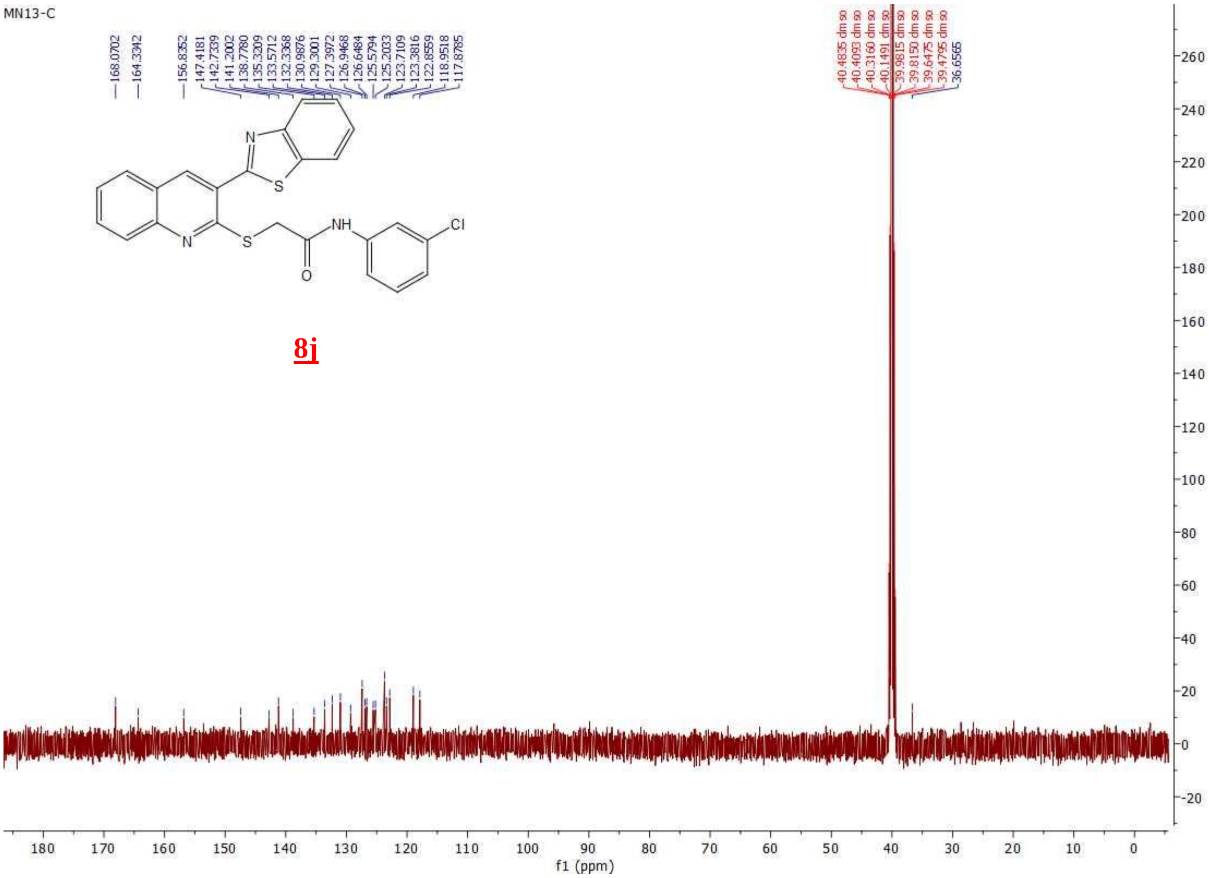
MN13-C
168.0702
164.3342
156.8352
147.4181
142.7339
141.2002
138.7780
135.3209
133.5712
132.3368
130.9876
129.3001
127.3972
126.9468
126.6484
125.5794
125.2033
123.7109
123.3816
122.8559
118.9518
117.8785
40.4835 dmso
40.4093 dmso
40.3160 dmso
40.1491 dmso
39.9815 dmso
39.8150 dmso
39.6475 dmso
39.4795 dmso
36.6565
N
S
N
S
NH
O
Cl
8j
f1 (ppm)

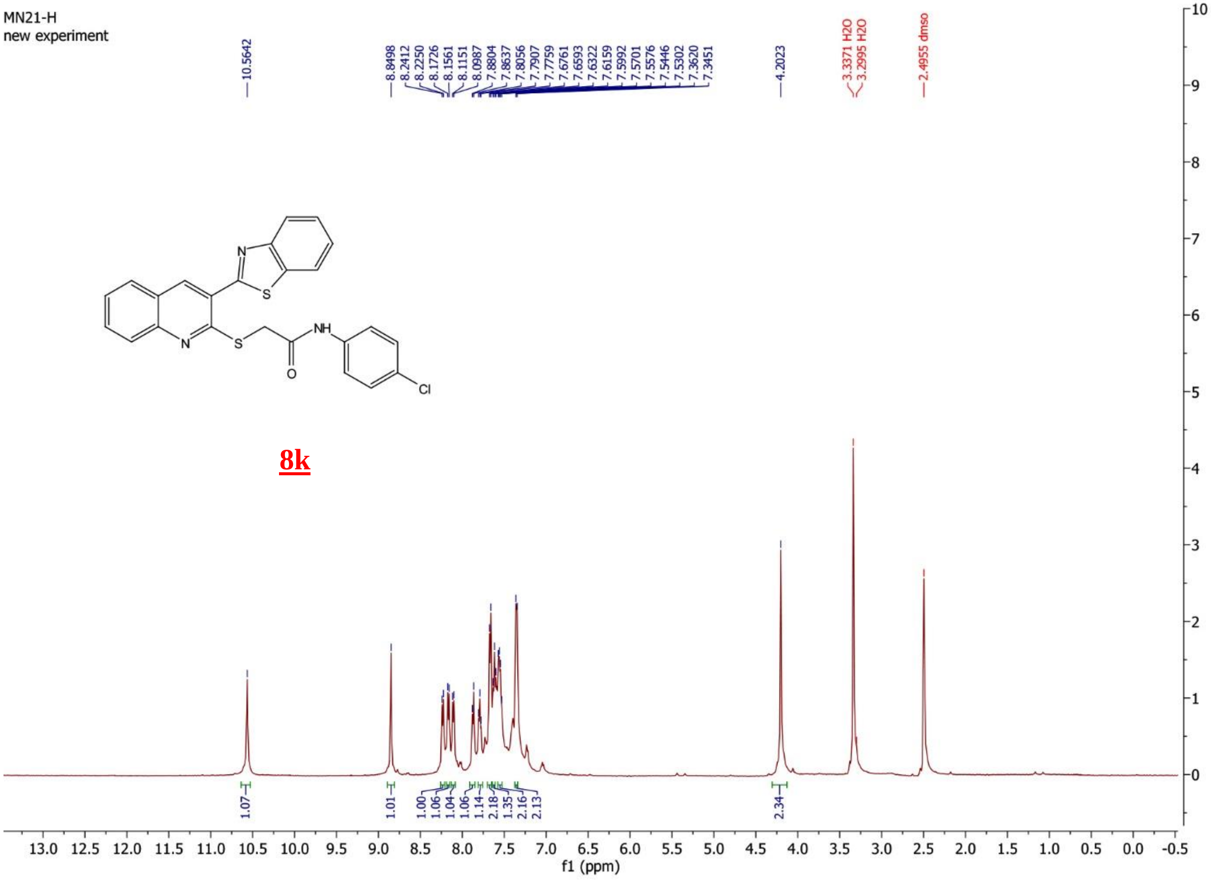
MN21-H
new experiment
10.5642
8.8498
8.2412
8.2250
8.1726
8.1561
8.1151
8.0987
7.8804
7.8637
7.8056
7.7907
7.7759
7.6761
7.6593
7.6322
7.6159
7.5992
7.5701
7.5576
7.5446
7.5302
7.3620
7.3451
4.2023
3.3371 H2O
3.2995 H2O
2.4955 dmso
1.07
1.01
1.00
1.06
1.04
1.06
1.14
2.18
1.35
2.16
2.13
2.34
f1 (ppm)

8k

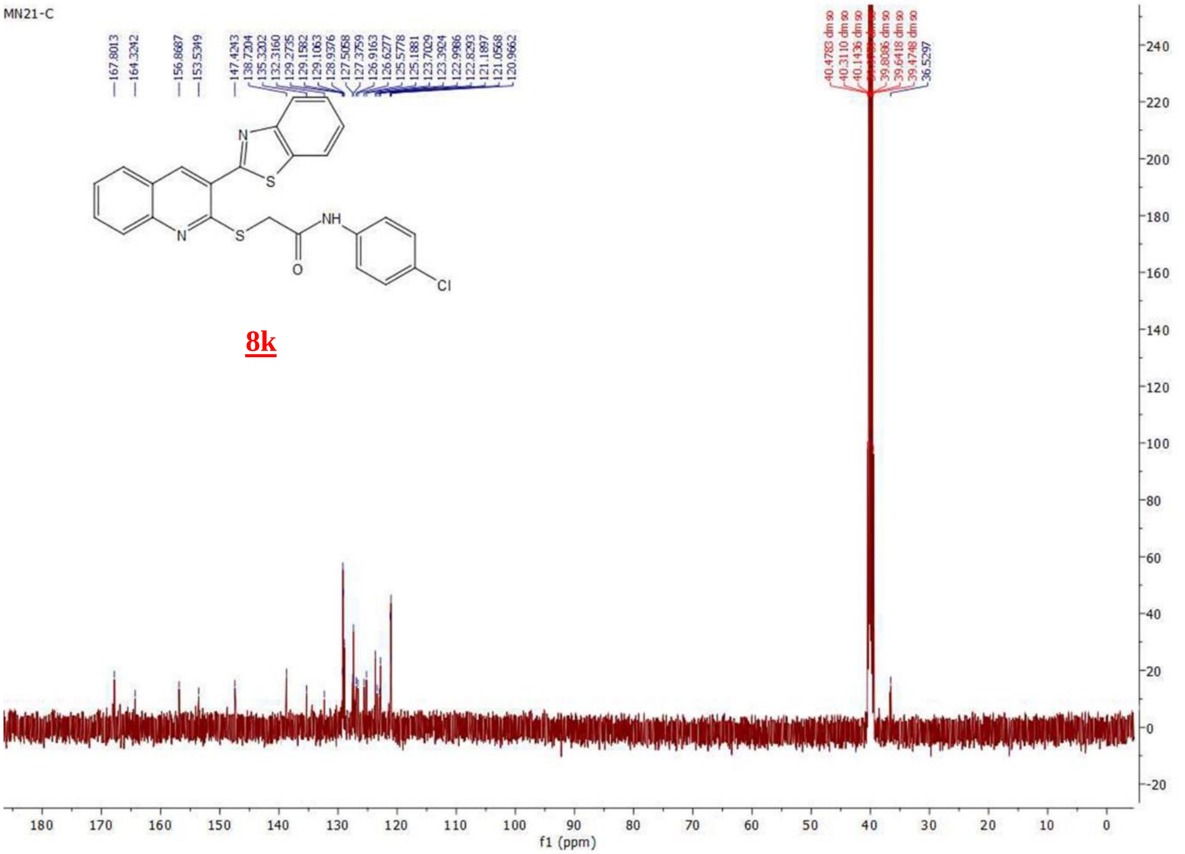
MN21-C
167.8013
164.3242
156.8687
153.5349
147.4243
138.7204
135.3202
132.3160
129.2735
129.1582
129.1063
128.9376
127.5058
127.3759
126.9163
126.6277
125.5778
125.1881
123.7029
123.3924
122.9986
122.8293
121.1897
121.0568
120.9662
40.4783 dm so
40.3110 dm so
40.1436 dm so
39.8096 dm so
39.6418 dm so
39.4748 dm so
36.5297
N
S
N
S
NH
O
Cl
8k
f1 (ppm)

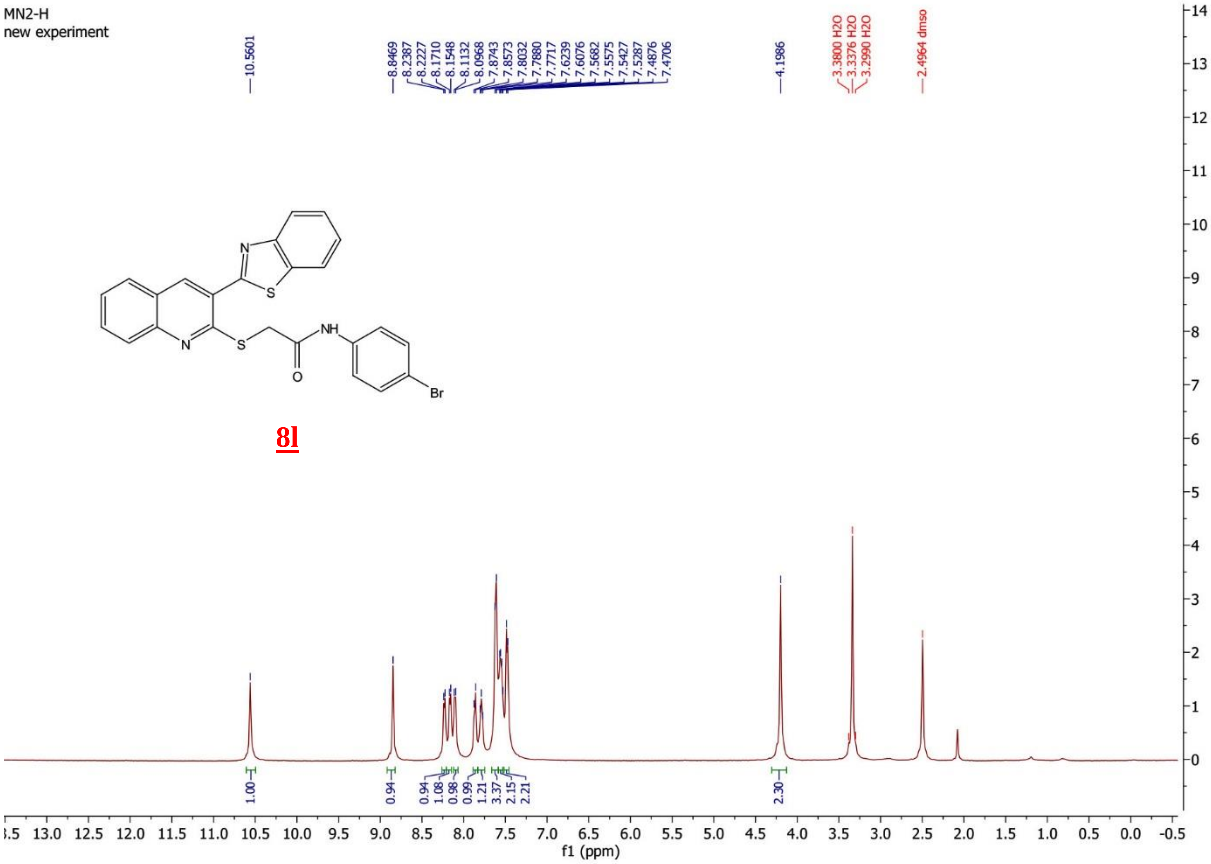

MN2-H
new experiment
10.5601
8.8469
8.2387
8.2227
8.1710
8.1548
8.1132
8.0968
7.8743
7.8573
7.8032
7.7880
7.7717
7.6239
7.6076
7.5682
7.5575
7.5427
7.5287
7.4876
7.4706
4.1986
3.3800 H2O
3.3376 H2O
3.2990 H2O
2.4964 dmso
1.00
0.94
0.94
1.08
0.98
0.99
1.21
3.37
2.15
2.21
2.30
f1 (ppm)
N
S
NH
O
Br

8l

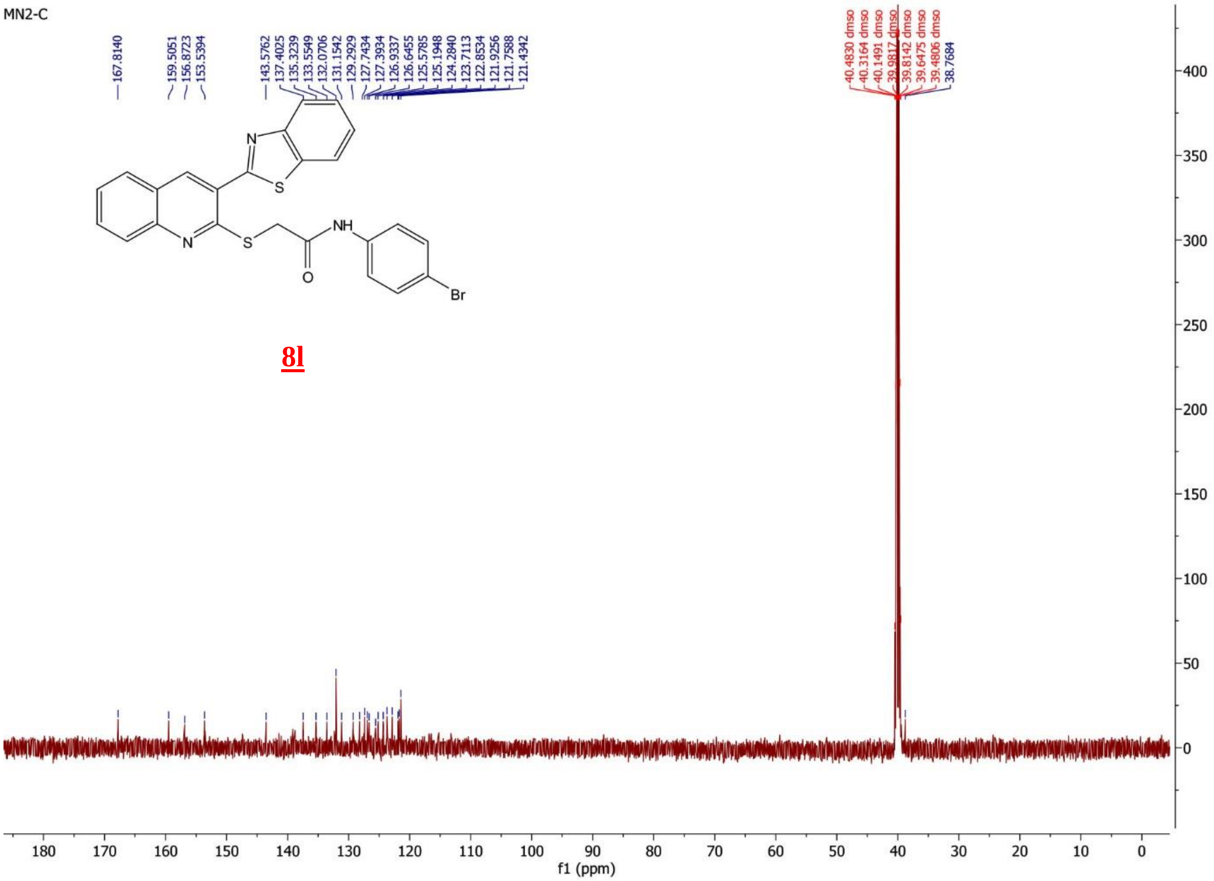
MN2-C
167.8140
159.5051
156.8723
153.5394
143.5762
137.4025
135.3239
133.5549
132.0706
131.1542
129.2929
127.7434
127.3934
126.9337
126.6455
125.5785
125.1948
124.2840
123.7113
122.8534
121.9256
121.7588
121.4342
40.4830 dmso
40.3164 dmso
40.1491 dmso
39.9817 dmso
39.8142 dmso
39.6475 dmso
39.4806 dmso
38.7684
N
S
N
S
NH
O
Br
8l
f1 (ppm)

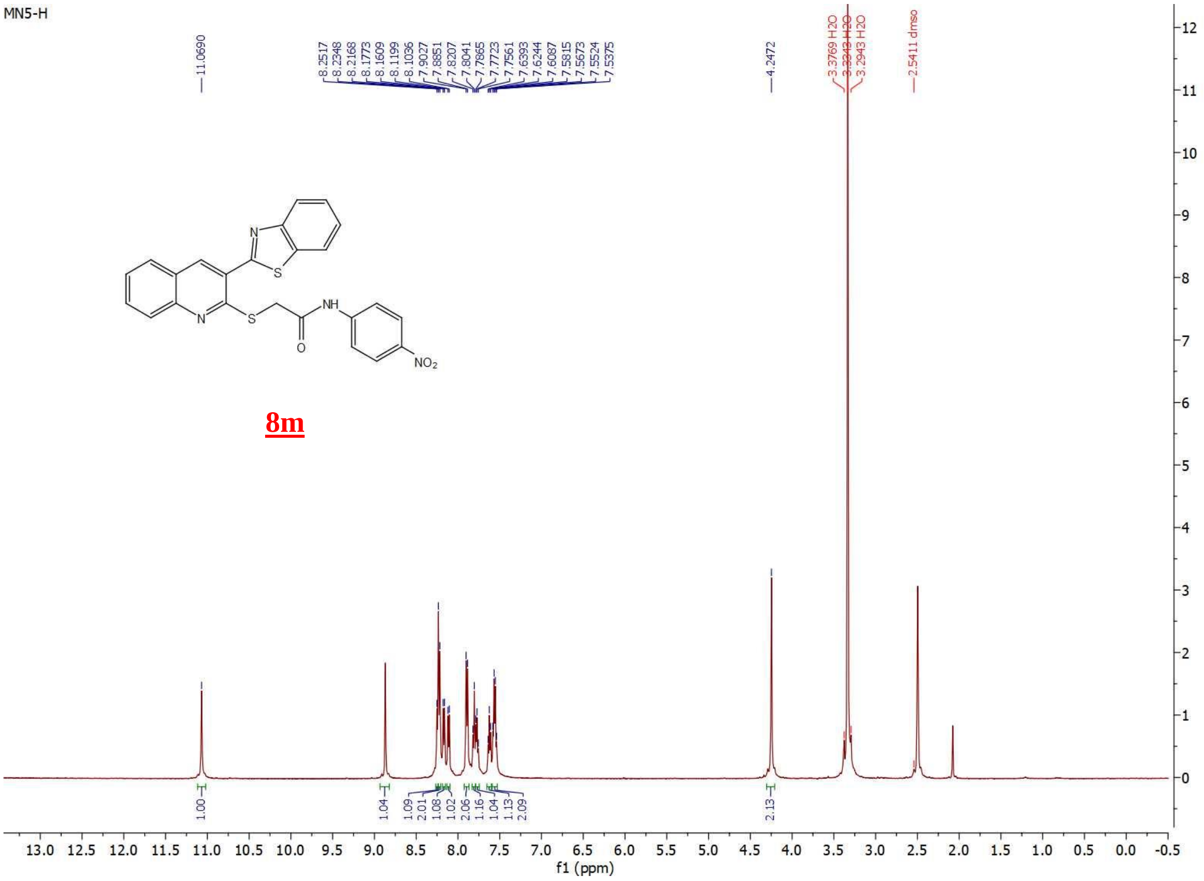
MN5-H
11.0690
8.2517
8.2348
8.2168
8.1773
8.1609
8.1199
8.1036
7.9027
7.8851
7.8207
7.8041
7.7865
7.7723
7.7561
7.6393
7.6244
7.6087
7.5815
7.5673
7.5524
7.5375
4.2472
3.3769 H2O
3.3343 H2O
3.2943 H2O
2.5411 dmso
N
S
N
S
NH
O
NO2
8m
1.00
1.04
1.09
2.01
1.08
1.02
2.06
1.16
1.04
1.13
2.09
2.13
13.0 12.5 12.0 11.5 11.0 10.5 10.0 9.5 9.0 8.5 8.0 7.5 7.0 6.5 6.0 5.5 5.0 4.5 4.0 3.5 3.0 2.5 2.0 1.5 1.0 0.5 0.0 -0.5
f1 (ppm)
12 11 10 9 8 7 6 5 4 3 2 1 0

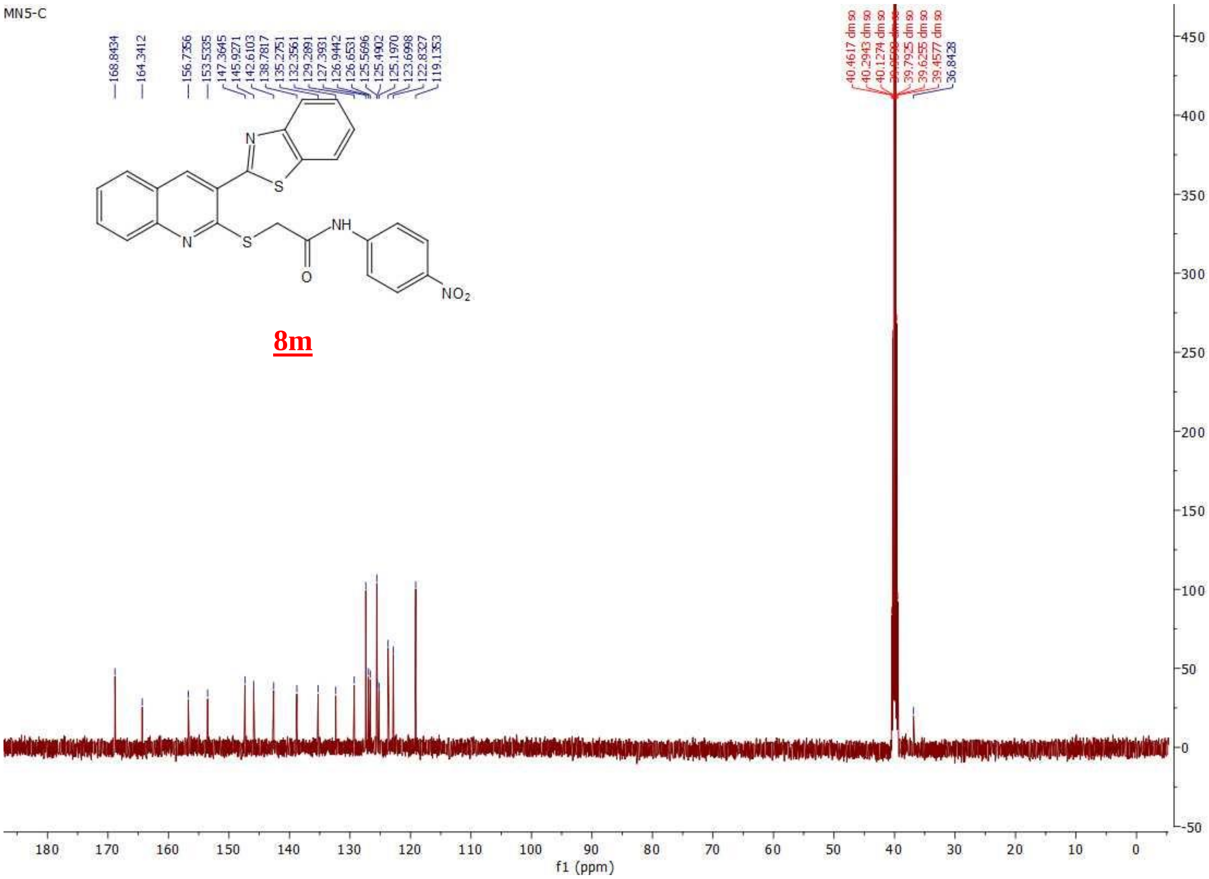
MN5-C
168.8434
164.3412
156.7356
153.5335
147.3645
145.9271
142.6103
138.7817
135.2751
132.3561
129.2891
127.3931
126.9442
126.6531
125.5696
125.4902
125.1970
123.6998
122.8327
119.1353
40.4617 dmso
40.2943 dmso
40.1274 dmso
39.7925 dmso
39.6255 dmso
39.4577 dmso
36.8428
N
S
N
S
NH
O
NO2
f1 (ppm)

8m

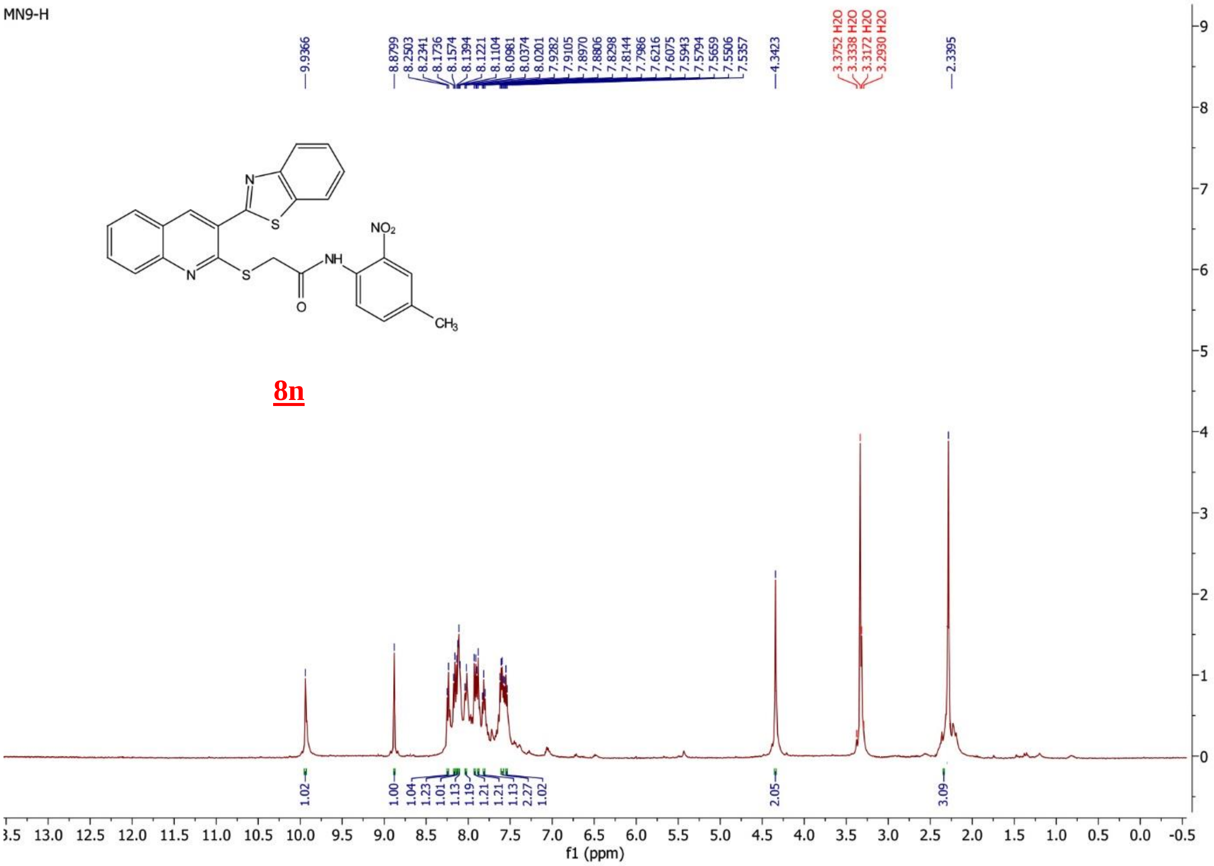
MN9-H
9.9366
8.8799
8.2503
8.2341
8.1736
8.1574
8.1394
8.1221
8.1104
8.0981
8.0374
8.0201
7.9282
7.9105
7.8970
7.8806
7.8298
7.8144
7.7986
7.6216
7.6075
7.5943
7.5794
7.5659
7.5506
7.5357
4.3423
3.3752 H2O
3.3338 H2O
3.3172 H2O
3.2930 H2O
2.3395
NO2
NH
N
S
O
CH3
1.02
1.00
1.04
1.23
1.01
1.13
1.19
1.21
1.21
1.13
2.27
1.02
2.05
3.09
f1 (ppm)

8n

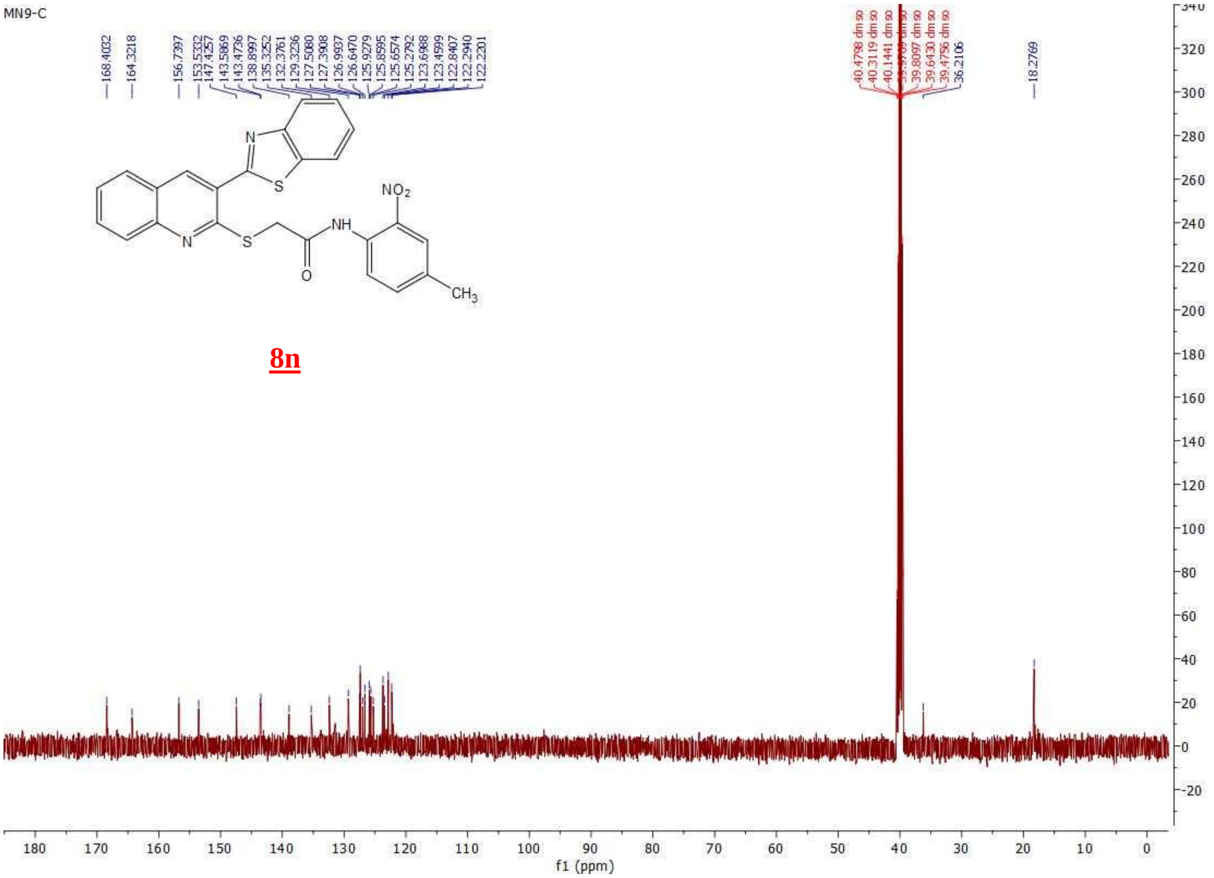

MN9-C
168.4032
164.3218
156.7397
153.5332
147.4257
143.5869
143.4736
138.8997
135.3252
132.3761
129.3236
127.5080
127.3908
126.9937
126.6470
125.9279
125.8595
125.6574
125.2792
123.6988
123.4599
122.8407
122.2940
122.2201
40.4798 dmso
40.3119 dmso
40.1441 dmso
39.8097 dmso
39.6430 dmso
39.4756 dmso
36.2106
18.2769
N
S
NO2
NH
O
CH3
f1 (ppm)

8n

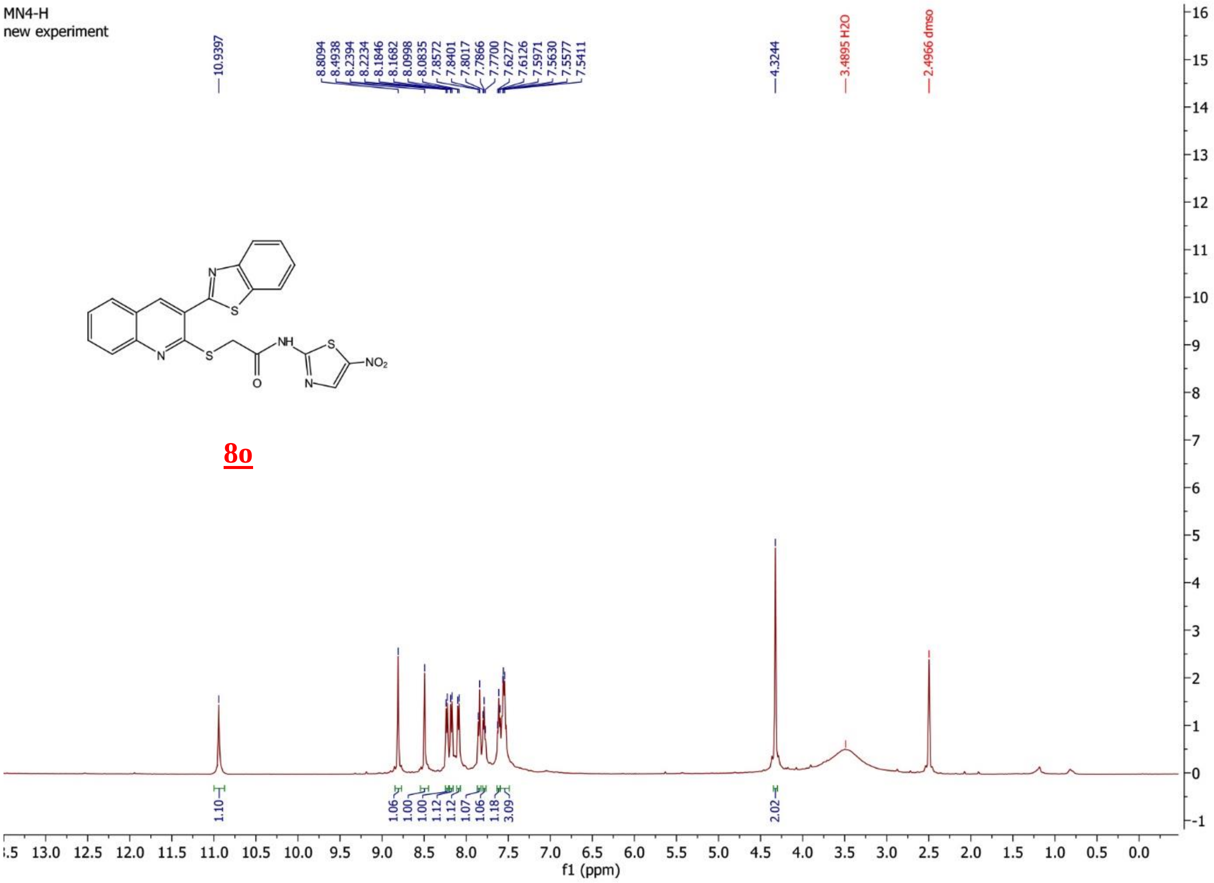
MN4-H
new experiment
10.9397
8.8094
8.4938
8.2394
8.2234
8.1846
8.1682
8.0998
8.0835
7.8572
7.8401
7.8017
7.7866
7.7700
7.6277
7.6126
7.5971
7.5630
7.5577
7.5411
4.3244
3.4895 H2O
2.4966 dmso
8o
1.10
1.06
1.00
1.00
1.12
1.12
1.07
1.06
1.18
3.09
2.02
f1 (ppm)

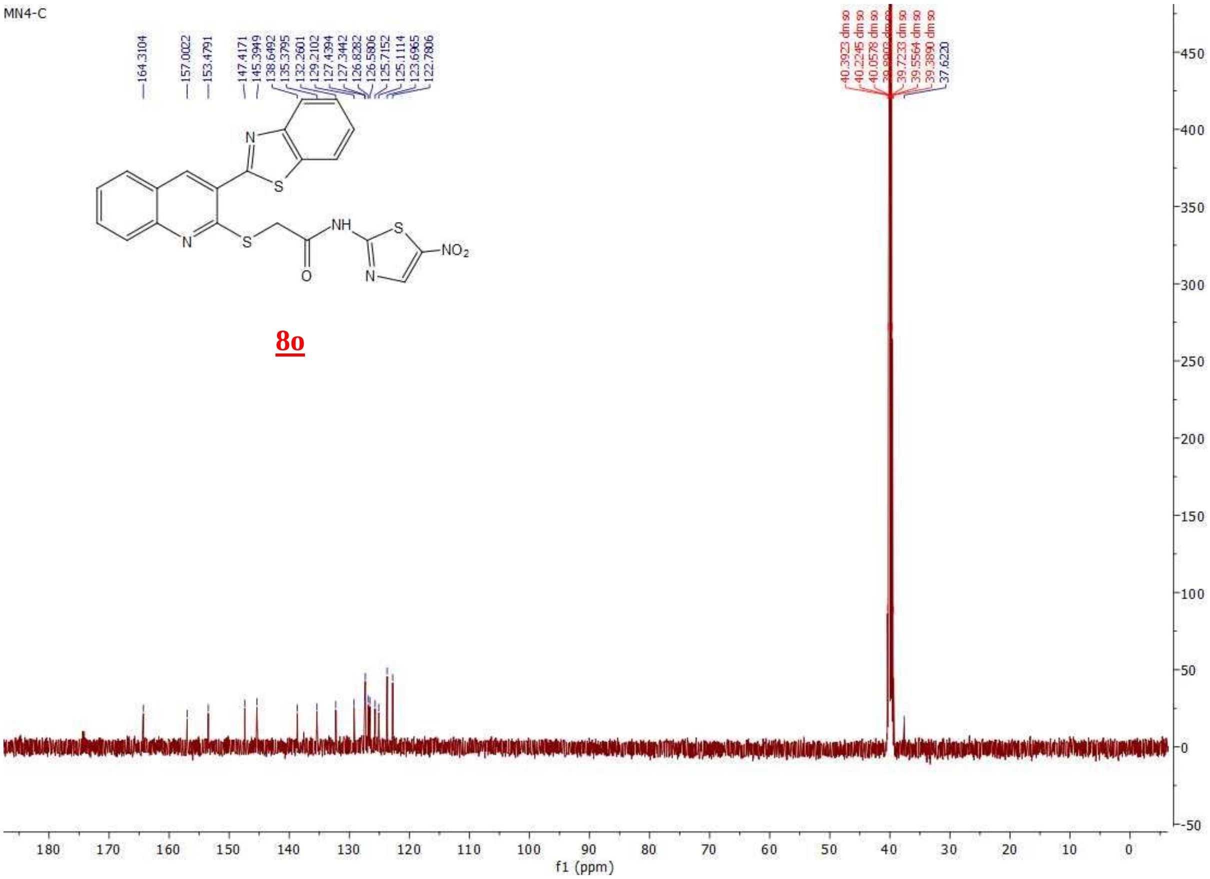
MN4-C
164.3104
157.0022
153.4791
147.4171
145.3949
138.6492
135.3795
132.2601
129.2102
127.4394
127.3442
126.8282
126.5806
125.7152
125.1114
123.6965
122.7806
40.3923 dmso
40.2245 dmso
40.0578 dmso
39.7233 dmso
39.5564 dmso
39.3890 dmso
37.6220
NH
S
N
NO2
O
f1 (ppm)

8o

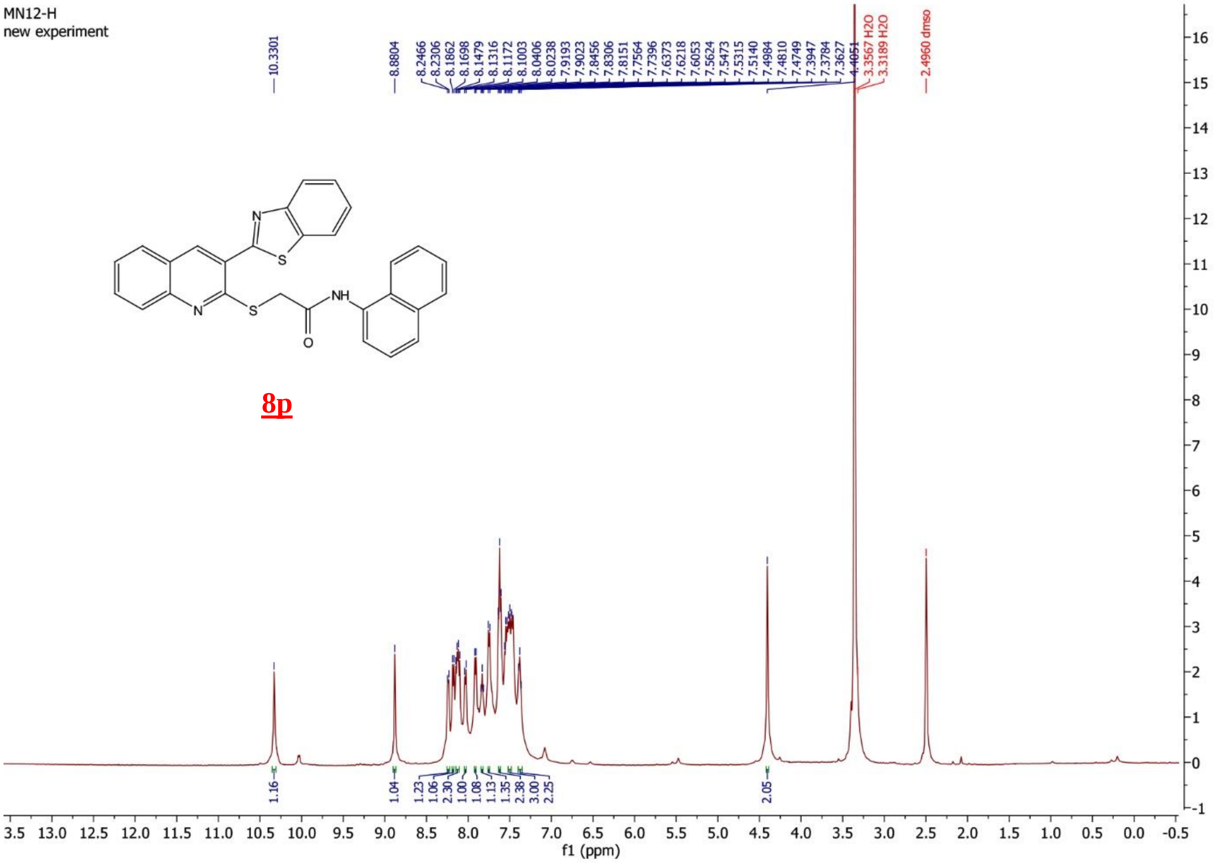
MN12-H
new experiment
10.3301
8.8804
8.2466
8.2306
8.1862
8.1698
8.1479
8.1316
8.1172
8.1003
8.0406
8.0238
7.9193
7.9023
7.8456
7.8306
7.8151
7.7564
7.7396
7.6373
7.6218
7.6053
7.5624
7.5473
7.5315
7.5140
7.4984
7.4810
7.4749
7.3947
7.3784
7.3627
4.4051
3.3567 H2O
3.3189 H2O
2.4960 dmso
N
S
N
S
NH
O
8p
1.16
1.04
1.23
1.06
2.30
1.00
1.08
1.13
1.35
2.38
3.00
2.25
2.05
f1 (ppm)

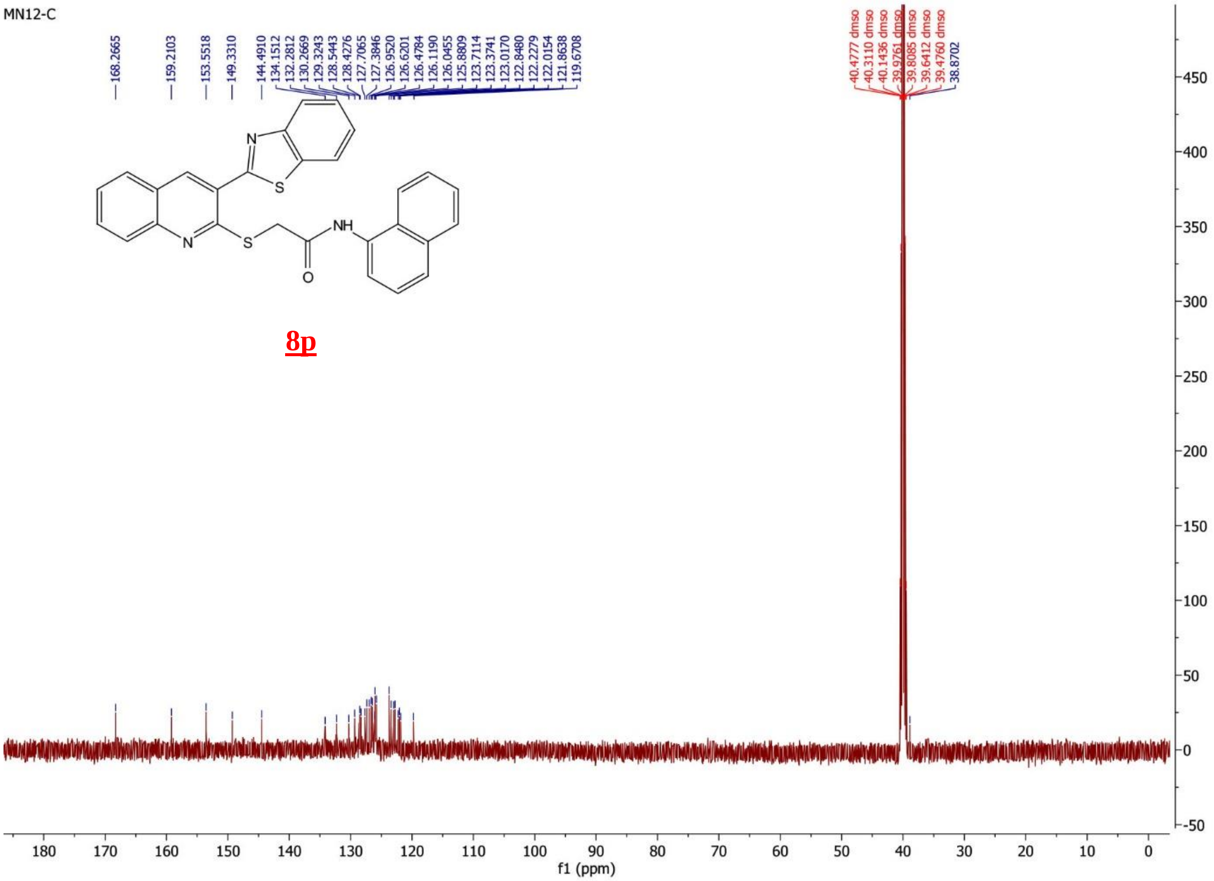
MN12-C
168.2665
159.2103
153.5518
149.3310
144.4910
134.1512
132.2812
130.2669
129.3243
128.5443
128.4276
127.7065
127.3846
126.9520
126.6201
126.4784
126.1190
126.0455
125.8809
123.7114
123.3741
123.0170
122.8480
122.2279
122.0154
121.8638
119.6708
N
S
N
S
NH
O
8p
40.4777 dmso
40.3110 dmso
40.1436 dmso
39.9761 dmso
39.8085 dmso
39.6412 dmso
39.4760 dmso
38.8702
f1 (ppm)

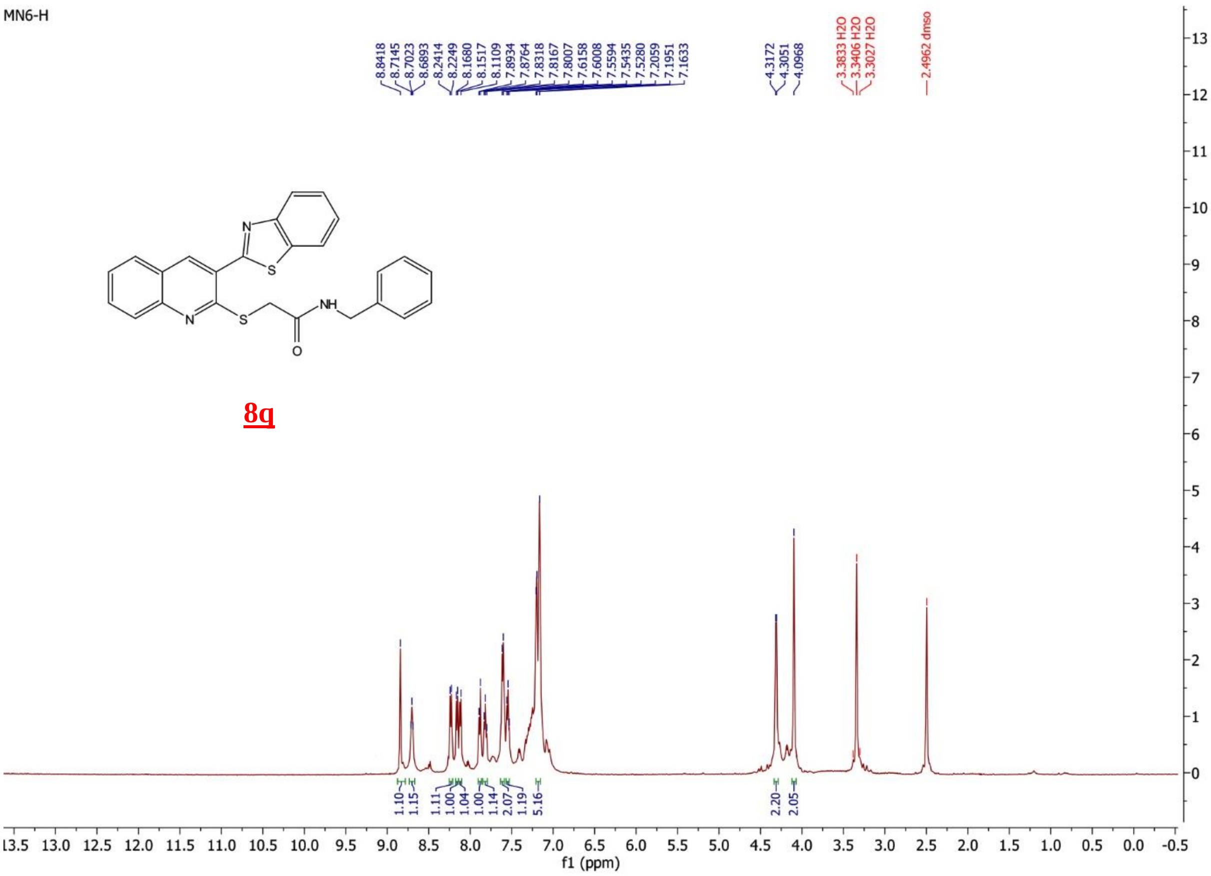
MN6-H
8.8418
8.7145
8.7023
8.6893
8.2414
8.2249
8.1680
8.1517
8.1109
7.8934
7.8764
7.8318
7.8167
7.8007
7.6158
7.6008
7.5594
7.5435
7.5280
7.2059
7.1951
7.1633
4.3172
4.3051
4.0968
3.3833 H2O
3.3406 H2O
3.3027 H2O
2.4962 dmso
N
S
N
S
NH
O
8q
1.10
1.15
1.11
1.00
1.04
1.00
1.14
2.07
1.19
5.16
2.20
2.05
f1 (ppm)

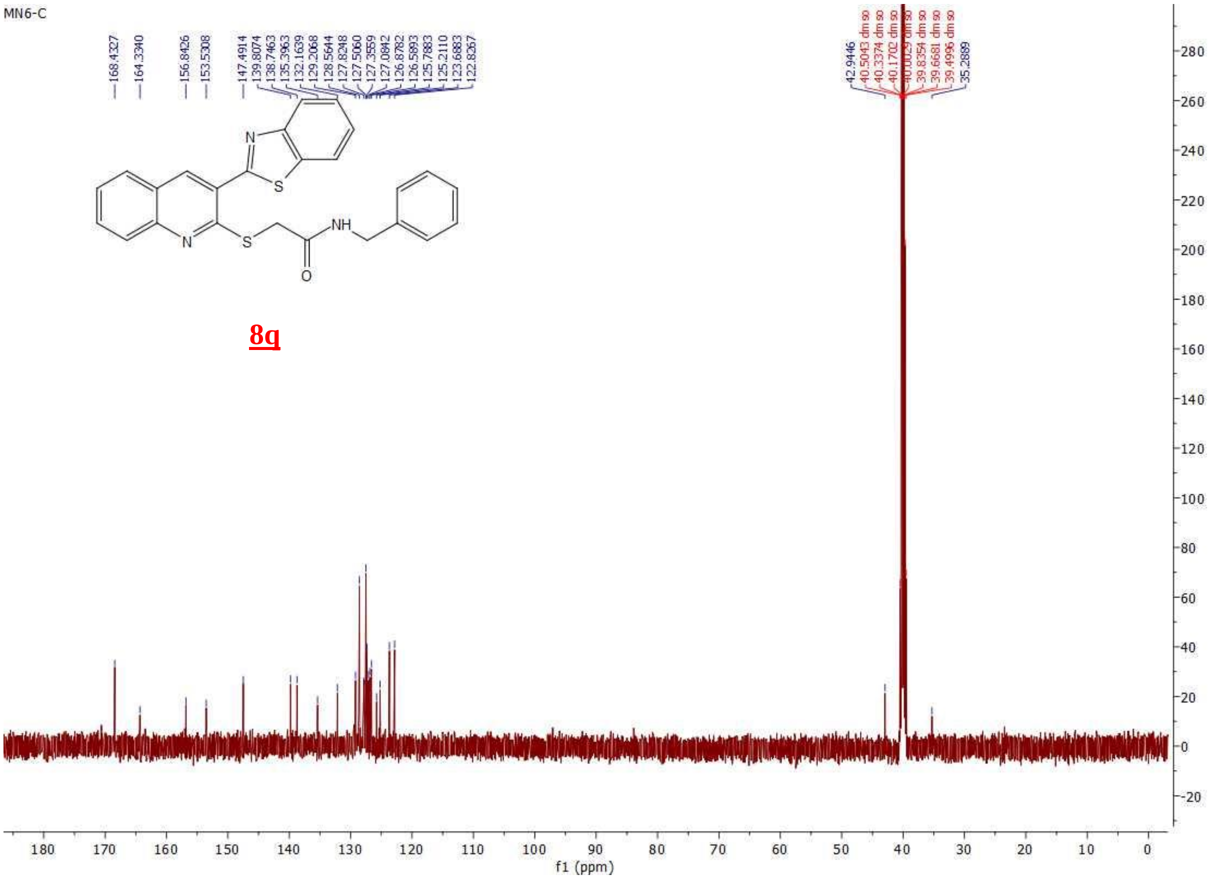
MN6-C
168.4327
164.3340
156.8426
153.5308
147.4914
139.8074
138.7463
135.3963
132.1639
129.2068
128.5644
127.8248
127.5060
127.3559
127.0842
126.8782
126.5893
125.7883
125.2110
123.6883
122.8367
42.9446
40.5043 dmso
40.3374 dmso
40.1702 dmso
39.8354 dmso
39.6681 dmso
39.4996 dmso
35.2889
N
S
N
S
NH
O
f1 (ppm)

8q

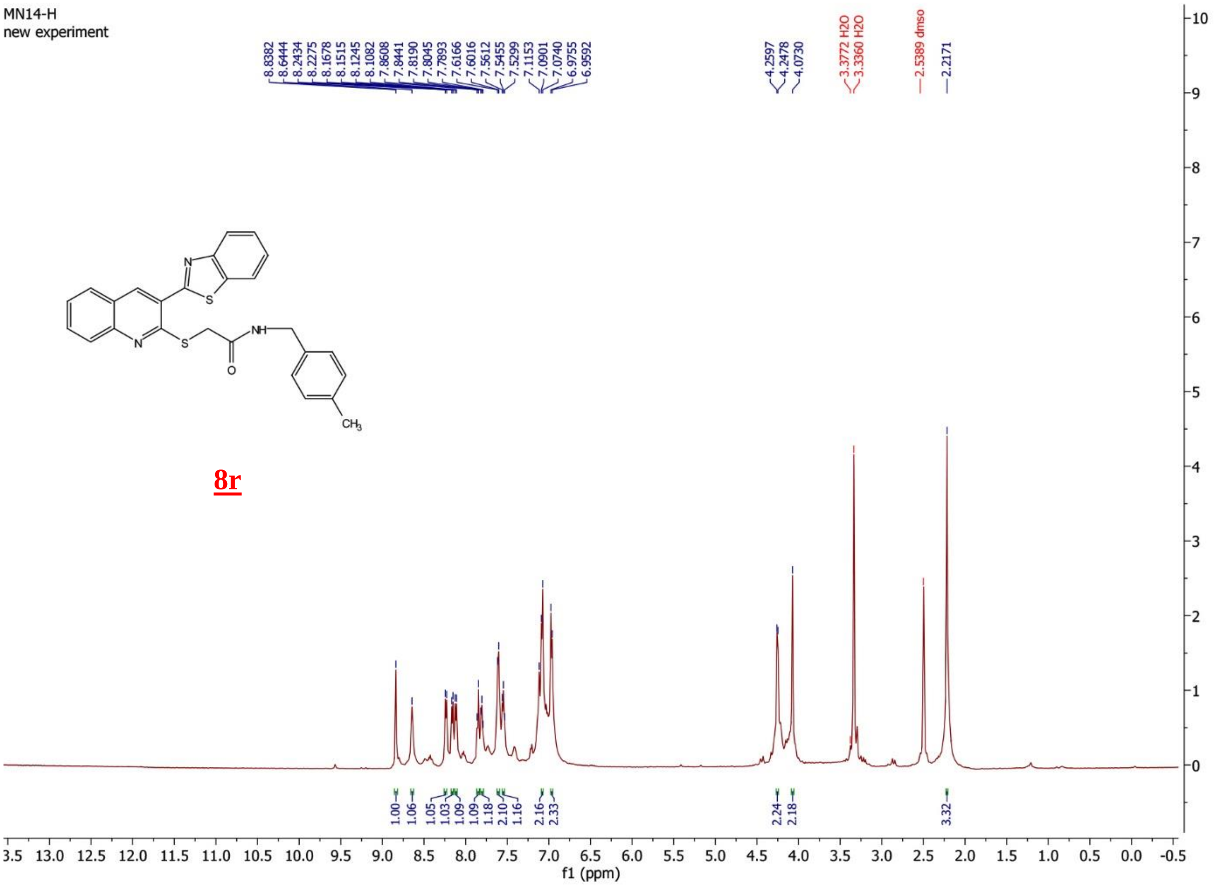

MN14-H
new experiment
8.8382
8.6444
8.2434
8.2275
8.1678
8.1515
8.1245
8.1082
7.8608
7.8441
7.8190
7.8045
7.7893
7.6166
7.6016
7.5612
7.5455
7.5299
7.1153
7.0901
7.0740
6.9755
6.9592
4.2597
4.2478
4.0730
3.3772 H2O
3.3360 H2O
2.5389 dmso
2.2171
N
S
N
S
NH
O
CH3
1.00
1.06
1.05
1.03
1.09
1.09
1.18
2.10
1.16
2.16
2.33
2.24
2.18
3.32
f1 (ppm)

8r

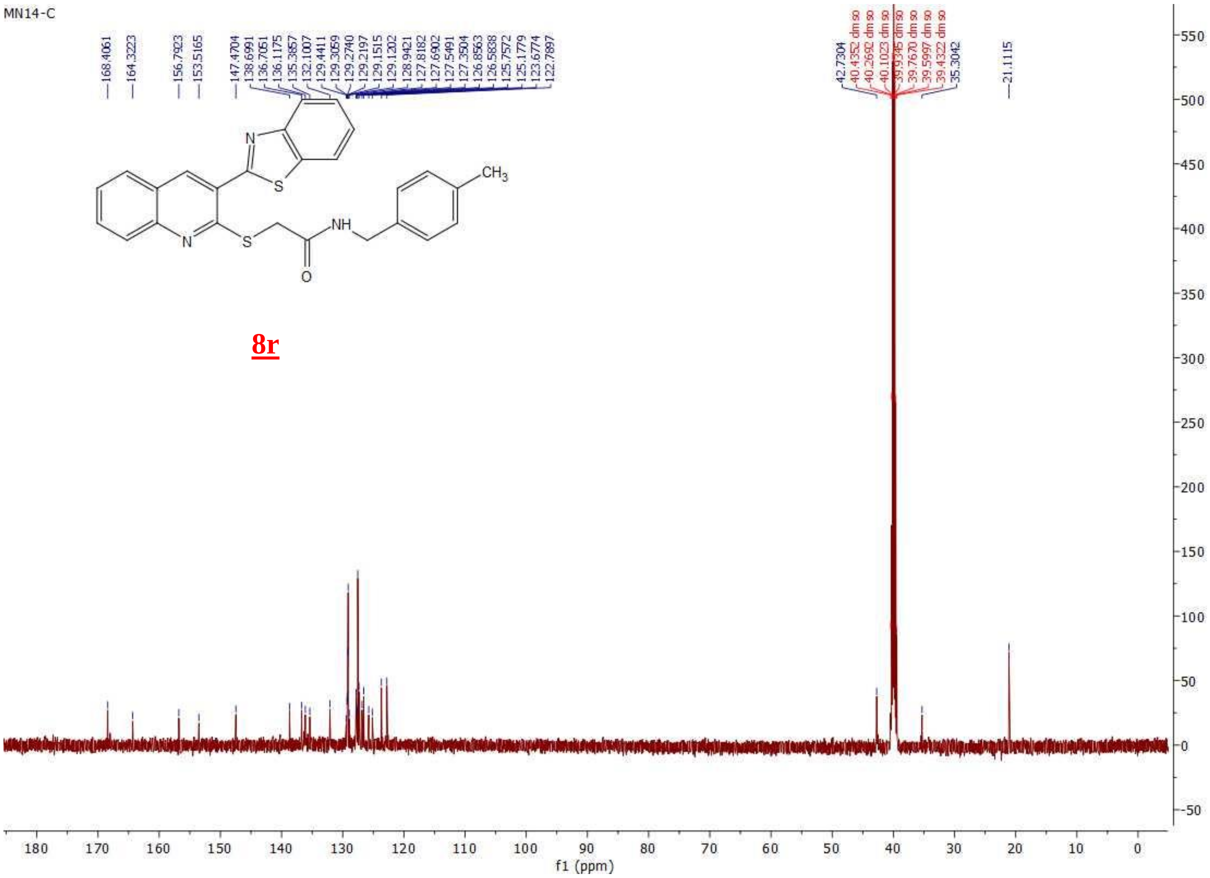
MN14-C
168.4061
164.3223
156.7923
153.5165
147.4704
138.6991
136.7051
136.1175
135.3857
132.1007
129.4411
129.3059
129.2740
129.2197
129.1515
129.1202
128.9421
127.8182
127.6902
127.5491
127.3504
126.8563
126.5838
125.7572
125.1779
123.6774
122.7897
42.7304
40.4352 dmso
40.2692 dmso
40.1023 dmso
39.9345 dmso
39.7670 dmso
39.5997 dmso
39.4322 dmso
35.3042
21.1115
N
S
N
S
NH
O
CH3
f1 (ppm)

8r

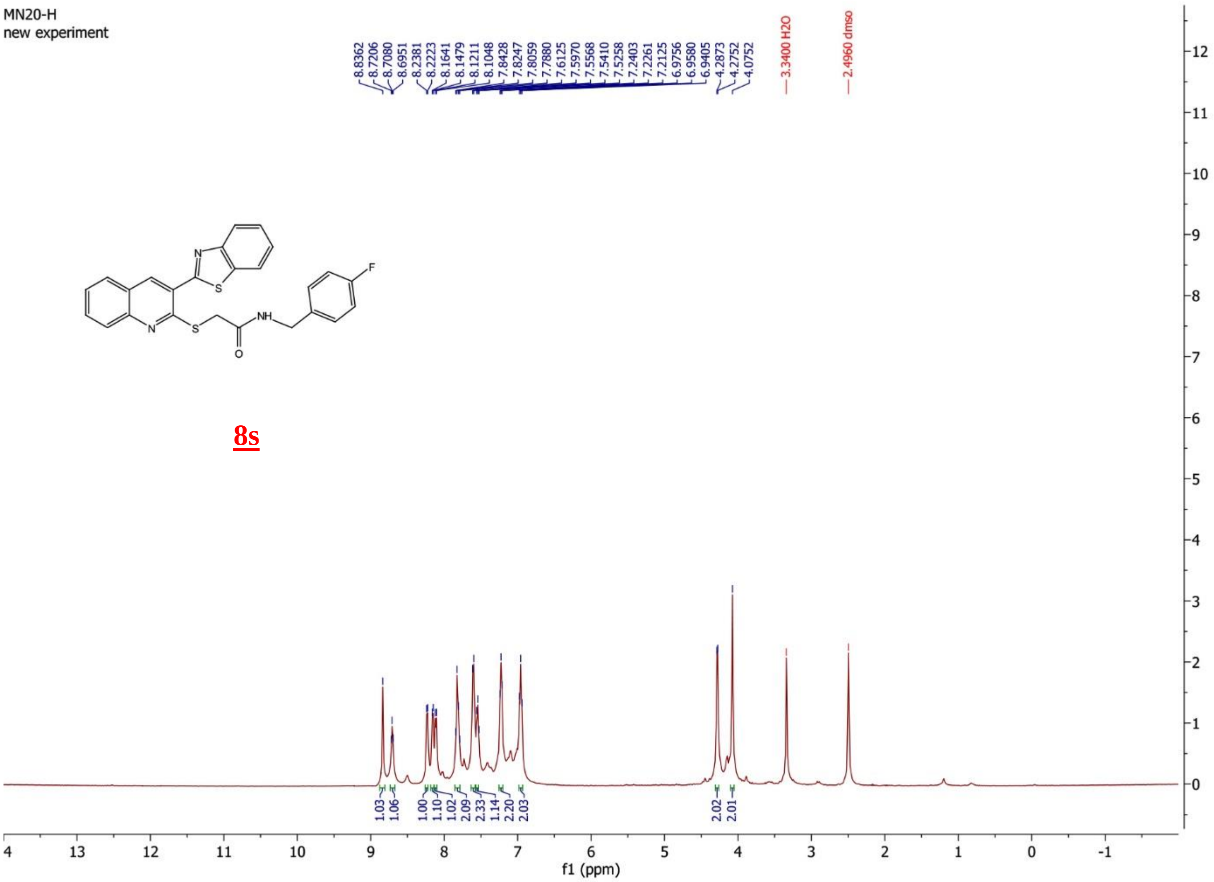

MN20-H
new experiment
8.8362
8.7206
8.7080
8.6951
8.2381
8.2223
8.1641
8.1479
8.1211
8.1048
7.8428
7.8247
7.8059
7.7880
7.6125
7.5970
7.5568
7.5410
7.5258
7.2403
7.2261
7.2125
6.9756
6.9580
6.9405
4.2873
4.2752
4.0752
3.3400 H2O
2.4960 dmso
N
S
F
NH
N
S
O
1.03
1.06
1.00
1.10
1.02
2.09
2.33
1.14
2.20
2.03
2.02
2.01
f1 (ppm)

8s

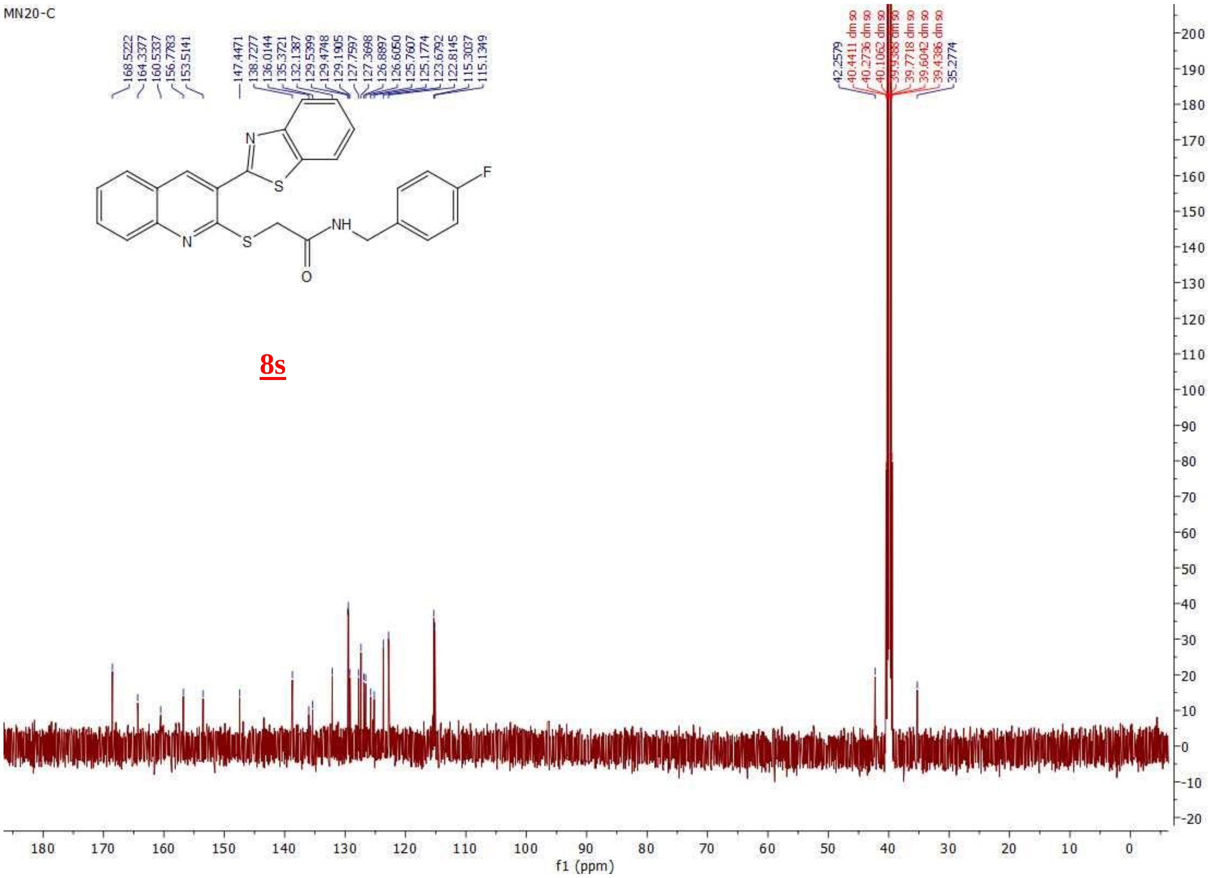
MN20-C
168.5222
164.3377
160.5337
156.7783
153.5141
147.4471
138.7277
136.0144
135.3721
132.1387
129.5399
129.4748
129.1905
127.7597
127.3698
126.8897
126.6050
125.7607
125.1774
123.6792
122.8145
115.3037
115.1349
42.2579
40.4411 dmso
40.2736 dmso
40.1062 dmso
39.9388 dmso
39.7718 dmso
39.6042 dmso
39.4386 dmso
35.2774
N
S
N
S
NH
O
F
f1 (ppm)

8s

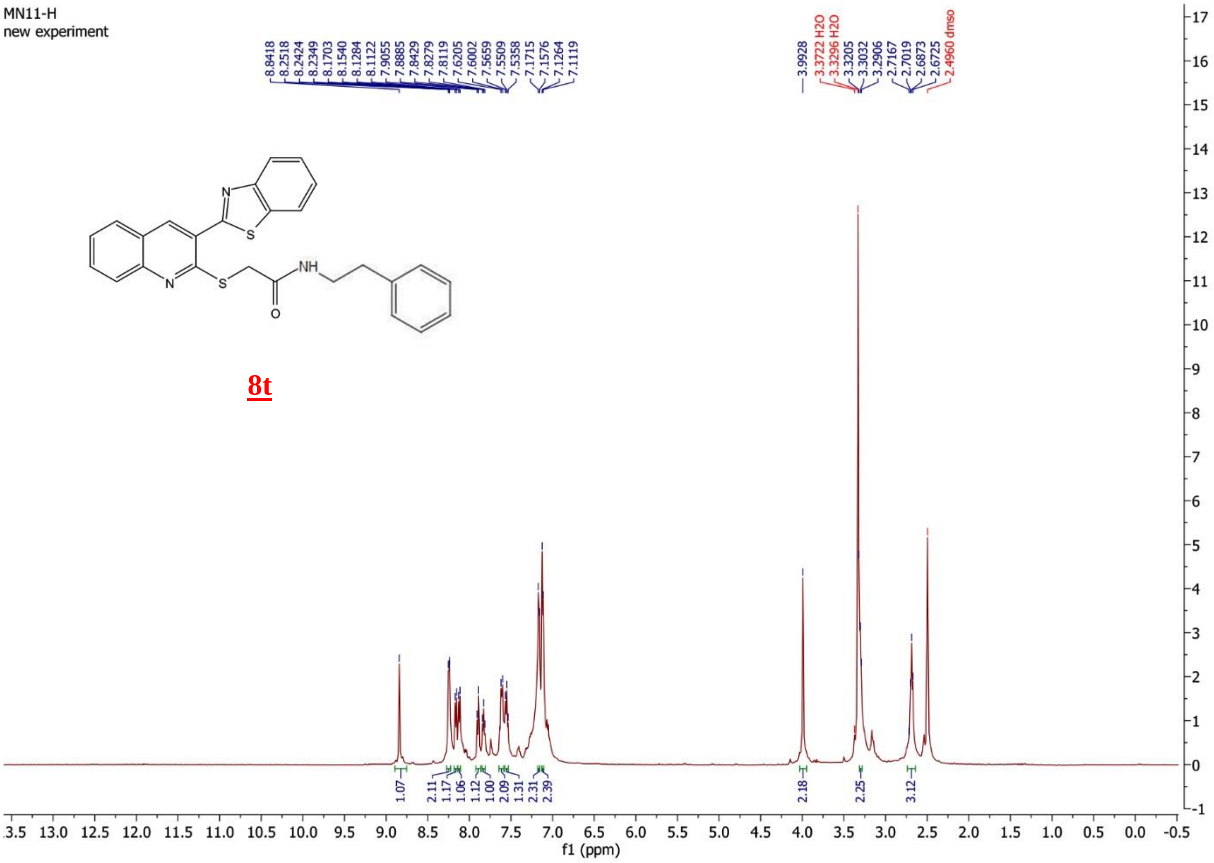
MN11-H
new experiment
8.8418 8.2518 8.2424 8.2349 8.1703 8.1540 8.1284 8.1122 7.9055 7.8885 7.8429 7.8279 7.8119 7.6205 7.6002 7.5659 7.5509 7.5358 7.1715 7.1576 7.1264 7.1119
3.9928 3.3722 H2O 3.3296 H2O 3.3205 3.3032 3.2906 2.7167 2.7019 2.6873 2.6725 2.4960 dmso
8t
1.07 2.11 1.17 1.06 1.12 1.00 2.09 1.31 2.31 2.39 2.18 2.25 3.12
f1 (ppm)

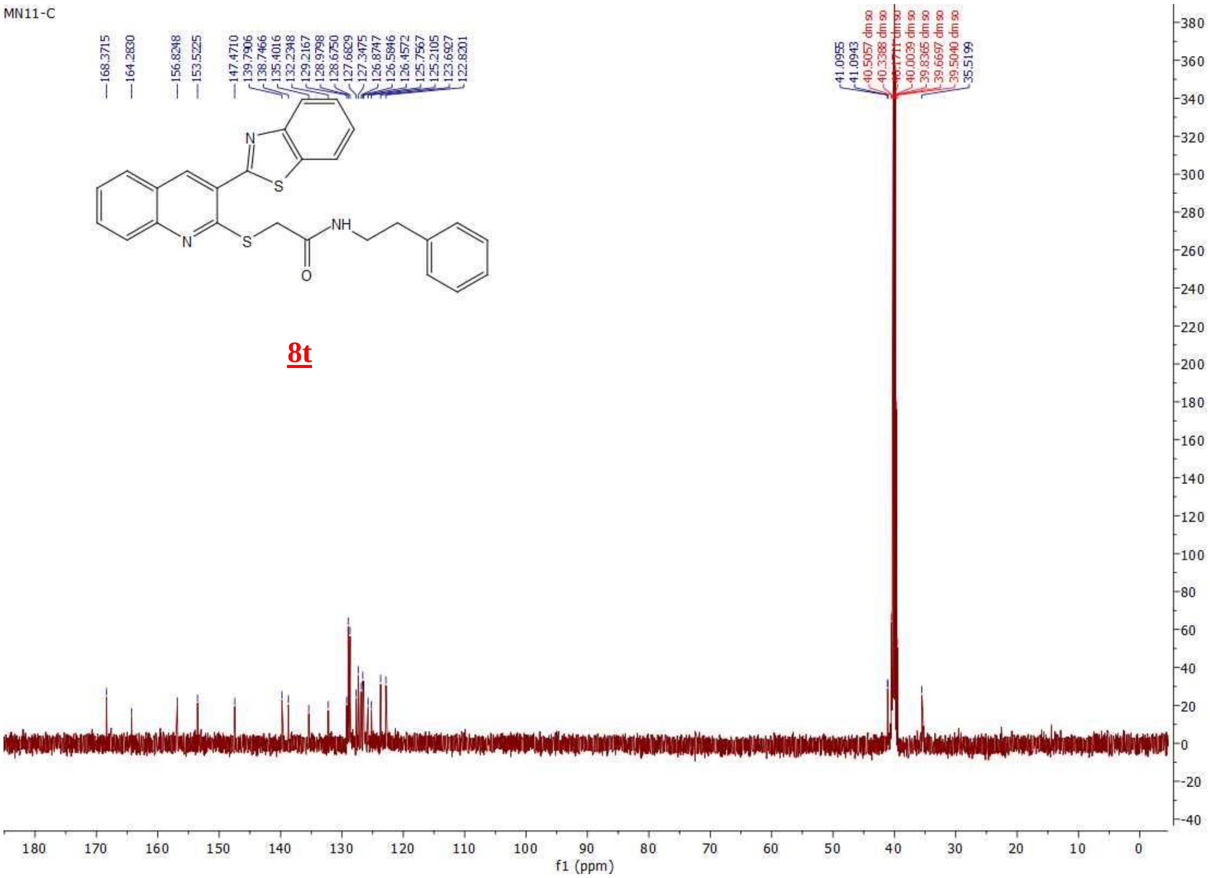
MN11-C
168.3715
164.2830
156.8248
153.5225
147.4710
139.7906
138.7466
135.4016
132.2348
129.2167
128.9798
128.6750
127.6829
127.3475
126.8747
126.5846
126.4572
125.7567
125.2105
123.6927
122.8201
41.0955
41.0943
40.5057 dmso
40.3388 dmso
dmso
40.0039 dmso
39.8365 dmso
39.6697 dmso
39.5040 dmso
35.5199
N
S
NH
O
8t
f1 (ppm)
